# Noninvasive monitoring technologies to identify discomfort and distressing symptoms in persons with limited communication at the end of life: a scoping review

**DOI:** 10.1186/s12904-024-01371-0

**Published:** 2024-03-21

**Authors:** Jingyuan Xu, Hanneke J. A. Smaling, Jan W. Schoones, Wilco P. Achterberg, Jenny T. van der Steen

**Affiliations:** 1 Department of Public Health and Primary Care, Leiden University Medical Center, Hippocratespad 21, Gebouw 3, Postzone V0-P, P.O. Box 9600, 2300 RC Leiden, The Netherlands; 2 University Network for the Care Sector Zuid-Holland, Leiden University Medical Center, Leiden, The Netherlands; 3 Directorate of Research Policy, Leiden University Medical Center, Leiden, The Netherlands; 4 Department of Primary and Community Care, and Radboudumc Alzheimer Center, Radboud university medical center, Nijmegen, The Netherlands

**Keywords:** Palliative care, Dementia, Information technology, Medical informatics

## Abstract

**Background:**

Discomfort and distressing symptoms are common at the end of life, while people in this stage are often no longer able to express themselves. Technologies may aid clinicians in detecting and treating these symptoms to improve end-of-life care. This review provides an overview of noninvasive monitoring technologies that may be applied to persons with limited communication at the end of life to identify discomfort.

**Methods:**

A systematic search was performed in nine databases, and experts were consulted. Manuscripts were included if they were written in English, Dutch, German, French, Japanese or Chinese, if the monitoring technology measured discomfort or distressing symptoms, was noninvasive, could be continuously administered for 4 hours and was potentially applicable for bed-ridden people. The screening was performed by two researchers independently. Information about the technology, its clinimetrics (validity, reliability, sensitivity, specificity, responsiveness), acceptability, and feasibility were extracted.

**Results:**

Of the 3,414 identified manuscripts, 229 met the eligibility criteria. A variety of monitoring technologies were identified, including actigraphy, brain activity monitoring, electrocardiography, electrodermal activity monitoring, surface electromyography, incontinence sensors, multimodal systems, and noncontact monitoring systems. The main indicators of discomfort monitored by these technologies were sleep, level of consciousness, risk of pressure ulcers, urinary incontinence, agitation, and pain. For the end-of-life phase, brain activity monitors could be helpful and acceptable to monitor the level of consciousness during palliative sedation. However, no manuscripts have reported on the clinimetrics, feasibility, and acceptability of the other technologies for the end-of-life phase.

**Conclusions:**

Noninvasive monitoring technologies are available to measure common symptoms at the end of life. Future research should evaluate the quality of evidence provided by existing studies and investigate the feasibility, acceptability, and usefulness of these technologies in the end-of-life setting. Guidelines for studies on healthcare technologies should be better implemented and further developed.

**Supplementary Information:**

The online version contains supplementary material available at 10.1186/s12904-024-01371-0.

## Introduction

Discomfort and distressing symptoms in the last two weeks of life are common [1–6]. Based on reviews of people dying with dementia and cancer, many may experience pain (12%-96%), difficulty breathing (8%-83%), fatigue (22%-93%), incontinence (32%-65%), and anxiety (6%-57%) [2, 6]. Studies in intensive care units (ICU), hospital general wards, nursing homes, and among the general public reported similar percentages of these symptoms, with the addition of agitation (12%—71%) [4, 5, 7], sleep disturbances (18%—56%) [7, 8], nausea (9%—59%) [4, 8], delirium (75%—91%) [3, 9, 10], fever (16%—61%), and [4, 8] pressure sores (16%—42%) [3, 4]. To relieve health-related suffering at the end of life, which is a core task of palliative care [11], it is essential to identify these distressing symptoms in a timely manner.

However, timely recognition of discomfort and distressing symptoms may be challenging at the end of life. People who are dying may have difficulties expressing their discomfort due to fatigue, sedation, delirium, cognitive impairment, loss of consciousness or communication problems such as aphasia [6, 12, 13]. In current practice, the identification of discomfort and distressing symptoms relies upon observations of professional caregivers and family [14]. However, observation may not be the most accurate measurement of discomfort [15, 16], and continuous observation of discomfort by staff is not feasible [17], so alternatives are needed. Implementing noninvasive monitoring technologies may aid in the early detection of discomfort and distressing symptoms at the end of life.

Various technologies that monitor distressing symptoms have already been developed, for example, actigraphy to monitor agitation [18], systems to detect stress based on facial expressions [19], and various devices to monitor nociception [20]. In addition to directly monitoring distressing symptoms, monitoring technologies such as the bispectral index (BIS) may also help detect discomfort by monitoring the level of consciousness of people who receive palliative sedation due to refractory distress [21–23]. However, knowledge about which technologies may be useful in the end-of-life context is lacking because most of these technologies have not been specifically developed for this setting, and previous reviews mainly focused on populations that were still mobile [24–27] and could self-report their symptoms [28, 29].

This review aims to generate an overview of noninvasive monitoring technologies that may be applied to people with limited communication at the end of life to identify discomfort and distressing symptoms. The results may provide a useful reference for clinicians who consider implementing technology in the end-of-life care of people with limited communication. Our main research question was: What noninvasive monitoring technologies are described in the literature that may be useful in identifying discomfort and distressing symptoms in persons with limited communication at the end of life? The subquestions were as follows: 1) What noninvasive monitoring technologies are available or under development that identify discomfort and distressing symptoms which can occur at the end of life, 2) What is known about the clinimetric properties (e.g., validity, reliability, sensitivity, specificity, responsiveness) of these technologies, and 3) What is known about the acceptability and feasibility of these technologies for persons with limited communication?

## Methods

A scoping review was chosen since the goal was to provide a broad view of technologies to identify discomfort and distressing symptoms in persons with limited communication at the end of life rather than to generate clinical guidance for a specific technology [30, 31]. This review was conducted in accordance with the Joanna Briggs Institute methodology for scoping reviews and reporting followed the Preferred Reporting Items for Systematic Reviews and Meta-analyses extension for scoping review (PRISMA-ScR) checklist [32]. The protocol for this scoping review was registered in the Open Science Framework (24 February 2022, Registration number: E6M8B) [33].

### Search methods

Manuscripts were included if they described a noninvasive technology that may be used to continuously (i.e., for at least 4 h) monitor discomfort and distressing symptoms in people at the end of life. The technology needed to serve as a direct or indirect measure of discomfort, potentially distressing symptoms, or level of consciousness. In studies with human subjects, participants must be at least 18 years old. The manuscript had to be written in English, Dutch, German, French, Japanese, or Chinese. The manuscript was excluded if the monitoring technology fully relied on self-report, was applied in the study to all participants during an invasive procedure only, was applied in infants or children only, it was an animal study or a review, no information on the technology was provided, or if the full text article was not available. Technologies that were judged by the screening researchers as not applicable to bed-ridden persons were also excluded, for example technologies that require input from a GPS tracker or step counts. People with limited communication at the end of life include persons who have difficulties expressing their discomfort due to fatigue, sedation, cognitive impairment, or communication problems (e.g. aphasia) [12]. This is operationalized in the search by also including populations in similar situations as people with limited communication at the end of life, where they are bedridden and noncommunicative, for example people with dementia, aphasia, profound intellectual disability, and people in critical care. Noninvasive is defined in this review as no penetration in the body of the person with solid material, while weak electric currents, weak laser lights or ultrasounds may be used [34]. 

On 16 March 2021, a search was conducted in PubMed, MEDLINE (OVID), Embase (OVID), Emcare (OVID), Web of Science, Cochrane Library, PsycINFO (EbscoHOST), Academic Search Premier (EbscoHOST), and Google Scholar. On 23 February 2023, an update search was performed in the same databases. In cooperation with a trained librarian (JWS), a detailed search strategy was composed. The query consisted of the combination of the following three concepts:

- death, end-of-life phase, terminal care, elderly, critical care, profound disability, bedridden persons, nonmobile persons, dementia, aphasia
- activity monitors, activity trackers
- (dis-)comfort, distress, agitation, pain assessment, pressure ulcers, quality of life, breathing difficulty, anxiety, sleep disturbances, fatigue, nausea, delirium, fever, incontinence, sedation depth

For these concepts, all relevant keyword variations were used, not only keyword variations in the controlled vocabularies of the various databases but also the free text word variations of these concepts. The search strategy was optimized for all consulted databases, taking into account the differences in the various controlled vocabularies as well as the differences in database-specific technical variations (e.g., the use of quotation marks). The complete search strategy is available as an appendix to the registered protocol [33]. After data extraction, six experts were consulted for additional, potentially missing information about new technologies under development. The experts were from the Interdem Taskforce Assistive Technologies and professional network of the authors.

### Source of evidence screening and selection

After removing duplicates, each title and abstract was independently screened by two researchers. Full-text articles were retrieved after the first screening round, and each article was assessed for eligibility by two independent reviewers. Consensus meetings were held to discuss discrepancies.

### Data extraction

Data extraction was performed using a form developed and pilot-tested by the research team. Extracted data were checked by a second researcher, and regular consensus meetings were held to discuss doubts and discrepancies. The following data were extracted from the included articles: author, year of publication, language, country, participants, type of technology, model and brand, and indicators of discomfort monitored by the technology. For studies that described the development of a technology, the aim of the study and the details of the technology (e.g., sensors, parameters) were extracted. For studies mentioning clinimetrics, acceptability, or feasibility of the technology, this information was also extracted, together with the study design and other instruments that were used in the study. The extracted data were then synthesized into tables and narrative summaries for each type of technology. An often used threshold for acceptable clinimetrics is at least 70% for sensitivity, specificity, and area under the curve (AUC) [35, 36].

## Results

**Fig. 1:**
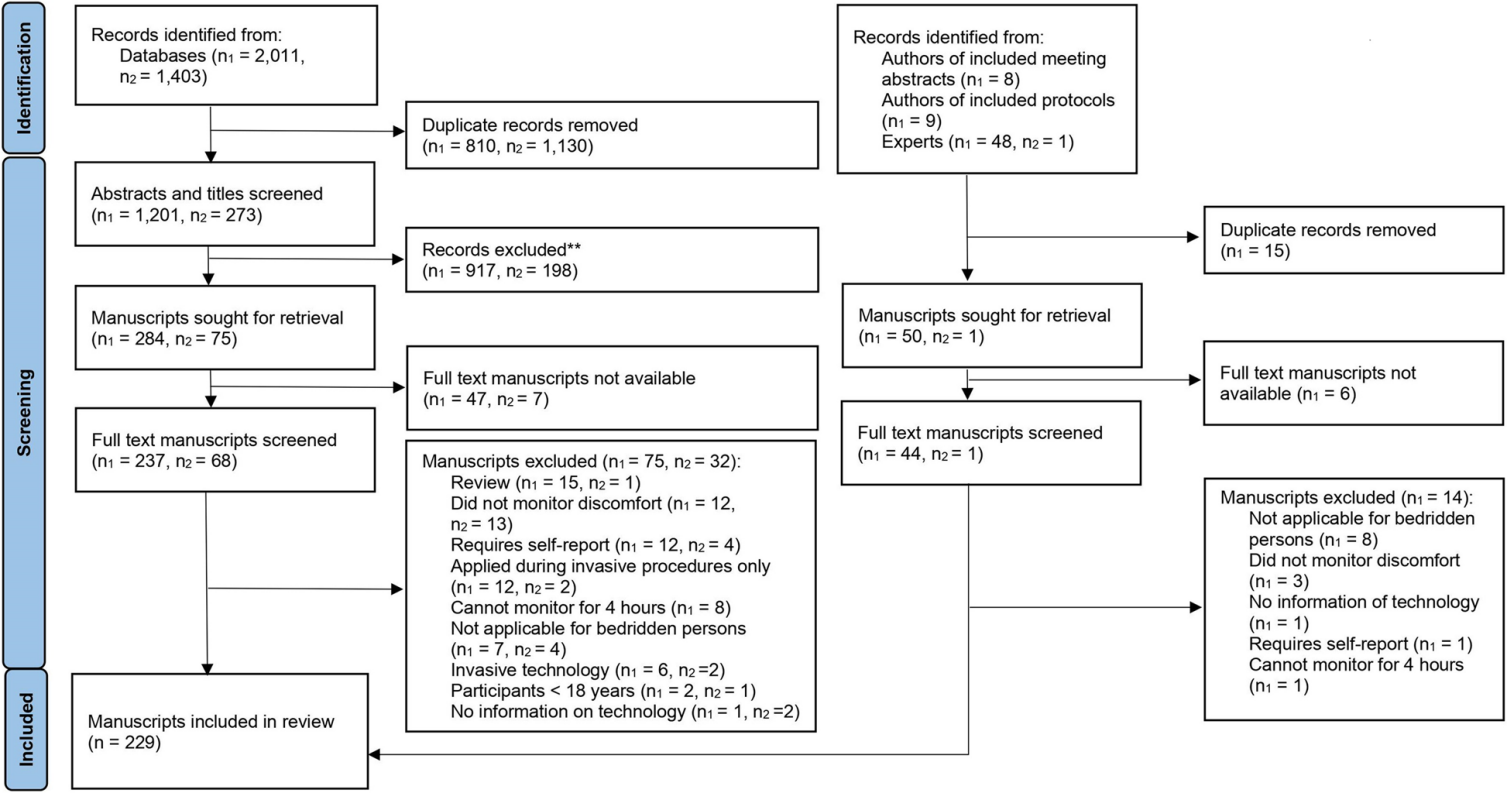
Preferred Reporting Items for Systematic Reviews and Meta-analyses (PRISMA) flow diagram. Note: n1= first search in 2021, n2 = update search in 2023

The flow chart of the screening process is presented in Fig. 1. A total of 229 manuscripts were included. Table 1 shows that most manuscripts were written in English by researchers in North America or Europe. The number of publications on this topic has shown exponential growth over the past 3 decades. Based on the bodily functions and signals that are measured, the monitoring technologies included in this review were grouped into 8 categories, of which actigraphy and multimodal systems such as polysomnography accounted for more than half of the included manuscripts. Table 2 lists the identified monitoring technologies for each indicator of discomfort. Multiple technologies measured sleep, pain, and agitation. No technologies monitored difficulty breathing, nausea, or fever. Many monitored sleep disordered breathing, but not the subjective experience of difficulty breathing.

**Table 1:** Typology of the included manuscripts

	**Number of manuscripts**	**Percentage**
**Languages**		
English	220	96%
Chinese	4	2%
Dutch	2	1%
Japanese	2	1%
German	1	< 1%
**Countries**		
United States of America	63	28%
Canada	13	6%
Netherlands	12	5%
Italy	11	5%
France	9	4%
Germany	9	4%
Japan	9	4%
Australia	8	3%
United Kingdom	8	3%
Belgium	7	3%
China	7	3%
South Korea	7	3%
Finland	5	2%
Spain	5	2%
Switzerland	3	1%
Brazil	2	1%
Denmark	2	1%
India	2	1%
Greece	2	1%
Mexico	2	1%
New Zealand	2	1%
Portugal	2	1%
Singapore	2	1%
Other countries	8	3%
Two or more countries^a^	29	13%
**Year of publication**		
1991–1995	4	2%
1996–2000	13	6%
2001–2005	15	7%
2006–2010	31	14%
2011–2015	41	18%
2016–2020	79	25%
2021-2023^b^	46	20%
**Monitoring technologies**		
Actigraphy	57	25%
Brain activity monitors	25	11%
Bispectral Index (BIS)	15	
Other Electroencephalography (EEG)-based^c, d^	9	
Sedation monitor (concept)	1	
Electrocardiography (ECG)^c^	12	5%
Electrodermal activity (EDA) monitor	8	3%
Surface electromyogram (sEMG)	2	1%
Incontinence sensors	11	5%
Multi-modal systems^e^	96	42%
Polysomnography (PSG)	75	
With environmental sensors	6	
Other multi-modal systems	14	
Non-contact monitoring systems ^f^	21	9%
Pressure mats	9	
Video/facial expression analysis	4	
Infrared	2	
Radar	3	
Multiple sensors	3	
**Aim of studies**^**g**^		
No technology-related aims (the technology is applied as standard measurement)	91	39%
Development of the technology	38	17%
Feasibility/pilot study of the technology	51	22%
Validation of the technology	65	28%

Total number of included manuscripts = 229

^a^The main countries involved were the USA (11 manuscripts), China (8 manuscripts), Finland (6 manuscripts), and the Netherlands (6 manuscripts). The countries involved in specific manuscripts can be found in etables 1–3 in the supplements

^b^Manuscripts published before March 13, 2023 were included

^c^3 manuscripts used both Electroencephalography and Electrocardiography to monitor people under sedation

^d^Other electroencephalogram-based technologies refer to traditional EEGs and other technologies which use EEG electrodes

^e^Multi-modal systems use a combination of different technologies within one integrated system or algorithm

^f^Non-contact monitoring systems use sensors that do not have direct contact with the person being monitored

^g^16 articles had multiple aims

**Table 2:** Summary of monitoring technologies per indicator of discomfort

Indicator of discomfort	Types of monitoring technology^a^
Vital signs	Multi-modal systems^b^ – other multi-model systems
	Non-contact monitoring systems^c^ – radar
Arrhythmia	Electrocardiography (ECG)
Consciousness/depth of sedation	Actigraphy
	Brain activity monitors—bispectral index scale (BIS)
	Brain activity monitors – other electroencephalogram (EEG)-based technologies
Sleep, including sleep disordered breathing	Actigraphy
	Brain activity monitors—BIS
	Brain activity monitors – other EEG-based technologies
	ECG
	Multi-modal systems^b^ – PSG
	Multi-modal systems^b^ – with environmental sensors
	Multi-modal systems^b^ – other multi-model systems
	Non-contact monitoring systems^c^ – pressure mats
	Non-contact monitoring systems^c^ – infrared
	Non-contact monitoring systems^c^ – radar
	Non-contact monitoring systems^c^ – multiple sensors
(Risk of) pressure ulcers	Actigraphy
	Non-contact monitoring systems^c^—pressure mats
Urinary incontinence	Incontinence sensors
	Multi-modal systems^b^ – with environmental sensors
Pain	Brain activity monitors—BIS
	Brain activity monitors – other EEG-based technologies
	ECG
	Electrodermal activity (EDA) monitor
	Surface electromyogram (sEMG)
	Multi-modal systems^b^ – other multi-model systems
	Non-contact monitoring systems^c^ – video/facial expression analysis
Agitation	Actigraphy
	Multi-modal systems^b^ – with environmental sensors
	Multi-modal systems^b^ – other multi-model systems
	Non-contact monitoring systems^c^ – video/facial expression analysis
	Non-contact monitoring systems^c^ – multiple sensors
Anxiety	ECG
	Multi-modal systems^b^ – with environmental sensors
	Multi-modal systems^b^ – other multi-model systems
Stress	Actigraphy
	Brain activity monitors – other EEG-based technologies
	ECG
	EDA monitor
	Multi-modal systems^b^ – other multi-model systems
Significant moments	Multi-modal systems^b^ – other multi-model systems
Emotions	Non-contact monitoring systems^c^ – video/facial expression analysis
(Motor subtypes of) delirium	Actigraphy
	Brain activity monitors—BIS
	Brain activity monitors – other EEG-based technologies
	Multi-modal systems^b^ – with environmental sensors
	Multi-modal systems^b^ – other multi-model systems
Thermal comfort	Non-contact monitoring systems^c^ – infrared
Mental fatigue	Multi-modal systems^b^ – other multi-model systems

^a^Cateogries and subcategories (if applicable) of technologies are presented

^b^Multi-modal systems use a combination of different technologies within one integrated system or algorithm

^c^Non-contact monitoring systems use sensors that do not have direct contact with the person being monitored

We briefly illustrate each type of monitoring technology and the main findings from the studies regarding the clinimetrics (see Supplement 1), acceptability, and feasibility (see Supplement 2). Supplement 3 summarizes manuscripts that did not report clinimetrics, acceptability, or feasibility, mainly because the technology was implemented as standard measurement instruments. The models and brands of the technologies, the settings and the study populations are listed in Supplements 1, 2 and 3.

### Actigraphy

Actigraphy uses accelerometers to measure movements with a band or pads that are attached to the wrist, ankle, neck, mattress [37], or integrated in a garment [38]. The data are usually processed by built-in software. Several researchers developed their own algorithms with machine learning to analyze the construct of interest, for example, agitation, body position or sleep/wake states, using movement data [38–40]. In approximately half of the manuscripts, actigraphy was used as a standard measurement for sleep. When discrepancies were observed between actigraphy results and observations or self-reports, authors of recent manuscripts favored actigraphy as a standard, objective measurement [41, 42].

#### Clinimetrics

Although there were some mixed results for different models of actigraphy, showing that it could underestimate sleep time in the ICU [40] or overestimate this in people with Huntington’s or Alzheimer’s Disease compared to polysomnography [43, 44], and it could be less accurate when the participants had sleep disordered breathing [45], in general, it demonstrated acceptable sensitivity and specificity of identifying sleep–wake states in ICU patients [40], older adults in nursing homes [46] and people with dementia [47].

Actigraphy measurement could recognize agitated behaviors [39], distinguish between groups of higher and lower agitation in people with dementia in long-term care facilities or hospital [48, 49], and was correlated with observational scales for agitation such as the Cohen-Mansfield Agitation Inventory in people with dementia [49, 50]. The correlation with the Neuropsychiatric Inventory was found only in some articles [49] but not in others [48]. Among nursing home residents with dementia, actigraphy demonstrated acceptable discriminant validity compared with the Mini-Mental State Examination, which measured a different concept [51]. Most of these studies were performed in people with dementia who were still mobile. In one study among ICU patients, who may be more similar to people at the end of life, actigraphic data also correlated with observations of agitation using the Richmond Agitation Sedation Scale [52].

The devices had good precision and sensitivity when used to monitor the risk of pressure ulcers in the hospital [38] and could reduce the chances of patients developing pressure ulcers in the ICU [53]. Actigraphy could recognize stress in nursing home residents with acceptable accuracy but lower precision and sensitivity [54].

In summary, for populations that are similar to people at the end of life, actigraphy has demonstrated good clinimetrics in monitoring sleep, agitation, and risk of pressure ulcers but is not precise in recognizing stress.

#### Feasibility and acceptability

Actigraphy was feasible for multiday continuous measurement of older adults with intellectual disability [55] and people with dementia [49]. Although community-dwelling older adults found it easy to use [56], it can be confusing for some people with less cognitive capacity [57]. The battery life could also be improved [44, 56]. No manuscripts reported on the feasibility and acceptability of actigraphy in bed-ridden participants with limited communication.

### Brain activity monitors

Electronic activity of the brain can be measured by electroencephalography. It traditionally uses electrodes with gel attached to the head. Portable versions with few electrodes have been developed [58].

#### Bispectral index (BIS)

BIS is a specific type of device that uses EEG signals to measure the depth of sedation. BIS consists of a sticker on the forehead with four EEG electrodes connected to a small monitor that displays a number ranging from 0 to 100. It has been implemented in the ICU and palliative care units [21, 59].

##### Clinimetrics

Sedation indices measured by BIS correlated with those measured by standard EEG in the ICU [60]. BIS scores increased during painful procedures in patients with traumatic brain injury in the ICU [61]. It also correlated with PSG and could discriminate between deep and light sleep in healthy adults [62]; however, it was only weakly correlated with self-reported sleep quality in ICU patients [63]. BIS monitoring with density spectral was tested for the detection of delirium in hospitals. Its accuracy was comparable to that of the 3-min Diagnostic Interview for Confusion Assessment Method and was more accurate in people with dementia than in those without cognitive impairment [64].

In summary, limited research has been performed on the clinimetrics of BIS. The preliminary results showed that it may have the potential to detect pain and delirium, as well as to monitor sedation and sleep [60–64].

##### Feasibility and acceptability

In a study in the ICU, BIS was deemed not possible to implement in a quarter of the eligible participants due to delirium, other medical procedures, discomfort of the sensors or displacement of the sensors [63]. One case study suggested that BIS was helpful for nurses to titrate medication for palliative sedation in the ICU and to educate the family. It reassured the family that the patient died comfortably, and they were pleased to see BIS fluctuations when talking to the patient [65]. Thus, BIS is not always feasible, but it can be acceptable and helpful when it is.

#### Other electroencephalography (EEG)-based technologies

In recent years, EEG sensors have been developed into more user-friendly forms of dry electrodes [58], a headband [66] or an earplug [67]. In two older studies, EEG was used to objectively measure sleep [68, 69]. Two recent studies used EEG to objectively monitor the depth of sedation [16, 70].

##### Clinimetrics

Compared to PSG, EEG-based technologies showed good accuracy in determining sleep stages in healthy adults [67] and could distinguish sleep and wake in ICU patients [71]. Good AUC was reported for delirium diagnosis in the ICU [72]. Preliminary results showed differences in EEG signals during stress in healthy adults [66] and pain in people with dementia residing in long-term care settings [58].

##### Feasibility and acceptability

A portable device to measure sleep was deemed feasible in the ICU [71]. An EEG-based headband was acceptable for long-term care residents with dementia, while properly positioning the device was challenging [58]. No manuscript reported on the acceptability of other EEG-based technologies.

#### The concept of sedation monitors

##### Feasibility and acceptability

An interview study showed that family and professional caregivers considered it acceptable to use monitors during palliative sedation as an objective measurement of sedation and pain, which could reassure the family and guide titration [73].

### Electrocardiography (ECG)

ECG is the measurement of the electronic activity of the heart. Traditionally, electrodes are attached to the torso and connected to a monitor. Among the manuscripts included in this review, the number of electrodes varied between 3 and 12, and the devices could be portable [74, 75]. Heart rate data can also be extracted from smart watches [76]. ECG was used as a standard measurement for arrhythmia [77] and stress [76, 78, 79].

#### Clinimetrics

Indices derived from ECG signals correlated with self-rated pain among patients taking physical therapy after surgery [74] and sleep apnea among people with suspected sleep disordered breathing measured by standard PSG [75]. When ECG indications of pain differed from observation, a recent study with patients under palliative sedation favored the ECG measurement and concluded that it was more objective [70]. No studies reported the clinimetrics of ECG in people at the end of life.

#### Feasibility and acceptability

It was feasible to use ECG to monitor stress in the workplace [80]. After surgery, patients were more satisfied with ECG monitoring for atrial fibrillation than observation [81].

### Electrodermal activity (EDA) monitors

By placing electrodes on the skin, electrodermal activity monitors measure the skin conductance, which drops as the sweat glands are activated by the sympathetic nervous system, indicating stress [82]. It is best measured at the palms and feet, where the sweat glands are dense. An older version of the portable EDA monitor included sensors on two fingers and hardware bound to the arm [82], while newer versions can take the form of a ring [83] or a sock [84]. EDA monitors have been tested with healthy adults and people in the ICU.

#### Clinimetrics

EDA measurement has been shown to be more sensitive than vital signs and BIS measurements in detecting pain in sedated patients in the ICU [85]. Among healthy volunteers, tt had good sensitivity, specificity and accuracy in detecting stress [84, 86] and could discriminate between stress and cognitive load [82].

#### Feasibility and acceptability

EDA sensors integrated into the sock were feasible for most participants in a study with intellectual disabilities [86]. Factors that hindered the implementation were other garments, such as compression socks, and the participants removing the device.

### Surface electromyography (sEMG)

Surface electromyography monitors muscle activity by attaching electrodes on the surface of the skin. Sensors of an older version needed to be attached to a monitor, which hanged in a holster at the waist [87]. A new device integrated multiple sensors into one soft mask and used Wi-Fi to transfer data directly from the sensor nodes to the server [88]. Only two studies on sEMG were identified in this review.

#### Clinimetrics

sEMG signals at the shoulder did not seem to be different for people experiencing cervical pain from those without pain [87]. However, facial expressions monitored by sEMG were different for neutral and painful faces in the laboratory setting [88].

#### Feasibility and acceptability

No manuscripts have reported on the feasibility and acceptability of sEMG.

### Incontinence monitors

Incontinence monitors are systems to monitor urinary incontinence events so that caregivers would only need to change the incontinence materials when an event takes place. Sensors measuring moisture or temperature change are attached to or built into incontinent materials. One device integrated the sensors into the fabric of a mat on top of the mattress [89]. The more recent and commercially available sensors are usually disposable. Data can be transmitted wirelessly to a web portal or mobile app.

#### Clinimetrics

Although there seemed to be high false positive and false negative rates in earlier systems tested in the laboratory and acute care rehabilitation wards for older adults [90, 91], more recent manuscripts reported favorable results in the laboratory setting [92]. The incontinence monitors could also reduce urine leakage and the use of incontinence materials in nursing homes [93, 94]. However, some of these reports may need to be interpreted with caution since they were published by the manufacturers.

#### Feasibility and acceptability

The incontinence sensors did not seem to disturb the patients since they reported not feeling the sensors in laboratory tests [90]. An earlier system was challenging to implement due to several technical problems and the unwillingness of experienced staff to change their routine in rehabilitation wards [91]. A recent pilot study reported positive reactions of family caregivers and staff in nursing homes. They appreciated the fact that the device helped save time and reduced the occasions where the clients were disturbed unnecessarily [94].

### Multimodal systems

Systems combining different sensors that monitor different bodily functions are grouped into the category multimodal systems.

#### Polysomnography (PSG)

Standard polysomnography uses multiple parameters (EEG, electro-oculogram, EMG, ECG, oxygen saturation, body position, snoring, airflow and breathing measurements) to monitor sleep [95]. It is considered the gold standard for diagnosing disordered breathing during sleep (e.g., obstructive sleep apnea), which is how it was used in most included manuscripts. Traditionally, PSG is administered by a technician at a sleep laboratory, but portable home monitoring systems have been developed, using only some of the sensors to monitor sleep quality or breathing [96–98]. New portable systems can take the form of a headband [99], a wristband [100], a collar around the neck [101], or a ring [102]. PSG has been used in diverse populations, including adults with sleep problems and nursing home residents with dementia.

##### Clinimetrics

Portable PSG systems were reported to have good reliability, sensitivity and specificity in diagnosing obstructive sleep apnea among outpatients with heart or lung problems compared with standard PSG [96, 101, 103, 104] but may produce false positives when compared with clinician judgment in the post anesthesia care unit [105] or may produce false negatives when compared with traditional PSG among people using positive airway pressure [106]. It was recommended to lower the cutoff score when using portable PSG to detect hypopnea events [106]. Minute ventilation, measuring the tidal volume and respiration rates with only two chest leads, was effective in detecting respiratory depression in the post anesthesia care unit [98]. With the help of machine learning models, new potable PSG systems have also demonstrated accuracy in measuring sleep stages in healthy adults [102]. In summary, portable PSG systems had good clinimetrics in monitoring sleep disordered breathing and sleep stages.

##### Feasibility and acceptability

Portable PSG systems may have occasional technical failure [107–109] but were evaluated as easy to use, comfortable, and did not interfere with sleep among healthy adults, those with sleep apnea, and people with bipolar disorder [109–111].

#### With environmental sensors

In recent years, environmental sensors to assess light, sound, humidity, and barometric pressure have been combined with actigraphy to monitor agitation and uncover its environmental determinants in people with dementia in their homes or institutions [112–114]. Sometimes cameras [114, 115], door sensors, pressure mats, and EDA sensors [114] were also used in the systems. Other systems monitored anxiety [116], delirium [115], sleep, and urinary incontinence [117]. In most of the included manuscripts, data from the various sensors were not yet integrated in a system and needed to be synchronized and processed by the researchers. These systems have been tested with people with dementia and older adults in the ICU.

##### Clinimetrics

Factors associated with agitation or delirium were identified. For people with dementia, environmental factors (i.e., temperature, pressure, and humidity) strongly correlated with agitation annotated by nurses or family caregivers, although the predictors were specific to each participant [113]. Another study indicated that the combination of actigraphy, skin temperature, and EDA data predicted agitation the best for people with dementia [114]. In the ICU, older adults with delirium differed from those without delirium in facial expressions, movements, postures, visitation frequency, environmental light and sound at night [115].

##### Feasibility and acceptability

Studies concluded that their systems were feasible for monitoring people with dementia at a rehabilitation institute [114] and critically ill patients in the ICU [115]. The challenges were Wi-Fi connections [112], trouble-shooting for the installation via the phone [117], and that people with dementia can take off the sensors when agitated [118].

#### Other multimodal systems

Several systems combined ECG, respiration monitors, EDA, sEMG, skin temperature, and breath temperature to monitor the activation of the sympathetic nervous system, which was indicative of pain [119], mental stress [120], anxiety [121], agitation [122], or significant moments [123] in different studies. In most studies, the sensors were implemented separately, but several studies integrated the sensors into a chest belt [122], a wristband [124], or a system worn on the fingertip [123]. New algorithms for analyzing the data were proposed [125]. These multimodal systems have mainly been tested in the laboratory with healthy adults [126, 127] but also with older adults with or without dementia [122, 123].

##### Clinimetrics

The multimodals systems could produce accurate measurements of physiological signals, such as ECG, photoplethysmography and respiration signals, changes in heart rate, skin temperature, and EDA, similar to conventional instruments in adults and older adults tested in the laboratory setting [122, 127]. Compared with self-report, the systems had an acceptable accuracy in classifying pain into three severity categories in healthy adults in the laboratory [119], had good clinimetrics in detecting moderate to severe pain among patients in the ICU, but the results were not optimal when the pain was milder [128]. They could detect significant moments [123], agitation and aggression in people with dementia [124, 129], and correlated with mental fatigue [130] and stress in healthy adults [120, 126]. One system could indicate anxiety with high precision but low specificity compared to self-report and observation in healthy adults [121]. In summary, multimodal systems had good clinimetrics in detecting pain and had some preliminary positive results in monitoring agitation and stress.

##### Feasibility and acceptability

A chest belt integrating EDA, heart rate, and skin temperature measurements was rated by community-dwelling older adults with a score of 2.7 out of 3 for appearance and 2.8 out of 3 for comfortability [122]. In another study with healthy adults in the laboratory, the authors reported EEG signals to be poor when participants moved [121].

### Noncontact monitoring systems

#### Pressure mats

Pressure mats are mattresses or films with integrated sensors (e.g., piezoelectric sensors [131]) to measure pressure. They do not need to have direct contact with the person since they can be used under a mattress topper or sheet. Pressure mats can be used to measure the body position and movement on the bed, which can be useful for preventing pressure ulcers [132]. Micromovements measured by pressure mats can be used to monitor sleep stages and sleep disordered breathing [133, 134]. Pressure mats have been tested with healthy adults in the laboratory and were implemented as standard measurements in nursing home residents with dementia, people with sleep apnea, and patients in the ICU and intermediate care units.

##### Clinimetrics

While older models of pressure mats needed the addition of a percutaneous oxygen saturation sensor to accurately detect sleep disordered breathing [135], a new model was very accurate on its own compared to PSG in the laboratory among outpatients with sleep problems [131]. Clinimetrics of the pressure mats in measuring other indicators of discomfort were not reported in the included manuscripts.

##### Feasibility and acceptability

A study in 2009 showed that pressure mats were feasible to use in the ICU for 48 h [136].

#### Video/facial expression analysis

Several algorithms have been developed to recognize agitation, emotions, and pain by analyzing videos taken by commercially available cameras. Using remote photoplethysmography (rPPG), some systems can also estimate heart rhythm based on the change in light transmission and reflection of the skin due to changes in blood flow [137].

##### Clinimetrics

The systems could detect faces and facial expressions most of the time, even when they were moving, as in simulated videos [138], or partially covered, as during group activity with people with Mild Cognitive Impairment [139]. They could recognize grimaces in simulated videos and emotions in people with mild cognitive impairment and were moderately to highly correlated with manual scoring of pain based on facial expressions in community-dwelling older adults and nursing home residents with dementia [137, 140].

##### Feasibility and acceptability

No manuscripts have reported on the feasibility and acceptability of these systems.

#### Infrared

Infrared sensors register the infrared waves emitted by warm objects. It can be used to monitor skin temperature and motion, which are further analyzed by researchers to indicate thermal comfort [141] and sleep stages [142]. The sensor can be installed in front of the participants or above their beds. The systems have been tested in healthy adults and nursing home residents.

##### Clinimetrics

A model to analyze the thermal comfort of nursing home residents was reliable. A lower temperature of the nose indicated feeling cold [141]. Compared to a commercially available bed sensor, the infrared system was able to detect waking states better than other stages of sleep in healthy adults [142].

##### Feasibility and acceptability

No manuscripts have reported on the feasibility and acceptability of these systems.

#### Radar

Radar systems monitor the shape and position of objects by emitting radio frequency waves and receiving reflections. By monitoring the small movements of the chest, radar systems can detect vital signs such as heart rate [143] and respiration rate [144]. Breathing and location can be used to monitor sleep quality [145]. The radar system can be installed on the wall or above the person being monitored. The systems have been tested in healthy adults.

##### Clinimetrics

Radar systems have demonstrated high accuracy in monitoring respiration rate [144], heart rate [143], sleep time, and sleep efficiency [145] in healthy adults.

##### Feasibility and acceptability

No manuscripts have reported on the feasibility and acceptability of these systems.

#### Multiple sensors

Only a few studies have experimented with the use of multiple noncontact sensors. Sleep or agitation was monitored using (infrared) cameras, acoustic sensors, and pressure mats in healthy adults, people with COVID-19, community-dwelling older adults, and people with dementia [146–148].

##### Clinimetrics

The clinimetrics of systems using multiple noncontact sensors were not yet optimal. Radar and pressure mats overestimated total sleep time and sleep accuracy in the laboratory among community-dwelling older adults [147]. The reliability of the measurements of heart rate and respiration rate of healthy adults and people with dementia differed depending on the setting [146]. Infrared cameras and a pressure mat complimented each other in their registration of movement of patients with COVID-19 [148].

##### Feasibility and acceptability

In the study with people with dementia in the emergency department and a geriatric-psychiatric ward, few disruptions occurred during the measurements, and one out of 19 participants with dementia refused to be monitored [146].

## Discussion

A variety of monitoring technologies are available to monitor discomfort and distressing symptoms that can occur at the end of life, including actigraphy, brain activity monitoring, ECG, EDA monitoring, surface EMG, incontinence sensors, multimodal systems, and noncontact monitoring systems. Although there were no validation studies in the end-of-life setting, the clinimetrics of selected technologies, especially actigraphy and portable polysomnography, have been researched and were satisfactory in other settings. Relatively few manuscripts have reported on the feasibility and acceptability of these technologies, and even fewer have been conducted in the end-of-life setting, except for brain activity monitors, which have been reported to be acceptable for palliative sedation. In the other settings, the technologies were deemed feasible most of the time. Challenges for feasibility included positioning of the device, signal quality during movement, problems with Bluetooth or Wi-Fi connections, battery life, and the device being taken off due to agitation.

This review demonstrated the rapid development of monitoring technologies, and we share the expectations of others that user-friendly systems with small, wireless, and noncontact sensors or smart garments will soon be available as minimally invasive options to monitor a wide range of discomfort and distressing symptoms in end-of-life care [26, 149, 150]. Currently, promising research is being conducted on the monitoring of a variety of indicators of discomfort with technology. Specifically, the depth of sedation may be monitored using brain activity monitors; sleep/wake states using actigraphy, brain activity monitors, portable PSG, infrared sensors, and radar; sleep disordered breathing using portable PSG, actigraphy, pressure mats, and ECG; risk of pressure ulcers using actigraphy; urinary incontinence using incontinence monitors; agitation using actigraphy and multi-modal systems; pain using brain activity monitors, ECG, EDA, multi-modal systems, facial expression analysis; delirium using brain activity monitors, and multimodal systems with environmental sensors; stress using brain activity monitors, EDA, multi-modal systems; fatigue using multi-modal systems; and thermal comfort using infrared sensors. However, we are not yet able to make recommendations for clinical practice due to limited research in this setting. Many studies included in this review contained a small sample, some technologies were only validated by the development team, and previous reviews on specific types of technologies, such as urinary incontinence monitors and BIS, reported very low quality of evidence [151, 152]. The included feasibility studies only focused on technical aspects and employed diverse, often unclear endpoints, which was also the case in a previous review on e-health in geriatric rehabilitation [153].

More attention is needed to examine the clinimetrics, feasibility, and acceptability of the technologies with solid study designs, a sample to generate enough power for the proposed analyses, and standardized outcome measurements. The Health Technology Assessment (HTA) core model may be a useful tool to guide future studies in systematically evaluating technologies from multiple dimensions. It provides a list of relevant research questions, corresponding outcome measurements and possible sources of information [154]. However, this model primarily guides the synthesis of evidence instead of primary research. For the latter, more detailed instructions would be needed. For example, with the note that the cutoffs for good clinimetrics should not be fixed but depend on the goal of the technology [155], more information is needed on how to determine these standards. With the accelerated development in the field of healthcare technology, we recommend better implementation of existing evaluation guidelines and further development of the guidelines to directly guide the design of primary studies.

This review has a few limitations and strengths. Many of the included manuscripts were not about the end-of-life setting, and we did not evaluate the quality of the studies. Commercially available technologies without scientific studies were also not systematically searched for. These were beyond the aim of our scoping review. Since the search terms were in English, manuscripts in other languages without an English title or abstract were not included. Despite these limitations, the authors believe that this review identified most monitoring technologies that were already applied or developed for settings similar to the end of life. We implemented an elaborate search strategy consisting of broad search terms, including manuscripts in multiple languages and gray literature, and conducted an expert consultation to include technologies that are still under development. To the best knowledge of the authors, this is the first review on noninvasive monitoring technologies that may be applied to the end of life, where people are often bed-ridden and have limited communication abilities. Future research is needed to assess the methodological quality of the studies, to identify potentially useful technologies for the end of life, to further develop them and validate them in this setting and to compare different technologies measuring the same symptoms.

## Conclusions

This review provides an overview for clinicians, researchers and technology developers of noninvasive technologies that can potentially be used to monitor discomfort and distressing symptoms in persons with limited communication at the end of life. A variety of technologies are available to monitor symptoms such as sleep, level of consciousness, risk of pressure ulcers, urinary incontinence, agitation, and pain. Clinimetrics and feasibility in non-end-of-life settings were promising, while acceptability studies were scarce. Future research should evaluate the usefulness, acceptability and feasibility of the identified technologies in end-of-life care, further develop the technologies and validate them in this setting. Guidelines for studies on healthcare technologies should also be developed.

### Supplementary Information

**Additional file 1: eTable 1** Manuscripts reporting clinimetrics of the monitoring technologies to detect distress and discomfort.

**Additional file 2: eTable 2** Manuscripts reporting on the acceptability or feasibility of the monitoring technologies to detect distress and discomfort.

**Additional file 3: eTable 3** Manuscripts in which the clinimetrics, acceptability or feasibility of the technologies that monitor distress and discomfort were not reported (the technology is either used as standard measurement or is newly developed).

**Additional file 4. **References of included articles.

**Additional file 5. **Search strategy.

## Data Availability

No datasets were generated or analysed during the current study.

## Abbreviations

ICU: Intensive care unit
BIS: Bispectral Index
AUC: Area under the curve
EEG: Electroencephalography
ECG: Electrocardiography
EDA: Electrodermal activity
sEMG: Surface electromyography
PSG: Polysomnography
rPPG: Remote photoplethysmography
HTA: Health Technology Assessment

## References

[1] Albert RH (2017). End-of-life care: managing common symptoms. Am Fam Physician.

[2] van der Steen JT (2010). Dying with dementia: what we know after more than a decade of research. J Alzheimers Dis.

[3] Fleming J, Calloway R, Perrels A, Farquhar M, Barclay S, Brayne C (2017). Dying comfortably in very old age with or without dementia in different care settings - a representative “older old” population study. BMC Geriatr.

[4] Binda F, Clari M, Nicolo G, Gambazza S, Sappa B, Bosco P (2021). Quality of dying in hospital general wards: a cross-sectional study about the end-of-life care. BMC Palliat Care.

[5] Pivodic L, Smets T, Van den Noortgate N, Onwuteaka-Philipsen BD, Engels Y, Szczerbinska K (2018). Quality of dying and quality of end-of-life care of nursing home residents in six countries: an epidemiological study. Palliat Med.

[6] Hui D, Dev R, Bruera E (2015). The last days of life: symptom burden and impact on nutrition and hydration in cancer patients. Curr Opin Support Palliat Care.

[7] Koppitz A, Bosshard G, Schuster DH, Hediger H, Imhof L (2015). Type and course of symptoms demonstrated in the terminal and dying phases by people with dementia in nursing homes. Z Gerontol Geriatr.

[8] Su A, Lief L, Berlin D, Cooper Z, Ouyang D, Holmes J (2018). Beyond pain: nurses’ assessment of patient suffering, dignity, and dying in the intensive care unit. J Pain Symptom Manage.

[9] Agar M, Bush SH (2020). Delirium at the end of life. Med Clin N Am.

[10] Knoepfel S, Bode L, Gehrke S, Spiller T, Fuchs S, Ernst J (2021). Delirium at the end of life. Palliat Support Care.

[11] Radbruch L, De Lima L, Knaul F, Wenk R, Ali Z, Bhatnaghar S (2020). Redefining palliative care-a new consensus-based definition. J Pain Symptom Manage.

[12] Soto-Rubio AL, Miguel JMT, Perez-Marin M, Martin PB (2019). Patients with limited communication in end-of-life situations: Initial psychometric properties of a discomfort observation scale. J Health Psychol.

[13] O’Connor T, Paterson C, Gibson J, Strickland K. The conscious state of the dying patient: an integrative review. Palliat Support Care. 2021;20(5):731–43. 10.1017/S1478951521001541 34615571

[14] National Consensus Project for Quality Palliative Care (2018). Clinical practice guidelines for quality palliative care.

[15] Bouajram RH, Sebat CM, Love D, Louie EL, Wilson MD, Duby JJ (2020). Comparison of self-reported and behavioral pain assessment tools in critically Ill patients. J Intensive Care Med.

[16] Six S, Laureys S, Poelaert J, Mairesse O, Theuns P, Bilsen J (2021). Neurophysiological assessments during continuous sedation until death put validity of observational assessments into question: a prospective observational study. Pain Ther.

[17] Brant JM, Fink RM, Thompson C, Li YH, Rassouli M, Majima T (2019). Global survey of the roles, satisfaction, and barriers of home health care nurses on the provision of palliative care. J Palliat Med.

[18] Khan SS, Ye B, Taati B, Mihailidis A (2018). Detecting agitation and aggression in people with dementia using sensors-a systematic review. Alzheimers Dementia.

[19] Nair P, Subha V, editors. Facial expression analysis for distress detection. In: 2018 Second International Conference on Electronics, Communication and Aerospace Technology (ICECA). 2018 Mar 29-31; Coimbatore, India. New York City: United States of America: IEEE; 2018. p. 1652–55.

[20] Ledowski T (2019). Objective monitoring of nociception: a review of current commercial solutions. Br J Anaesth.

[21] Barbato M, Barclay G, Potter J, Yeo W, Chung J (2017). Correlation between observational scales of sedation and comfort and Bispectral index scores. J Pain Symptom Manag.

[22] Cherny NI, Radbruch L, Ca BEAP (2009). European Association for Palliative Care (EAPC) recommended framework for the use of sedation in palliative care. Palliat Med.

[23] Dieudonne Rahm N, Morawska G, Pautex S, Elia N (2021). Monitoring nociception and awareness during palliative sedation: a systematic review. Palliat Med.

[24] Moyle W (2019). The promise of technology in the future of dementia care. Nat Rev Neurol.

[25] Patel S, Park H, Bonato P, Chan L, Rodgers M (2012). A review of wearable sensors and systems with application in rehabilitation. J Neuroeng Rehabil.

[26] Wang ZH, Yang ZC, Dong T (2017). A review of wearable technologies for elderly care that can accurately track indoor position, recognize physical activities and monitor vital signs in real time. Sensors.

[27] Husebo BS, Heintz HL, Berge LI, Owoyemi P, Rahman AT, Vahia IV (2019). Sensing technology to monitor behavioral and psychological symptoms and to assess treatment response in people with dementia. A systematic review. Front Pharmacol.

[28] Naslund JA, Marsch LA, McHugo GJ, Bartels SJ (2015). Emerging mHealth and eHealth interventions for serious mental illness: a review of the literature. J Ment Health.

[29] Widberg C, Wiklund B, Klarare A (2020). Patients' experiences of eHealth in palliative care: an integrative review. BMC Palliat Care.

[30] Peters MDJ, Godfrey C, McInerney P, Munn Z, Tricco AC, Khalil H. Chapter 11: Scoping reviews (2020 version). In: Aromataris E MZE, editor. JBI Manual for Evidence Synthesis. JBI. 2020. Available from: https://synthesismanual.jbi.global, 10.46658/JBIMES-20-01.

[31] Munn Z, Peters MDJ, Stern C, Tufanaru C, McArthur A, Aromataris E (2018). Systematic review or scoping review? Guidance for authors when choosing between a systematic or scoping review approach. BMC Med Res Methodol.

[32] Tricco AC, Lillie E, Zarin W, O'Brien KK, Colquhoun H, Levac D (2018). PRISMA Extension for Scoping Reviews (PRISMA-ScR): checklist and explanation. Ann Intern Med.

[33] Xu J, van der Steen JT, Smaling HJA, Achterberg WP (2022). Non-invasive monitoring technologies to identify discomfort and distressing symptoms in persons with limited communication at the end of life: protocol of a scoping review.

[34] Tura A, Maran A, Pacini G (2007). Non-invasive glucose monitoring: assessment of technologies and devices according to quantitative criteria. Diabetes Res Clin Pr.

[35] Mandrekar JN (2010). Receiver operating characteristic curve in diagnostic test assessment. J Thorac Oncol.

[36] Sheldrick RC, Benneyan JC, Kiss IG, Briggs-Gowan MJ, Copeland W, Carter AS (2015). Thresholds and accuracy in screening tools for early detection of psychopathology. J Child Psychol Psychiatry.

[37] Anusha G, Sujatha V, Swarnalatha M, Hema B, Lakshmi DM (2021). An advanced nursing homes activity tracking for elderly care support. J Cardiovasc Dis Res.

[38] Cicceri G, De Vita F, Bruneo D, Merlino G, Puliafito A (2020). A deep learning approach for pressure ulcer prevention using wearable computing. Human-centric Comput Inf Sci.

[39] Chikhaoui B, Ye B, Mihailidis A. Ensemble learning-based algorithms for aggressive and agitated behavior recognition. In: García CR, Caballero-Gil P, Burmester M, Quesada-Arencibia A, editors. Ubiquitous Computing and Ambient Intelligence. 2016 Nov 29-Dec 2, San Bartolomé de Tirajana, Spain. Cham (CH): Springer; 2016. p. 9–20.

[40] Delaney L, Litton E, Melehan K, Huang HC, Lopez V, Van Haren F (2021). The feasibility and reliability of actigraphy to monitor sleep in intensive care patients: an observational study. Crit Care.

[41] Blytt KM, Bjorvatn B, Husebo B, Flo E (2017). Clinically significant discrepancies between sleep problems assessed by standard clinical tools and actigraphy. BMC Geriatr.

[42] Most EI, Aboudan S, Scheltens P, Van Someren EJ (2012). Discrepancy between subjective and objective sleep disturbances in early- and moderate-stage Alzheimer disease. Am J Geriatr Psychiatry.

[43] Maskevich S, Jumabhoy R, Dao PDM, Stout JC, Drummond SPA (2017). Pilot validation of ambulatory activity monitors for sleep measurement in Huntington’s disease gene carriers. J Huntingtons Dis.

[44] Svetnik V, Wang TC, Ceesay P, Snyder E, Ceren O, Bliwise D (2021). Pilot evaluation of a consumer wearable device to assess sleep in a clinical polysomnography trial of suvorexant for treating insomnia in patients with Alzheimer’s disease. J Sleep Res.

[45] Mokhtaran M, Sacchi L, Tibollo V, Risi I, Ramella V, Quaglini S, et al. Obstructive sleep apnea home-monitoring using a commercial wearable device. In: MEDINFO 2021: One World, One Health - Global Partnership for Digital Innovation. Proceedings of the 18th World Congress on Medical and Health Informatics. 2021 Oct 2-4; Online. The Netherlands: Studies in Health Technology and Informatics; 2022. p. 522–5. 10.3233/SHTI220131 35673070

[46] Alessi CA, Yoon EJ, Schnelle JF, Al-Samarrai NR, Cruise PA (1999). A randomized trial of a combined physical activity and environmental intervention in nursing home residents: do sleep and agitation improve?. J Am Geriatr Soc.

[47] Gibson RH, Gander PH (2019). Monitoring the sleep patterns of people with dementia and their family carers in the community. Australas J Ageing.

[48] Mahlberg R, Walther S (2007). Actigraphy in agitated patients with dementia. Monitoring treatment outcomes. Z Gerontol Geriatr.

[49] Knuff A, Leung RH, Seitz DP, Pallaveshi L, Burhan AM (2019). Use of actigraphy to measure symptoms of agitation in dementia. Am J Geriatr Psychiatry.

[50] Nagels G, Engelborghs S, Vloeberghs E, Van Dam D, Pickut BA, De Deyn PP (2006). Actigraphic measurement of agitated behaviour in dementia. Int J Geriatr Psychiatry.

[51] Bankole A, Anderson M, Smith-Jackson T, Knight A, Oh K, Brantley J (2012). Validation of noninvasive body sensor network technology in the detection of agitation in dementia. Am J Alzheimers Dis Other Demen.

[52] Raj R, Ussavarungsi K, Nugent K (2014). Accelerometer-based devices can be used to monitor sedation/agitation in the intensive care unit. J Crit Care.

[53] Pickham D, Berte N, Pihulic M, Valdez A, Mayer B, Desai M (2018). Effect of a wearable patient sensor on care delivery for preventing pressure injuries in acutely ill adults: a pragmatic randomized clinical trial (LS-HAPI study). Int J Nurs Stud.

[54] Kikhia B, Stavropoulos TG, Andreadis S, Karvonen N, Kompatsiaris I, Sävenstedt S (2016). Utilizing a wristband sensor to measure the stress level for people with dementia. Sensors (Basel).

[55] van Dijk E, Hilgenkamp TI, Evenhuis HM, Echteld MA (2012). Exploring the use of actigraphy to investigate sleep problems in older people with intellectual disability. J Intellect Disabil Res.

[56] Leblanc RG, Czarnecki P, Howard J, Jacelon CS, Marquard J (2022). Usability experience of a personal sleep monitoring device to self-manage sleep among persons 65 years or older with self-reported sleep disturbances. Comput Inform Nurs.

[57] Favela J, Cruz-Sandoval D, Morales-Tellez A, Lopez-Nava IH (2020). Monitoring behavioral symptoms of dementia using activity trackers. J Biomed Inform.

[58] Pu L, Lion KM, Todorovic M, Moyle W (2021). Portable EEG monitoring for older adults with dementia and chronic pain - a feasibility study. Geriatr Nurs.

[59] Bass S, Vance ML, Reddy A, Bauer SR, Roach E, Torbic H (2019). Bispectral index for titrating sedation in ARDS patients during neuromuscular blockade. Am J Crit Care.

[60] Arbour RB, Dissin J (2015). Predictive value of the bispectral index for burst suppression on diagnostic electroencephalogram during drug-induced coma. J Neurosci Nurs.

[61] Arbour C, Gélinas C, Loiselle CG, Bourgault P (2015). An exploratory study of the bilateral bispectral index for pain detection in traumatic-brain-injured patients with altered level of consciousness. J Neurosci Nurs.

[62] Giménez S, Romero S, Alonso JF, Mañanas M, Pujol A, Baxarias P (2017). Monitoring sleep depth: analysis of bispectral index (BIS) based on polysomnographic recordings and sleep deprivation. J Clin Monit Comput.

[63] Pedrao RAA, Riella RJ, Richards K, Valderramas SR (2020). Viability and validity of the bispectral index to measure sleep in patients in the intensive care unit. Rev.

[64] Luo A, Muraida S, Pinchotti D, Richardson E, Ye E, Hollingsworth B, et al. Bispectral index monitoring with density spectral array for delirium detection. J Acad Consult Liaison Psychiatry. 2021;62(3):318–29. 10.1016/j.psym.2020.08.008 33223218

[65] Gambrell M (2005). Using the BIS monitor in palliative care: a case study. J Neurosci Nurs.

[66] Roh T, Bong K, Hong S, Cho H, Yoo HJ. Wearable mental-health monitoring platform with independent component analysis and nonlinear chaotic analysis. In: Conference proceedings: 2012; Annual International Conference of the IEEE Engineering in Medicine and Biology Society. 2012 Aug 20-Sep 1; Sandiego, United States of America. New York City (US): IEEE Engineering in Medicine and Biology Society; 2012. p. 4541–4. 10.1109/EMBC.2012.6346977 23366938

[67] Nakamura T, Alqurashi YD, Morrell MJ, Mandic DP (2020). Hearables: automatic overnight sleep monitoring with standardized in-ear EEG sensor. IEEE Trans Biomed Eng.

[68] Espie CA, Paul A, McFie J, Amos P, Hamilton D, McColl JH (1998). Sleep studies of adults with severe or profound mental retardation and epilepsy. Am J Ment Retard.

[69] Sato R, Kanda K, Anan M, Watanuki S (2002). Sleep EEG patterns and fatigue of middle-aged and older female family caregivers providing routine nighttime care for elderly persons at home. Percept Mot Skills.

[70] Six S, Laureys S, Poelaert J, Bilsen J, Theuns P, Musch L (2019). Should we include monitors to improve assessment of awareness and pain in unconscious palliatively sedated patients? A case report. Palliat Med.

[71] Vacas S, McInrue E, Gropper MA, Maze M, Zak R, Lim E (2016). The feasibility and utility of continuous sleep monitoring in critically Ill patients using a portable electroencephalography monitor. Anesth Analg.

[72] Urdanibia-Centelles O, Nielsen RM, Rostrup E, Vedel-Larsen E, Thomsen K, Nikolic M (2021). Automatic continuous EEG signal analysis for diagnosis of delirium in patients with sepsis. Clin Neurophysiol.

[73] Spira AP, Stone KL, Redline S, Ensrud KE, Ancoli-Israel S, Cauley JA (2017). Actigraphic sleep duration and fragmentation in older women: associations with performance across cognitive domains. Sleep.

[74] De jonckheere J, Dassonneville A, Flocteil M, Delecroix M, Seoane G, Jeanne M (2014). Ambulatory pain evaluation based on heart rate variability analysis: application to physical therapy. Annu Int Conf IEEE Eng Med Biol Soc.

[75] Grasso I, Haigney M, Mortara D, Collen JF, Hostler J, Moores A (2018). Detection of sleep-disordered breathing with ambulatory Holter monitoring. Sleep Breath.

[76] Park HJ, Choi D, Park HA, Lee CA (2022). Nurse evaluation of stress levels during CPR training with heart rate variability using smartwatches according to their personality: a prospective, observational study. PLoS One.

[77] Estrada CA, Rosman HS, Prasad NK, Battilana G, Alexander M, Held AC (2000). Evaluation of guidelines for the use of telemetry in the non-intensive-care setting. J Gen Intern Med.

[78] Gerber SM, Jeitziner MM, Knobel SE, Mosimann UP, Müri RM, Jakob SM (2019). Perception and performance on a virtual reality cognitive stimulation for use in the intensive care unit: a non-randomized trial in critically ill patients. Front Med.

[79] Gerber SM, Jeitziner M-M, Sänger SD, Knobel SE, Marchal-Crespo L, Müri RM (2019). Comparing the relaxing effects of different virtual reality environments in the intensive care unit: observational study. JMIR Perioper Med.

[80] Li X, Zhu W, Sui X, Zhang A, Chi L, Lv L (2021). Assessing workplace stress among nurses using heart rate variability analysis with wearable ECG device-a pilot study. Front Public Health.

[81] Tao L, Yi YP, Shan Y, Yu D, Zhang J, Qu YS (2021). Analysis on severe fever with thrombocytopenia syndrome bunyavirus infection combined with atrial fibrillation under digital model detection. Results Phys.

[82] Setz C, Arnrich B, Schumm J, La Marca R, Troster G, Ehlert U (2010). Discriminating stress from cognitive load using a wearable EDA device. IEEE Trans Inform Technol Biomed.

[83] Jussila J, Venho N, Salonius H, Moilanen J, Liukkonen J, Rinnetmäki M, editors. Towards ecosystem for research and development of electrodermal activity applications. In: Proceedings of the 22nd International Academic Mindtrek Conference. 2018 Oct 10-11; Tempere, Finland. New York (US): Association for Computing Machinery; 2018. p. 79–87.

[84] de Vries S, Smits R, Tataj M, Ronckers M, van der Pol M, van Oost F (2022). Accurate stress detection from novel real-time electrodermal activity signals and multi-task learning models. Cognit Comput Internet Things.

[85] Aslanidis T, Grosomanidis V, Karakoulas K, Chatzisotiriou A (2018). Electrodermal activity monitoring during painful stimulation in sedated adult intensive care unit patients: a pilot study. Acta Medica (Hradec Kralove).

[86] Leborgne F, Smits R, Gencheva M, De Vries S, Meinders E, Cluitmans P, et al. The development of a washable and durable smart textile to measure electrodermal activity for early stress recognition. In Ahram T, Karwowski W, Bucchianico PD, Taiar R, Casarotto L, Coasta P, editors. Intelligent Human Systems Integration (IHSI 2023): Integrating People and Intelligent Systems. 2023 Feb 22-24; Venice, Italy. USA: AHFE International; 2023;69:513–23.

[87] Carlson CR, Wynn KT, Edwards J, Okeson JP, Nitz AJ, Workman DE (1996). Ambulatory electromyogram activity in the upper trapezius region: patients with muscle pain vs. pain-free control subjects. Spine.

[88] Yang G, Jiang M, Ouyang W, Ji G, Xie H, Rahmani AM (2018). IoT-based remote pain monitoring system: from device to cloud platform. IEEE J Biomed Health Inform.

[89] Fischer M, Renzler M, Ussmueller T (2019). Development of a smart bed insert for detection of incontinence and occupation in elder care. IEEE Access.

[90] van der Hurk PR, Middelkoop HA, van Waalwijk-van Doorn ES, Roos RA, Cools HJ (1998). Long-term ambulatory monitoring of urine leakage in the elderly: an evaluation of the validity and clinical applicability of thermistor signalling. J Med Eng Technol.

[91] Nikoletti S, Young J, King M (2004). Evaluation of an electronic monitoring device for urinary incontinence in elderly patients in an acute care setting. J Wound Ostomy Continence Nurs.

[92] Tekcin M, Sayar E, Yalcin MK, Bahadir SK (2022). Wearable and flexible humidity sensor integrated to disposable diapers for wetness monitoring and urinary incontinence. Electronics.

[93] AB EHaH. [TENA Identifi TM is bewezen effectief] TENA Identifi TM is proved effective. 2023. Available from: https://tena-images.essity.com/images-c5/789/261789/original/essitynl9049-def-identifi-factsheet-nw.pdf.

[94] Boerakker R (2022). Project Eind Rapport (PER) - pilot slim incontinentiemateriaal.

[95] To KW, Chan TO, Chan WC, Choo KL, Hui DSC. Using a portable monitoring device for diagnosing obstructive sleep apnea in patients with multiple coexisting medical illnesses. Clin Respir J. 2021;15(10):1104–12. 10.1111/crj.13416 34224640

[96] Chang Y, Xu L, Han F, Keenan BT, Kneeland-Szanto E, Zhang R (2019). Validation of the Nox-T3 portable monitor for diagnosis of obstructive sleep apnea in patients with chronic obstructive pulmonary disease. J Clin Sleep Med.

[97] Polese JF, Santos-Silva R, de Oliveira Ferrari PM, Sartori DE, Tufik S, Bittencourt L (2013). Is portable monitoring for diagnosing obstructive sleep apnea syndrome suitable in elderly population?. Sleep Breath.

[98] Jungquist CR, Chandola V, Spulecki C, Nguyen KV, Crescenzi P, Tekeste D (2019). Identifying patients experiencing opioid-induced respiratory depression during recovery from anesthesia: the application of electronic monitoring devices. Worldviews Evid Based Nurs.

[99] Arnal PJ, Thorey V, Debellemaniere E, Ballard ME, Bou Hernandez A, Guillot A (2020). The Dreem Headband compared to polysomnography for electroencephalographic signal acquisition and sleep staging. Sleep.

[100] Kang D, Ye JY, Zheng L, Zhang JB, Bian QL (2012). Evaluation of Watch PAT as a diagnosing test for patients with obstructive sleep apnea hypopnea syndrome. Zhonghua Er Bi Yan Hou Tou Jing Wai Ke Za Zhi.

[101] Mäkinen N, Huuskonen U, Hannila E, Pisilä A-P, Alaniemi L, Koskinen K (2022). System validation study for novel wearable sleep apnea screening device. Sleep Med.

[102] Ghorbani S, Golkashani HA, Chee N, Teo TB, Dicom AR, Yilmaz G (2022). Multi-night at-home evaluation of improved sleep detection and classification with a memory-enhanced consumer sleep tracker. Nat Sci Sleep.

[103] Li S, Xu L, Dong X, Zhang X, Keenan BT, Han F (2021). Home sleep apnea testing of adults with chronic heart failure. J Clin Sleep Med.

[104] Tedeschi E, Carratu P, Damiani MF, Ventura VA, Drigo R, Enzo E (2013). Home unattended portable monitoring and automatic CPAP titration in patients with high risk for moderate to severe obstructive sleep apnea. Respir Care.

[105] Supe D, Baron L, Decker T, Parker K, Venella J, Williams S (2017). Research: continuous surveillance of sleep apnea patients in a medical-surgical unit. Biomed Instrum Technol.

[106] Buyse B, Borzée P, Kalkanis A, Testelmans D (2023). In search of a cut-off apnea-hypopnea index in type 3 home portable monitors to diagnose and treat obstructive sleep apnea: a mathematical simulation. J Sleep Res.

[107] Abdenbi F, Ahnaou A, Royant-Parola S, Nedelcoux H, Rouault S, Alfandary D (2002). Ambulatory sleep recording in a healthcare network: a feasibility study. Comptes Rendus Biol.

[108] Morales CR, Hurley S, Wick LC, Staley B, Pack FM, Gooneratne NS (2012). In-home, self-assembled sleep studies are useful in diagnosing sleep apnea in the elderly. Sleep.

[109] Koskinen K, Hannila E, Kallio M, Huuskonen U, Himanen SL. Clinical study: evaluating the usability and clinical performance of the Nukute collare system. Oulu; 2021. https://nukute.com/products/clinical-validation.

[110] Sylvia LG, Salcedo S, Bianchi MT, Urdahl AK, Nierenberg AA, Deckersbach T (2014). A novel home sleep monitoring device and brief sleep intervention for bipolar disorder: feasibility, tolerability, and preliminary effectiveness. Cogn Ther Res.

[111] Lazazzera R, Carrault G. MonEco: a novel health monitoring ecosystem to predict respiratory and cardiovascular disorders. Irbm. 2023;44(2):100736.

[112] Au-Yeung WTM, Miller L, Beattie Z, Dodge HH, Reynolds C, Vahia I (2020). Sensing a problem: proof of concept for characterizing and predicting agitation. Alzheimers Dement Transl Res Clin Interv.

[113] Bankole A, Anderson MS, Homdee N, Alam R, Lofton A, Fyffe N (2020). BESI: Behavioral and Environmental Sensing and Intervention for dementia caregiver empowerment-phases 1 and 2. Am J Alzheimers Dis Other Demen.

[114] Khan SS, Spasojevic S, Nogas J, Ye B, Mihailidis A, Iaboni A (2019). Agitation detection in people living with dementia using multimodal sensors. Annu Int Conf IEEE Eng Med Biol Soc.

[115] Davoudi A, Malhotra KR, Shickel B, Siegel S, Williams S, Ruppert M (2019). Intelligent ICU for autonomous patient monitoring using pervasive sensing and deep learning. Sci Rep.

[116] Hoehn-Saric R, McLeod DR, Funderburk F, Kowalski P (2004). Somatic symptoms and physiologic responses in generalized anxiety disorder and panic disorder: An ambulatory monitor study. Arch Gen Psychiatry.

[117] Rose K, Specht J, Forch W (2015). Correlates among nocturnal agitation, sleep, and urinary incontinence in dementia. Am J Alzheimers Dis Other Demen.

[118] Davidoff H, van den Bulcke L, Vandenbulcke M, De Vos M, van den Stock J, Van Helleputte N (2022). Toward quantification of agitation in people with dementia using multimodal sensing. Innov Aging.

[119] Jiang M, Mieronkoski R, Syrjälä E, Anzanpour A, Terävä V, Rahmani AM (2019). Acute pain intensity monitoring with the classification of multiple physiological parameters. J Clin Monit Comput.

[120] Choi J, Ahmed B, Gutierrez-Osuna R (2012). Development and evaluation of an ambulatory stress monitor based on wearable sensors. IEEE Trans Inform Technol Biomed.

[121] Miranda D, Favela J, Ibarra C, Cruz N (2016). Naturalistic enactment to elicit and recognize caregiver state anxiety. J Med Syst.

[122] Rajasekaran S, Luteran C, Qu H, Riley-Doucet C (2011). A portable autonomous multisensory intervention device (PAMID) for early detection of anxiety and agitation in patients with cognitive impairments. Annu Int Conf IEEE Eng Med Biol Soc.

[123] Lai Kwan C, Mahdid Y, Motta Ochoa R, Lee K, Park M, Blain-Moraes S. Wearable technology for detecting significant moments in individuals with dementia. Biomed Res Int. 2019;10:1–13. 10.1155/2019/6515813 PMC6778872 31662986

[124] Iaboni A, Spasojevic S, Newman K, Schindel Martin L, Wang A, Ye B (2022). Wearable multimodal sensors for the detection of behavioral and psychological symptoms of dementia using personalized machine learning models. Alzheimers Dement.

[125] Hassan SR, Ahmad I, Ahmad S, Alfaify A, Shafiq M (2020). Remote pain monitoring using fog computing for e-healthcare: an efficient architecture. Sensors.

[126] Wijsman J, Grundlehner B, Liu H, Hermens H, Penders J. Towards mental stress detection using wearable physiological sensors. In: Conference proceedings: Annual International Conference of the IEEE Engineering in Medicine and Biology Society. 2011 Aug 30-Sep 3; Boston, USA. New York (USA): IEEE Engineering in Medicine and Biology Society; 2011. p. 1798–801. 10.1109/IEMBS.2011.6090512 22254677

[127] Wu W, Gil Y, Lee J (2012). Combination of wearable multi-biosensor platform and resonance frequency training for stress management of the unemployed population. Sensors (Switzerland).

[128] Gelinas C, Shahiri TS, Richard-Lalonde M, Laporta D, Morin JF, Boitor M (2021). Exploration of a multi-parameter technology for pain assessment in postoperative patients after cardiac surgery in the intensive care unit: the Nociception Level Index (NOL)TM. J Pain Res.

[129] Spasojevic S, Nogas J, Iaboni A, Ye B, Mihailidis A, Wang A (2021). A pilot study to detect agitation in people living with dementia using multi-modal sensors. J Healthc Inform Res.

[130] Ramirez-Moreno MA, Carrillo-Tijerina P, Candela-Leal MO, Alanis-Espinosa M, Tudon-Martinez JC, Roman-Flores A (2021). Evaluation of a fast test based on biometric signals to assess mental fatigue at the workplace-a pilot study. Int J Environ Res Public Health.

[131] Dietz-Terjung S, Geldmacher J, Brato S, Linker CM, Welsner M, Schobel C, et al. A novel minimal-contact biomotion method for long-term respiratory rate monitoring. Sleep Breath. 2021;25:145–9. 10.1007/s11325-020-02067-4 32297144

[132] Peterson MJ, Gravenstein N, Schwab WK, van Oostrom JH, Caruso LJ (2013). Patient repositioning and pressure ulcer risk–monitoring interface pressures of at-risk patients. J Rehabil Res Dev.

[133] Tong Y, Zhang Q, Cheng C, She C, Song W, Cui S (2015). Analysis of monitoring results of mattress-type of sleep monitoring system in elderly patients with OSAHS. Lin Chung Er Bi Yan Hou Tou Jing Wai Ke Za Zhi.

[134] Higami Y, Yamakawa M, Shigenobu K, Kamide K, Makimoto K (2019). High frequency of getting out of bed in patients with Alzheimer's disease monitored by non-wearable actigraphy. Geriatr Gerontol Int.

[135] Kobayashi M, Namba K, Tsuiki S, Nakamura M, Hayashi M, Mieno Y (2013). Validity of sheet-type portable monitoring device for screening obstructive sleep apnea syndrome. Sleep Breath.

[136] Sakai K, Sanada H, Matsui N, Nakagami G, Sugama J, Komiyama C (2009). Continuous monitoring of interface pressure distribution in intensive care patients for pressure ulcer prevention. J Adv Nurs.

[137] Castillo LI, Browne ME, Hadjistavropoulos T, Prkachin KM, Goubran R (2020). Automated vs. manual pain coding and heart rate estimations based on videos of older adults with and without dementia. J Rehabil Assist Technol Eng.

[138] Becouze P, Hann CE, Chase JG, Shaw GM (2007). Measuring facial grimacing for quantifying patient agitation in critical care. Comput Methods Programs Biomed.

[139] Palestra G, Pino O (2020). Detecting emotions during a memory training assisted by a social robot for individuals with Mild Cognitive Impairment (MCI). Multimed Tools Appl.

[140] Rezaei S, Moturu A, Zhao S, Prkachin KM, Hadjistavropoulos T, Taati B. Unobtrusive pain monitoring in older adults with dementia using pairwise and contrastive training. IEEE J Biomed Health Inform. 2021;25(5):1450–62. 10.1109/JBHI.2020.3045743 33338024

[141] Tejedor B, Casals M, Gangolells M, Macarulla M, Forcada N (2020). Human comfort modelling for elderly people by infrared thermography: evaluating the thermoregulation system responses in an indoor environment during winter. Build Environ.

[142] Casaccia S, Braccili E, Scalise L, Revel GM (2019). Experimental assessment of sleep-related parameters by passive infrared sensors: measurement setup, feature extraction, and uncertainty analysis. Sensors.

[143] Schellenberger S, Shi KL, Steigleder T, Malessa A, Michler F, Hameyer L (2020). A dataset of clinically recorded radar vital signs with synchronised reference sensor signals. Sci Data.

[144] Resuli N, Skubic M, Myungki J. Noninvasive respiration monitoring of different sleeping postures using an rf sensor. In: 2021 IEEE International Conference on Bioinformatics and Biomedicine (BIBM). 2021 Dec 9-12; Online. New York Ciity (USA): IEEE; 2021. p. 1485–90.

[145] Hsu C-Y, Ahuja A, Yue S, Hristov R, Kabelac Z, Katabi D (2017). Zero-effort in-home sleep and insomnia monitoring using radio signals. Proc ACM Interact Mob Wearable Ubiquitous Technol.

[146] Kroll L, Bohning N, Mussigbrodt H, Stahl M, Halkin P, Liehr B (2020). Non-contact monitoring of agitation and use of a sheltering device in patients with dementia in emergency departments: a feasibility study. BMC Psychiatry.

[147] Ravindran KKG, Monica CD, Atzori G, Enshaeifar S, Mahvash-Mohammadi S, Dijk DJ (2021). Validation of technology to monitor sleep and bed occupancy in older men and women. Alzheimers Dement.

[148] Dimitrievski A, Zdravevski E, Lameski P, Villasana MV, Pires IM, Garcia NM (2021). Towards detecting pneumonia progression in COVID-19 patients by monitoring sleep disturbance using data streams of non-invasive sensor networks. Sensors.

[149] Stavropoulos TG, Papastergiou A, Mpaltadoros L, Nikolopoulos S, Kompatsiaris I (2020). IoT wearable sensors and devices in elderly care: a literature review. Sensors (Basel).

[150] Walid B, Ma J, Ma M, Qi A, Luo Y, Qi Y. Recent advances in radar-based sleep monitoring—a review. In: 2021 IEEE Intl Conf on Dependable, Autonomic and Secure Computing, Intl Conf on Pervasive Intelligence and Computing, Intl Conf on Cloud and Big Data Computing, Intl Conf on Cyber Science and Technology Congress (DASC/PiCom/CBDCom/CyberSciTech). 2021 Oct 25-28; Calgary, Canada. New York City (USA): IEEE; 2021. p. 759–66.

[151] Ontario HQ (2018). Electronic monitoring systems to assess urinary incontinence: a health technology assessment. Ont Health Technol Assess Ser.

[152] Shetty RM, Bellini A, Wijayatilake DS, Hamilton MA, Jain R, Karanth S (2018). BIS monitoring versus clinical assessment for sedation in mechanically ventilated adults in the intensive care unit and its impact on clinical outcomes and resource utilization. Cochrane Database Syst Rev.

[153] Kraaijkamp JJM, van Dam van Isselt EF, Persoon A, Versluis A, Chavannes NH, Achterberg WP (2021). eHealth in geriatric rehabilitation: systematic review of effectiveness, feasibility, and usability. J Med Internet Res.

[154] Kristensen FB, Lampe K, Wild C, Cerbo M, Goettsch W, Becla L (2017). The HTA core model (R)-10 years of developing an international framework to share multidimensional value assessment. Value Health.

[155] Joint Action 2 WP (2016). HTA Core Model® version 3.0.

