# Supplementary material for: Noninvasive monitoring technologies to identify discomfort and distressing symptoms in persons with limited communication at the end of life: a scoping review
Source: BMC Palliat Care. 2024 Mar 21;23:78. doi: 10.1186/s12904-024-01371-0 (PMC10956214; doi:10.1186/s12904-024-01371-0)
Supplement: Supplementary file 1 — Additional file 1: eTable 1 Manuscripts reporting clinimetrics of the monitoring technologies to detect distress and discomfort. [file 12904_2024_1371_MOESM1_ESM.docx]

**Supplement I of “Noninvasive monitoring technologies to identify discomfort and distressing symptoms in persons with limited communication at the end of life: A scoping review”**

| eTable 1  *Manuscripts reporting clinimetrics of the monitoring technologies to detect distress and discomfort* | | | | | | | | |
| --- | --- | --- | --- | --- | --- | --- | --- | --- |
| First author | Year | Country | | Monitoring technology (model and brand) | Symptom monitored | Aim of study | Methods: Study design; Participants (age, disease, setting); Procedures | Clinimetrics of the technology (validity, sensitivity, reliability, specificity, responsiveness) |
| **Actigraphy** | | | | | | | | |
| Alam | 2019 | USA | | Pebble watch (Pebble Technology, Corp., Taiwan), Pixie app | Agitation | To predict agitation of people with dementia using multiple-instance learning models. | Quantitative cross-sectional study with 10 older adults with mild to severe dementia living at home. Participants wore the Pebble watch for 30 days. Agitation events were marked by family caregivers. | Accuracy: 76% - 87%. F-score: 0.69-0.73. Area under the receiver-operating characteristic curves (AUC): 0.78-0.92, indicating significantly higher true positive rates compared with single instance learning models. |
| Bankole | 2012 | USA | | TEMPO 3.1 (developed by University of Virginia) | Agitation | To validate the Body Sensor Network (BSN) in measuring agitation. | Quantitative cross-sectional study with 6 older adults with dementia in nursing homes. Each participant wore the devices in the morning, afternoon and evening for 3 hours per session. The Cohen-Mansfield Agitation Inventory (CMAI), the Aggressive Behavior Scale (ABS) and the Mini-Mental State Examination (MMSE) were assessed at each session. | Convergent validity (BSN vs. CMAI and ABS): acceptable for morning sessions, less for afternoon and evening sessions.  Discriminant validity (BSN vs. MMSE): strong.  Secondary validation (Teager scores calculated from the BSN measurements during agitation vs. pre- and post-agitation): strong support |
| Chikhaoui | 2016 | Canada | | Shimmer3 GSR (Shimmer, Dublin, Ireland) | Agitation and aggression | To compare Microsoft Kinect sensor and actigraphy in detecting aggressive and agitation behaviors, and to develop and validate corresponding algorisms. | Quantitative cross-sectional study with 10 adults. They performed aggressive and agitated behaviors wearing the device. All actions were performed 5 times.* | The algorism developed by the researchers using actigraphic data could recognize aggressive and agitated behaviors (F-Measure = 0.85 – 0.94) |
| Grap | 2011 | USA | | Basic Motionlogger (Ambulatory Monitoring Inc, Ardsley, NY) | Agitation | To differentiate behavioral states common in the critically ill population. | Quantitative cross-sectional study with 30 adult healthy volunteers in the laboratory. Participants wore the device on the wrist and ankle and simulated states of calmness, restlessness, and agitation for 10 minutes. | The average movement is significantly different among the three states and between wrist and ankle measurements. |
| Knuff | 2019 | USA | | wGT3x+ activity monitors (Actigraph,  LLC, Pensacola, FL) | Agitation | To evaluate the feasibility and validity of actigraphy as a measurement of agitation in dementia. | Quantitative cross-sectional study. 20 older adults with dementia living in long-term care facilities wore the wristband continuously for a minimum of 24 hours and maximum of 7 days. | Significant difference in motor activity between the low- and high-agitation groups. Most actigraphic data, except for night time measurements, significantly correlated with the CMAI (*r* = 0.74 - 0.77, *p* < 0.001) and the Neuropsychiatric Inventory (NPI, *r* = 0.47 - 0.56, *p* = 0.01 – 0.04) |
| Mahlberg | 2007 | Germany | | Actiwatch (Cambridge Neurotechnology Ltd, Cambridge, UK) | Agitation | To evaluate the actigraphy in measuring day-night rhythm disturbances and agitated behavior. | Randomized controlled trial with 24 older adults with dementia and agitated behavior in a geriatric psychiatry unit. 14 received medication, 10 received placebo. Control group: 10 young and 10 older healthy subjects. The device was worn for at least two weeks. The NPI was scored before and after the treatment period. | The actigraphic agitation is significantly lower in the treatment group compared to placebo and healthy control groups. No correlation between actigraphy and NPI. |
| Nagels | 2006 | Belgium | | Basic motionlogger (Ambulatory monitoring, New York, USA) | Agitation | To assess the association between the actigraphic measure of agitation and a validated scale. | Quantitative cross-sectional study with 110 older adults recently diagnosed with dementia. The device was worn for 48 hours. CMAI was rated by nurses during staff briefings. * | Significant association between actigraphic measures and CMAI (*F* = 126.75, *p* < 0.001). |
| Raj | 2014 | USA | | Actiwatch 2 (Phillips Respironics, Andover, MA) | Agitation/ sedation | To determine the usefulness of actigraphy to measure sedation/agitation in the ICU. | Quantitative cross-sectional study with 86 adults in the ICU of a tertiary care center. Actigraphy was worn for 60 or 70 minutes. The Richmond Agitation Sedation Scale (RASS) was scored every 10 minutes. | Actigraphic data associated with sedation/agitation level as assessed by RASS. |
| Valembois | 2015 | France | | Vivago (Vivago Oy, Espoo, Finland) | Aberrant motor behavior | To evaluate the usefulness of actigraphy to measure disorders in motor behavior. | Quantitative cross-sectional study with 183 older adults, of which 126 had dementia admitted to an intermediate care unit in the hospital. The device was worn for 10 days. Apathy, aberrant motor behavior, agitation and anxiety were evaluated with the NPI. | Motor activity measured by the actigraphy was lower in patients with apathy (*p* = 0.008 - 0.05, depending on time of the day), and was greater in those with aberrant motor behavior (*p* = 0.003 - 0.04 depending on time of the day). No correlation with agitation and anxiety was observed. |
| Anusha | 2021 | India | | Not reported | Risk of pressure ulcers and agitation | To validate the use of accelerometer on the mattress to monitor movement on the bed. | Quantitative non-randomized experimental study in the laboratory. The number of participants was not reported. Accelerometers were placed on the mattress and the participants performed several actions on it, including sitting up, rolling over, and slightly moving the limbs. | The algorithm categorized the activity with a 100% accuracy for a single participant. 92% accuracy when the system was trained with the accelerometer on more locations on the bed. When the system was trained with seven different beds, the accuracy on an eighth bed was 82%. |
| Cicceri | 2020 | Italy | | developed by the researchers | Risk of pressure ulcers | To design and implement a wearable sensor system to prevent pressure ulcers with deep learning. | Quantitative cross-sectional study. 6 adult patients wore a hospital gown with integrated sensors in the medicine department. The body position was monitored. The proposed deep learning method was compared with two machine learning models. | The precision was between 99% and 100%.  The sensitivity was between 93% and 100%. |
| Minteer | 2020 | USA | | PUMP1 and PUMP2, developed by the authors | Risk of pressure ulcers | To test the accuracy of PUMP1 (garment sensors) and PUMP2 (bed sensors) in detecting body repositioning. | Quantitative cross-sectional study with 10 immobile patients in the hospital. They were monitored by both devices for 10 ± 2 hours. The data was compared to body repositioning events identified from a video footage. | Both PUMP1 and PUMP2 captured repositioning movement with 85% reliability.  False positives: 2 events for PUMP1, 1 for PUMP2.  False negatives: 11 events for PUMP1, 7 for PUMP2. |
| Pickham | 2018 | USA | | Leaf Patient Monitoring System (Leaf Healthcare, Inc., San Francisco, CA) | Risk of pressure ulcers | To assess the effectiveness of Leaf on preventing pressure ulcers. | Randomized controlled trial. Among 1,312 patients in ICU, 659 wore a Leaf monitor which helps providers know whether the patient has turned recently. | Patients with Leaf had significantly less chance of pressure ulcers (OR=0.33, 95% CI [0.12, 0.90]). The staff complied to the turning scheme of these patients more (67% vs. control=51%, *p* < 0.005). |
| Alessi | 1999 | USA | | Wrist activity monitor (Augmentech Inc., Pittsburgh, PA) | Sleep | To evaluate the effect of daytime physical activity and night time environment on sleep and agitation. | Quantitative cross-sectional study with 29 older adults with incontinence living in nursing homes. The monitor was worn on the wrist for 5 nights during the night. | Wrist activity monitors had a sensitivity of 95.5% and a specificity of 82.0% monitoring sleep-wake status, compared to behavioral observations. |
| Brooks | 1993 | USA | | Actigraph (Ambulatory Monitoring, Inc., Ardsley, NY) | Sleep | To evaluate the sensitivity of the actigraph to detect insomnia treatment effects. | Quantitative cross-sectional study with 9 older adults with insomnia, without dementia. They wore the actigraph for one night at home. | Change in total sleep time measured by actigraphy correlates with the sleep log (*r* = 0.69, *p* = 0.04). No correlations between the two measurements in changes of time to sleep onset, wake after sleep onset and sleep efficiency. |
| Delaney | 2021 | Australia | | Actiwatch Spectrum Plus (Phillips Respironics Inc, Murrysville, PA) | Sleep | To evaluate the feasibility and reliability of actigraphy to monitor sleep compared with polysomnography (PSG). | Quantitative cross-sectional study. 80 adult patents in the ICU were monitored for 24 hours. | Agreement in identifying sleep and wake states compared with PSG (69.4%; K = 0.386, *p* < 0.05), with a moderate level of sensitivity (65.5%) and specificity (76.1%). Amongst non-ventilated patients: specificity 83.7%; sensitivity 56.7%. Moderate correlation with PSG reported total sleep time (*r* = 0.359, *p* < 0.05, underestimated) and wakefulness (*r* = 0.371, *p* < 0.05, overestimated). |
| van Someren | 2007 | Netherlands | | Actiwatch and Sleep 5 software (Cambridge Neurotechnology, Cambridge, UK) | Sleep | To investigate the reliability of actigraphy sleep monitoring depending on the recording duration. | Quantitative cross-sectional study with 10 adults with insomnia living at home and 12 older adults with dementia at group-care facilities. Participants wore the device continuously for 20 days. | Reliability: 59% - 97%, increasing with the recording duration. The authors recommended at least 7 days of monitoring for reliable data. |
| Gibson | 2019 | New Zealand | | Actiwatch-2. Software: Actiware 2015, Version 6 (Mini Mitter, Respironics) | Sleep | To evaluate the reliability of actigraphy to score sleep patterns. | Quantitative cross-sectional study with 15 older adults with dementia and their family caregivers. Both groups wore the device at home for one week and used an event marker to register relevant events such as bedtime. | People with dementia:  Sleep: sensitivity: 87%, specificity: 80%  Wake: sensitivity 77%, specificity: 61%  Family caregiver:  Sleep: sensitivity: 90%, specificity: 93%  Wake: sensitivity 92%, specificity: 57% |
| Maskevich | 2017 | Australia | | Actiwatch Spectrum Pro (Philips Respironics, Murrysville, PA), Jawbone UP2 and Fitbit One | Sleep | To validate the monitoring devices for sleep measurement. | Quantitative cross-sectional study with 7 adult Huntington’s gene carriers. They wore the devices overnight while undergoing a PSG at the laboratory. | All the actigraphy devices are less accurate than PSG and overestimate sleep time by more than an hour and sleep efficiency by 15%. The researchers concluded that they are sufficient to estimate general sleep-wake patterns. |
| Stavitsky | 2010 | USA | | Actiwatch AW-64 (Mini Mitter, Sunriver, Orlando) | Sleep | To compare self-report with actigraphic data on sleep quality | Quantitative cross-sectional study with 30 community-dwelling adults with Parkinson’s Disease and 14 normal control participants. The device was worn 24 hours a day for 7 days at home. Sleep diaries were kept for the same period. | Actigraphic data of sleep quality correlated with self-report in the Parkinson group but not in the control group. |
| McClure | 2020 | USA | | BioStamp nPoint (MC10, Inc., Lexington, MA, USA) | Sleep – disordered breathing | To develop an analysis system to detect breathing patterns. | Quantitative non-randomized experimental study with 100 healthy adults in the laboratory. Participants simulated central sleep apnea, coughing, obstructive sleep apnea, sighing, and yawning, while being monitored with wireless sensors on the chest and abdomen. | F1 score was 92% for normal breathing, 87% for central sleep apnea, 72% for coughing, 51% for obstructive sleep apnea, 57% for sighing, and 63% for yawning. |
| Mehra | 2008 | USA | | Sleepwatch-O (Ambulatory Monitoring, Inc., Ardsley, NY) | Sleep | To examine the association between actigraphic measures and PSG-determined sleep-disordered breathing (SDB) and/or periodic limb movement disorder (PLMD). | Quantitative cross-sectional study with 455 older women living in the community. The device was worn for three 24-hour periods. The PSG was administered for one night at their home by staff. | Poor sleep efficiency measured by wrist actigraphy was associated with SDB (OR = 2.43, 95% CI: 1.43-4.14) and PLMD (OR = 2.36, 95% CI: 1.34-4.15). Reduced sleep duration was also associated with SDB (OR = 3.18, 95% CI: 1.51-6.68) and PLMD (OR = 3.77, 95% CI: 1.78-7.95). |
| Mokhtaran | 2022 | Italy | | Fitbit Charge 3 (Fitbit Inc., San Francisco, California) | Sleep | To explore the use of Fitbit to monitor sleep quality. | Quantitative cross-sectional study with 26 adults with obstructive sleep apnea. Participants were observed by standard PSG and Fitbit simultaneously for three nights in the hospital (baseline, before treatment, after treatment). | Fitbit correlated with PSG in the estimation of total sleep time (*r* = 0.8-0.9, *p* < 0.001), sleep efficiency (*r* = 0.8-0.9, *p* < 0.001), the percentage of four sleep stages (*r* = 0.5-0.9, *p* = 0.001-0.03).  Sensitivity: 90%-93%  Specificity: 56%-59%  Accuracy: 59%-61%  The measurement of Fitbit improved after the treatment for obstructive sleep apnea. The authors concluded that it is less able to measure abnormal sleep patterns. |
| Svetnik | 2021 | USA | Garmin Vivosmart | | Sleep | To investigate the usefulness of a wearable in measuring treatment effects of insomnia in people with dementia. | Randomized experimental study with 285 older adults with Alzheimer’s disease and insomnia. During insomnia treatment of four weeks, participants were instructed to wear the device all the nights at home. Three overnight measurements with standard PSG were performed in the laboratory at screening, baseline, and at the end of the treatment. | Compared to PSG, the device overestimated total sleep time (e.g., placebo baseline = 412 min for watch and 265 min for PSG) and underestimated the change in total sleep time before and after treatment. |
| Kikhia | 2016 | Sweden,Greece,Netherlands | | Philips sensor DTI-2 (Philips Research, Eindhoven, The Netherlands) | Stress | To validate the sensor system in recognizing stress. | Quantitative cross-sectional study with 6 older adults living in nursing homes. Participants wore the device when they are awake for 2 months. Observation notes were made by the staff. | With sampling rate of an hour and stress level threshold of 3: precision: 19%, recall: 48%, accuracy: 76% |
| **Brain activity monitors - Bispectural Index (BIS)** | | | | | | | | |
| Arbour | 2015 | Canada | | BIS VISTA bilateral BIS (Aspect Medical Systems, Newton, MA) | Pain | To validate BIS in pain detection of people with traumatic brain injury. | Quantitative cross-sectional study with 25 critically ill adults in the ICU with acute traumatic brain injury and altered level of consciousness. They were monitored 1 min before, during, and 15 min after turning and non-invasive blood pressure measurements. Pain behaviors were observed. | BIS measurements increased during turning (nociceptive) but not during non-invasive blood pressure measurements (non-nociceptive). Only BIS of the right hemisphere correlates with pain behavior and only for people with left-sided traumatic brain injury (*r* = 0.99, *p* < 0.001). |
| Arbour | 2015 | USA | | BIS A-2000 XP (Aspect Medical Systems, Inc. Norwood, MA) | Sedation | To evaluate the predictive value of BIS in determining level of burst suppression during drug-induced coma, compared with EEG. | Quantitative prospective, observational cohort study with 4 adults in the ICU under drug-induced coma. 33 hours of monitoring was performed in total. | BIS value, BIS suppression ratio, and BIS burst counts had medium to strong correlations with EEG burst counts. |
| Dahaba | 2011 | Australia, China | | BIS-Vista version 1.4 (Aspect Medical Systems, Newton, MA) | Sleep | To assess whether BIS can quantify sleep depth. | Quantitative cross-sectional study with 10 healthy sleep-deprived adults. Participants were monitored during one sleep.* | BIS value showed a temporal decline corresponding to sleep stages. The lowest score was about 40. |
| Giménez | 2017 | Spain | | BIS A-2000 XP Monitor (Aspect Medical Systems, Inc. Norwood, MA) with Quatro Sensors | Sleep | To assess the feasibility and reliability of BIS for sleep monitoring. | Quantitative cross-sectional study with 12 healthy adults. Sleep was measured with PSG and BIS both at baseline and after sleep deprivation at the laboratory. | BIS value was highly correlated with the hypnogram and could discriminate between deep and light sleep. |
| Pedrao | 2020 | Brazil | | BIS™ Vista Monitoring System (Covidien LLC, Mansfield, United States) | Sleep | To test the feasibility of using BIS to evaluate sleep in critically ill patients. | Quantitative cross-sectional study with 29 critically ill patients on ICU. BIS monitoring was performed for a whole night (12 hours). The participants filled in the Richards-Campbell Sleep Questionnaire (RCSQ) the next morning. | Total sleep volume, total sleep time, and continuous sleep volume measured by BIS were weakly correlated with the RCSQ depth of sleep domain score, overall sleep quality domain score, and total score. Total volume, total time, and continuous volume were moderately correlated with the occurrence of awakenings domain score of RCSQ. |
| Luo | 2021 | USA | | BIS-VISTA with density spectral array (Aspect Medical Systems, Inc. Norwood, MA) | Delirium | To evaluate the use of BIS to identify delirium. | Quantitative cross-sectional study with 40 adults with delirium and 83 adults without delirium. Participants were monitored for 20 minutes at the hospital that they were recruited from. | BIS with density spectral array was similar to the 3-minute Diagnostic Interview for Confusion Assessment Method in identifying delirium. Visual inspection of the density spectral array was not associated with delirium. |
| **Brain activity monitors – other electroencephalography (EEG)-based technologies** | | | | | | | | |
| Haenggi | 2004 | Switzerland | | EMMA (Department of Clinical Neurophysiology, Kuopio University Hospital, Kuopio, Finland) | Sedation | To explore the use of long latency evoked potential (EP) in measuring depth of sedation. | Quantitative cross-sectional study with 10 healthy adults under sedation. EP, which is the brain activity 100ms after a standard auditory stimuli, was measured in different stages of sedation based on the Ramsay Sedation Score. | The brain activity 100ms after a standard auditory stimuli (EP) decreased as the depth of sedation increased. |
| Lee | 2022 | South Korea | | Amp GS5001 (SOSO H&C, Kyungpook University, Daegu, Korea) | Delirium | To test a compact EEG device as diagnostic tool for delirium by analysing differences in EEG signals in patients with and without delirium both before and after spinal surgery. | Quantitative cross-sectional study with 37 older adults who had spinal surgery. EEG measurements were performed before, one week after and three months after the surgery in the hospital or outpatient clinic. Delirium was diagnosed by a psychiatrist. Patients with delirium were compared to those without. | Compared to the group without delirium, people with delirium showed more increase in the H-beta waves (19%, *p* = 0.003), gamma (19%, *p* = 0.006) waves, the tension index (8%, *p* = 0.011), and more decrease in the theta waves (−23%, *p* = 0.016) one week after the surgery. |
| Urdanibia-Centelles | 2021 | Denmark | | NicoletOne^TM^  Version 5.71 (Nicolet, Natus Medical Incorporated) | Delirium | To compare visual analysis and automatic analysis of continuous EEG in diagnosing delirium in septic patients. | Quantitative cross-sectional study with 102 older adults with sepsis in the ICU. Delirium was evaluated six times per day with the Confusion Assessment Method in the ICU. | Delirium was associated with several parameters of the continuous EEG. The AUC is slightly higher for visual analysis (88%) than automatic analysis (74%). The authors concluded that the automatic analysis can complement the visual analysis in delirium diagnosis. |
| Nakamura | 2020 | UK | | Not reported | Sleep - stages | To investigate the validity of in-ear EEG monitoring of sleep. | Quantitative cross-sectional study with 22 adult participants. Sleep was monitored by both the in-ear EEG and PSG for a whole night in their homes. | Accuracy in five sleep stage classification: 74% compared to PSG (k = 0.61). |
| Vacas | 2016 | USA | | SedLine Brain Function Monitor (Masimo Corp., Irvine, California) | Sleep - stages | To validate the device in laboratory setting and to test the feasibility of its use in the ICU. | Quantitative cross-sectional study with three adult outpatients in the laboratory and 23 ICU patients. The system was validated against PSG in the laboratory, where participants were monitored for one night. The mean recording time in the ICU was 19.1 hours. | The system could distinguish sleep from wakefulness and the transition between sleep stages, although the latter is less accurate. |
| Pu | 2021 | Australia | | MUSE 2 (InteraXon Inc., Toronto ON, Canada) | Pain | To assess the feasibility of a headband EEG to monitor pain in long-term care residents with dementia. | Quantitative cross-sectional study with 4 older adults with dementia and chronic pain in a long-term care facility. Each participant was monitored for 10 minutes. The Pain Assessment in Advanced Dementia (PAINAD) scale was administered before and after the measurement. | The detrended fluctuation analysis of EEG signals fluctuated in participants experiencing pain, but not in the participant without pain. |
| Roh | 2012 | South Korea | | developed by the authors | Stress | To develop a portable system to measure stress and mental state. | Quantitative non-randomized experimental study with 10 healthy adults in the laboratory. For 10 minutes, participants wore a headband and did three stressful tasks with rest in between. | Compared to the resting state, the largest lyapunov exponent value (the physiological marker for stress and mental state) decreased to 0.734 and the standard deviation of heart rate variability (HRV), also derived from EEG data, increased to 73ms during stressful tasks. |
| **Electrocardiography (ECG)** | | | | | | | | |
| De jonckheere | 2014 | France | | Developed by the authors | Pain | To present an ambulant device which monitors pain with HRV analysis. | Quantitative cross-sectional study with 12 adults having physical therapy after surgery. Pain was scored with the Visual Analogue Scale (VAS) during the first and second therapy session. | The HRV was correlated with the VAS (*r* = - 0.50, *p* = 0.002). HRV was lower in the VAS > 30 subgroup than in the VAS ≤ 30 one (p=0.002). |
| Grasso | 2018 | USA | | Mortara H12 (Mortara Instrument, Milwaukee, Wis, USA) | Sleep – apnea | To evaluate the portable ECG system in its detection of sleep disordered breathing compared to standard PSG. | Quantitative prospective cohort study with 30 adult patients with the suspicion of sleep disordered breathing. The ECG monitor and PSG were worn simultaneously overnight in the laboratory. | ECG parameters correlated with PSG parameter of apnea (*r* = 0.77 - 0.81). The ECG system was accurate and sensitive but relatively imprecise in detecting the severity of the apnea. |
| Li | 2022 | China | | myBeat-WHS-1 (Union Tool Co., Ltd., Japan) | Stress | To use a wearable ECG monitor to measure workplace stress in nurses. | Quantitative cross-sectional study with 17 healthy nurses during one work day at the hospital. Participants wore the device and filled in the Chinese Nurses Stress Response Scale (CNSRS) after work. | The HRV parameters were correlated with time, work and rest phases, postures, and subscales of the CNSRS (physiological reaction, irritability, social phobia, and fatigue). |
| Miranda | 2017 | USA | | HxM Bluetooth chest belt (Zephyr) | Anxiety | To present a system to detect internal states of anxiety during caregiving tasks. | Quantitative non-randomized experimental study with 10 healthy adults in the laboratory. During 30 min sessions, participants had a relaxation stage and performed cognitive therapy tasks with a simulated person with dementia. Video recordings of each 30s of the session were coded by the participants for the presence of anxiety. | Using inter beat interval as classification feature, the accuracy of the model was 73%. |
| **Electrodermal activity (EDA) monitors** | | | | | | | | |
| Aslanidis | 2018 | Greece | | Med Storm Pain Monitor System (MED Storm® Innovation AS, Oslo, Norway) | Pain | To monitor electrodermal activity changes during pain. | Quantitative cohort study with 25 adult patients under sedation in the ICU. Participants were monitored for four hours. During a 10-second pain stimulus, electrical dermal activity changes in the palm were measured and compared to observation, BIS, cardiovascular and respiratory measurements. | EDA monitoring was more sensitive to pain compared to cardiovascular, respiratory and BIS measurements. |
| De Vries | 2022 | Netherlands | | Sentisock (Mentech, Eindhoven, the Netherlands) | Stress | To validate the sock-integrated EDA sensor in monitoring stress. | Quantitative non-randomized experimental study with 28 healthy adults in the laboratory. Measurements were taken at baseline, during mental stress and physical stress in a 75 min session. Sock-integrated EDA data was compared to wrist EDA measured by Empatica E4 and self-report of the participants using the Self-Assessment-Manikin scale. | Sentisock: F1-score: 0.84, sensitivity: 81%, specificity: 86%, balanced accuracy: 83%  Empatica E4: F1-score: 0.83, sensitivity: 81% , specificity: 84%, balanced accuracy: 82% |
| Leborgne | 2023 | Netherlands | | Developed by the author | Stress | To develop a sock garment to detect stress in people with intellectual disabilities. | Quantitative non-randomized experimental study. Sixty adults with severe intellectual disabilities in long-term care facilities tried on the garment. It was not clear whether the experiment was conducted with other participants in the laboratory, where the sock sensor was compared with Empatica E4 (Empatica Inc, Boston, USA) during sitting, walking, standing, and doing math. | The signal quality was good for neural and stable positions, but more noise was present during movement. The sock sensors recognized EDC changes in stressful situations with an accuracy of 85%. |
| Setz | 2010 | Switzerland | | Emotion Board (developed by the authors) | Stress | To investigate accuracy of EDA in discriminating stress from cognitive load. | Quantitative randomized controlled trial with 33 healthy adults in the laboratory. Participants went through a 2-hour stress session with social and cognitive stress, as well as a 2-hour cognitive load session. | Maximum accuracy of discriminating stress from cognitive load: 83% |
| **Surface electromyogram (sEMG)** | | | | | | | | |
| Carlson | 1996 | USA | | BioPrompt 3000 (Physical Health Devices) | Pain | To determine whether there is difference in ambulatory sEMG signals in the upper trapezius muscle for people with and without pain. | Quantitative case-control study with 10 adult participants with cervical pain and 10 adult participants without pain. Participants were monitored by the ambulatory device in their normal life for three consecutive days during daytime. | No difference in sEMG signals was found between the group with pain and the group without. |
| Yang | 2018 | China, Finland, Austria, France, and Sweden | | Developed by the authors | Pain | To present a facial expression monitoring system for automatic pain assessment. | Quantitative non-randomized experimental study with one healthy adult in the laboratory. The participant mimicked painful expressions, which were compared to frowning and neutral expressions. The duration of the measurement was not reported. | Muscle activities in the face during painful expressions and neutral expressions could be differentiated. |
| **Incontinence sensors** | | | | | | | | |
| Boerakker | 2022 | Netherlands | | Abena Nova (Abena Group, Denmark), WeSense (Seneca Sense Technologies, Canada) | Incontinence | To investigate the use of intelligent incontinence material in nursing homes. | Mixed-methods pilot study with 25 older adults in nursing homes with urinary incontinence. Abena Nova and WeSense were compared to the regular incontinence material. | Abena Nova reduced the use of incontinence materials by 34%, WeSense reduced it by 16%. |
| Cusick | 2003 | UK | | Developed by the authors | Incontinence | To develop and test an incontinence sensor. | Quantitative cross-sectional study with volunteers. No details on the participants were reported. A total of 71 events were simulated, of which 20 simulated incontinence events.* | 5 false-positive events, 2 false negatives events. The authors concluded that the system met the technological requirements. |
| Ouslander | 1998 | USA | | Not reported | Incontinence | To investigate the relationship between incontinence and sleep disruption. | Quantitative cross-sectional study with 73 older adults in nursing homes. Night-time incontinence was recorded both by a device and by staff. | The records of the device (wet/dry status) coincide with the records made by staff. |
| Not reported (This manuscript is the device website) | 2022 | Denmark | | TENA Identifi Sensor Wear (Essity Hygiene and Health AB, Denmark) | Incontinence | To report customer results. | Quantitative cross-sectional study with 324 adults. Further details not reported.* | Leakage was reduced by 66%,  Time saved (per client per year): 86.7 hours, incontinence material changes reduced by 22%, toilet visits reduced by 6%, urine loss reduced by 14%, rubbish reduced by 36%. Clients can use materials with less absorption capacities. |
| Tekcin | 2022 | Tekcin | | Developed by the authors | Incontinence | To develop a humidity sensor to measure urinary incontinence. | Quantitative non-randomized experimental study in the laboratory. No participants were involved. The sensor was tested in a humidity chamber and water was dripped on it to measure the change in resistance. | In the humidity chamber, the resistance decreased as the humidity increased. Change in resistance of the sensor was measurable with 0.1ml of water dripped on it. |
| Toba | 1996 | Japan | | Not reported | Incontinence | To evaluate the effects of wearing a thin-film urinary drainage sensor in the incontinence material on urinary quality of life and nursing work hours. | Mixed-method cross-sectional study with 4 older adults with urinary incontinence in long-term care facility. Incontinence materials were changed regularly (4 times a day) and as needed (as soon as possible after sensor detection) for 4 days each. The wetting times were compared. Working time was recorded and asked through a caregiver questionnaire. | Incontinence material wetting time was reduced from 1 hour 21 ± 11 minutes with the regular change to 9±1 minutes with the sensor. Total working time increased from 52 minutes to 90 minutes because of the change of the sensors. |
| Van der Hurk | 1998 | Netherlands | | Developed by the authors | Incontinence | To evaluate the applicability and validity of the Urine Leakage Recording Device. | Quantitative non-randomized experimental study with 5 older adults. Participants were instructed to turn and void during the experiment. 3 human assessors scored the data generated from the device to determine incontinence events. This was compared with the self-report of participants. The setting was probably laboratory, but not stated in the manuscript. | Interscorer reliability: 0.92. True positive events: 5.3 out of 10, False positive events: 9.3. The authors concluded that the device was not useful. |
| Wang | 2009 | USA | | Developed by the authors | Incontinence | To validate the system to record urinary leakage in women doing intense physical activities. | Quantitative non-randomized experimental study with one adult woman. The sensitivity of the sensor was first tested in the laboratory with a burette dropping urine onto it. Then the participant wore the device during a 25-minute jog, releasing urine at the end. | The lab test suggested that the sensor could always detect a leakage of more than 0.5ml. The jogging experiment demonstrated that the sensitivity of the sensor was not influenced by sweat disturbance. |
| **Multi-modal systems - polysomnography (PSG)** | | | | | | | | |
| Koley | 2013 | India | Alice LE (Philips Respironics) | | Sleep - apnea | To develop an algorism to detect apnea or hypopnea events using airflow data from PSG. | Quantitative cross-sectional study with 36 adult participants with a variety of diseases. 28 participants received one recording session for a night. 8 participants had repeated test. Recordings were conducted at a Center for sleep disorders. The algorism results were compared with full PSG. | Accuracies of event detection: hypopnea: 92%, apnea: 95%, and combined 97%. |
| Supe | 2017 | USA | Capnostream 20 Bedside Monitors (Covidien, Needham, Massachusetts, USA) | | Hypopnea | To implement continuous monitoring to identify alarm conditions of respiratory distress. | Quantitative cross-sectional study with 25 adult patients with obstructive sleep apnea on a post anesthesia care unit. The time of the monitoring ranged from a few hours to several days. | True positive rate: 57%, compared with clinician judgement. True negative rate: 100%. |
| Koskinen | 2021 | Finland | Collare (Nukute Ltd, Finland) | | Sleep - apnea | To validate the Nukute Collare in diagnosing sleep apnea. | Mixed-methods cross-sectional study with 71 adults with or without sleep apnea. Collare measurement was compared with standard PSG.* | 91% sensitivity, 97% specificity, 91% positive predictive value, 97% negative predictive value, and 0.9-1.0 balanced accuracy. |
| Makinen | 2022 | Finland | Collare (Nukute Ltd, Finland) | | Sleep - apnea | To validate the Nukute Collare in screening sleep apnea. | Quantitative cross-sectional study with 41 adults. Collare measurement was compared with standard PSG.* | Accuracy of diagnosing sleep apnea automatically: 88% - 95%, accuracy of automatically classifying apnea severity: 76%. |
| Rotariu | 2013 | Romania | Developed by the authors | | Sleep - apnea | To develop a system to monitor sleep apnea using wireless sensor nodes. | Quantitative cross-sectional study with 3 patients in the hospital. The patients simulated apnea events to test the system. | Simulated apnea events were detected by the system and alarms were triggered. |
| Jungquist | 2019 | USA | ExSpiron 1Xi Minute Ventilation Monitor (Respiratory Motion) | | Hypopnea | To compare Minute ventilation, capnography, and pulse oximetry in identifying respiratory depression and to develop and algorism for early detection. | Quantitative cross-sectional study with 48 adult participants in the post anesthesia care unit. The participants were the devices during their whole stay in the unit. | Minute ventilation and capnography were both effective in detecting respiratory depression, while pulse oximetry was not. A machine learning model could predict an opioid-induced respiratory depression event 10 min in advance with 80% accuracy. |
| Acebo | 1991 | USA | Home monitoring system, brand not reported | | Sleep -apnea | To investigate the reliability and validity of measures of sleep and apnea using the home sleep monitoring system. | Quantitative cross-sectional study with 14 adults (Study 1) and 8 older adults living in nursing facilities (study 2). Study 1: One night, together with standard PSG in the Sleep Disorders Center. Study 2: Four weekly 24-h recordings were made on successive weeks at the participants’ homes. | Study 1: Significant agreement was found for each sleep measure and the apnea index between the home monitoring system and standard PSG. Study 2: There were significant individual differences, and the measurement is reliable for each sleep measure and the number of apneas. |
| Buyse | 2023 | Belgium | Hospital PSG, model and brand not reported | | Sleep -apnea | To investigate whether lowering the cut-off scores for type 3 portable monitoring would benefit the diagnosis and treatment of obstructive sleep apnea. | Quantitative cross-sectional study with a database of 865 adults who started continuous positive airway pressure. Apnea-hypopnea index (AHI) of both the in-hospital PSG and portable monitoring were simulated from the existing data. Calculations of negative tests were performed with different Portable monitoring- respiratory event index (PM-REI) cut-of scores. | Portable monitoring underestimated the hypopnea events by 15 events/hour, compared to in-hospital PSG. Lowering the PM-REI cut-off score for portable monitoring reduced the negative test rate from 57% to 34%. |
| Ravishankar | 2014 | India and USA | ICU bedside monitors, brand not reported | | Hypopnea | To develop an algorithm to detect respiratory distress. | Quantitative cross-sectional study using ICU patient records. Preliminary analysis: 50 records with acute respiratory failure and 48 records without. Extended analysis: 157 records with no acute respiratory distress. | True Positive Rate: 88-92%; False Positive Rate: 6-12%. The algorithm would be able to detect respiratory instability when existing systems would not. |
| Li | 2021 | China, USA | Nox-T3 (Nox Medical, Reykjavik, Iceland) | | Sleep – disordered breathing | To evaluate the portable monitor in diagnosing sleep disordered breathing. | Quantitative cross-sectional study with 84 adults with chronic heart failure. Participants were monitored at home for one night. Another measurement was conducted with both PSG and the portable monitor in the laboratory. | Diagnosing sleep disordered breathing at ≥ 5 AHI events per hour: 87% sensitivity, 77% specificity.  Detecting of Cheyne-Stokes respiration: 95% sensitivity, 91% specificity, compared to PSG. |
| To | 2021 | China | Nox-T3 (Nox Medical, Reykjavik, Iceland) | | Sleep - apnea | To test the reliability of Nox-T3 in diagnosing sleep apnea in people with major diseases. | Quantitative cross-sectional study with 74 adults with psychiatric illnesses, stroke, ischemic heart diseases, chronic kidney diseases or other major diseases, suspected of having obstructive sleep apnea. Participants were simultaneously monitored by standard PSG and Nox-T3 overnight for at least four hours in the hospital. | Nox-T3 respiratory event index was significantly lower than the PSG AHI. Nox-T3 can reliably diagnose obstructive sleep apnea, but can underestimated the severity. |
| Xu | 2017 | China | Nox-T3 (Nox Medical, Reykjavik, Iceland) | | Sleep - apnea | To validate the portable monitor in the diagnosis of sleep apnea. | Quantitative cross-sectional study with 80 adults. Participants were monitored at home for one night. Another measurement was conducted with both PSG and the portable monitor in the laboratory. | At ≥ 5 AHI events per hour: 95% sensitivity, 69% specificity.  At ≥ 15 AHI events per hour: 93% sensitivity, 85% specificity, compared to standard PSG |
| Wang | 2015 | China | Oximeter (Konica Minolta, Japan) | | Sleep - apnea | To evaluate the use of oximeter in diagnosing obstructive sleep apnea. | Quantitative cross-sectional study with 244 patients with obstructive sleep apnea hypopnea syndrome in the hospital. Participants were monitored overnight with both an oximeter and a full PSG. | Oximeter demonstrated a sensitivity of 68% and a specificity of 73% in diagnosing obstructive sleep apnea hypopnea syndrome, compared with PSG. |
| Chang | 2019 | China, USA | PM, Nox-T3 (Nox Medical, Reykjavik, Iceland) | | Sleep -apnea | To evaluate a portable monitor in diagnosing obstructive sleep apnea. | Quantitative cross-sectional study with 90 adults with chronic obstructive pulmonary disease. Participants were monitored at home for one night. Another measurement was conducted with both PSG and the portable monitor in the laboratory. | 95% sensitivity and 78% specificity in diagnosing obstructive sleep apnea compared to a standard PSG. |
| Alvarez | 2017 | Spain | Reslink^TM^ module (ResMed Inc. SanDiego, CA) | | Sleep - hypopnea | To evaluate the reliability of the built-in software compared to ventilatory Polygraphy. | Quantitative cross-sectional study with 26 adults with stable obesity hypoventilation syndrome under nocturnal non-invasive ventilation. Participants were monitored at home for a full night. | The built-in analysis is reproducible and reliable to assess quality of non-invasive ventilation when compared with Polygraphy (*r*^2^ = 0.71, *p* < 0.001), but is less consistent than manual scoring. |
| Ganglberger | 2022 | USA | Respiration belt: AirGo (brand not reported), standard PSG: Natus System (brand not reported) | | Sleep -apnea | To develop an algorithm to detect sleep-related respiratory abnormalities measured by a wearable device. | Quantitative cross-sectional study with 404 overweight adults. Participants were monitored with both devices for at least one night in the laboratory. | The AHI from the PSG, labeled by experts, correlated with those predicted by models using data from the respiration belt and oxygen saturation (*r* = 0.96), respiration-only (*r* = 0.78), and saturation only (*r* = 0.93). The AUCs were 94%, 86%, and 82% for the receiver operating characteristic curves, and 48%, 32%, and 51% for the precision-recall curves. |
| Tedeschi | 2013 | Italy | Somtè PSG (Compumedics, Abbotsford, Victoria, Australia) | | Sleep - apnea | Compare home unattended portable monitoring and automatic continuous positive airway pressure titration to an attended in-laboratory program. | Randomized controlled trial with 131 adults with high suspicion of obstructive sleep apnea. 66 participants received home monitoring and 65 received in-laboratory standard PSG. | Reliability: 97% for the home portable monitoring. 2 out of 66 recordings had low quality. |
| Polese | 2013 | Brazil | Stardust II® (Philips Respironics, Inc., Murrysville, PA, USA) | | Sleep - apnea | To evaluate the effectiveness of a type 3 portable monitor. | Quantitative cross-sectional study with 43 older participants. Sleep was assessed for two nights, one at home with the portable monitor (Stardust), the other at the hospital with Stardust and standard PSG. | No difference in the apnea–hypopnea index values between the portable and standard PSG.  Good correlation between standard PSG and portable monitor (r=0.67) and the combination (r=0.84).  The area under the receiver operator curve indicated good sensitivity and a positive predictive value for AHI with cutoffs of 5, 15, and 30 and good specificity and negative predictive value for AHI values above 15. |
| Lazazzera | 2022 | France | UpNEA (developed by the authors) | | Sleep - apnea | To present a system aimed to predict respiratory and cardiovascular disorders. UpNEA was part of the whole system. | Quantitative cross-sectional study with 96 adults with apnea/hypopnea syndrome in a hospital. Participants wore the glove-life device for a night. It was not reported with which device the results were compared. One healthy adult also wore the device for four nights.* | Within the patient group, the device detected central and obstructive apnea or hypopnea with a sensitivity of 73% - 87%, a specificity of 55% - 68%, and an accuracy of 65% - 72%.  The data from the healthy adult showed a specificity of the device in detecting apnea or hypopnea was 96%. |
| Kang | 2012 | China | WatchPAT (Itamar Medical, Israel) | | Sleep - apnea | To validate WatchPAT in diagnosing obstructive sleep apnea hypopnea syndrome. | Quantitative cross-sectional study with 35 adults complaining of snoring. Participants were monitored with Watch PAT and standard PSG simultaneously in the laboratory. | The apnea hypopnea index registered by WatchPAT correlated with PSG (*r* = 0.96, *p* < 0.001). Sensitivity = 92%, specificity = 100%. Accuracy is lower in patients with a high BMI. |
| Lachenmeier | 2022 | Germany | SleepU (Wellue Health, Shenzhen Viatom Technology Co., Ltd., Shenzhen,  China) | | Sleep - dyspnea | To present a case where wearable oximetry device with haptic feedback was used to treat sleep disturbances in a person with lung cancer. | Case report with a home-dwelling older adult with lung cancer and sleep disturbances due to drops of oxygen saturation during sleep. The device vibrated when the oxygen saturation dropped. The recording of one whole night was reported. | The detection of decrease in oxygen saturation was reliable. By waking the patient with the vibration, events of oxygen saturation drop were reduced, so did sleep disturbances and anxiety symptoms associated with them. |
| Arnal | 2020 | France | Dreem Headband (developed by the authors) | | Sleep - stages | To validate the Dreem Headband, containing EEG, pulse oximetry and accelerometer in determining sleep stages. | Quantitative cross-sectional study with 25 healthy adults at a sleep center. The sleep stages determined by Dreem Headband was compared with standard PSG. | The error of Dreem Headband compared with standard PSG:  EEG signals: 10% - 16%  Heart rate: 1.2 ± 0.5 beats per minute  Breathing frequency: 0.3 ± 0.2 cycles per minute  Respiration rate variability: 3.2 ± 0.6% |
| Cooray | 2021 | UK | Signals from standard PSG (model and brand not reported) | | Sleep – stages and rapid-eye-movement sleep behavioral disorder (RBD) | To develop a RBD screening tool with minimal sensors. | Quantitative cross-sectional study with 50 adults with RBD and 50 healthy controls. Participants were monitored for one night with standard PSG for one night. The results of the algorithm were compared to results based an earlier algorithm using EEG, electrooculogram (EOG), and EMG. | The optimal model combined EOG and EMG and could classify sleep stages with 0.57 ± 0.19 kappa (3 stage) and detect RBD detection with an accuracy of 90% (sensitivity 88% and specificity 92%). |
| Hirnoven | 1997 | Finland | 8-channel 9000-11 recorder (Oxford Medilog) | | Sleep - stages | To develop an algorism to score drowsiness and sleep onset. | Quantitative cross-sectional study. An algorism was developed based on 30 min recording at sleep onset of seven healthy adult participants and a 6-hour daytime recording, including drowsy and sleep episodes, of seven adult patients with obstructive sleep apnea syndrome was used to test the algorism. * | Total agreement between the visual and computer scorings: 92%  Non-REM sleep: 64% sensitivity, 82% specificity.  Drowsy stages: 19% sensitivity, 61% specificity.  Alpha activity detection: 5% sensitivity, 11-13% specificity. |
| Altini | 2021 | Finland | Oura Ring, Gen2M (Oura Health Ltd, Oulu, Finland) | | Sleep - stages | To test the accuracy of sleep stages determination by Oura Ring, combined with machine learning. | Quantitative cross-sectional study with 106 adults. In total, 440 nights of sleep were monitored by Oura Ring and standard PSG. Some of the recordings took place at home, the setting of other recordings was not reported. Machine learning models were build using different numbers of parameters derived from Oura Ring signals. | Accuracy for 2-stage detection (sleep, wake) with accelerometer data was 94%. Including other parameters (temperature, HRV, and circadian features) increased the accuracy to 96%.  Accuracy for 4-stage detection (light NREM sleep, deep NREM sleep, REM sleep, and  wake) with accelerometer data was 57%. Including other parameters increased the accuracy to 79%. |
| Ghorbani | 2022 | Singapore | Oura Ring, Gen2 and Gen3 (Oura Health Ltd, Oulu, Finland) | | Sleep - stages | To compare two generations of Oura Ring algorithms in classifying sleep stages. | Quantitative cross-sectional study with 58 healthy adults, each went through 3 nights of home monitoring with standard PSG and Oura Ring. | Gen3 accuracy: 92.6%, sensitivity: 94.9% (0.03), specificity: 78.5%. Gen3 algorithm outperformed Gen2 and is comparable with PSG in measuring sleep latency, light sleep, rapid eye movement, and wake after sleep onset durations. |
| Kinnunen | 2020 | Finland | Oura Ring Gen3 (Oura Health Ltd, Oulu, Finland) | | Sleep -heart rate | To validate the Oura Ring in monitoring nocturnal heart rate. | Quantitative cross-sectional study with 49 healthy adults. Recordings were made at home with both the Oura Ring and an ECG during the night. | High correlation between the Oura Ring and ECG on the average heart rate (*r^2^* = 0.996) and HRV (*r^2^* = 0.980). |
| **Multi-modal systems - with environmental sensors** | | | | | | | | |
| Au-Yeung | 2020 | USA | | Ambient sensors: model not reported (NYCE Sensors, Vancouver, BC),  Pressure mats: model not reported (Emfit, Finland),  Actigraphy: Actiwatch Spectrums (Philips Respironics, Murrysville, PA),  Environmental sensors: Thunderboard Sense 2-SLTB004A (Silicon Labs, Austin, TX) | Agitation | To provide a proof of concept of monitoring and predicting agitation in memory care facilities with by monitoring the activity, sleep, and the environment (temperature, light, sound, humidity). | Quantitative case study with one adult with dementia in a memory care unit. The participant was continuously monitored by the system for 138 days. Agitation was recorded by nurses. | Sleep, total activity, humidity, ambient sound during the night, humidity and light and in the living room of the previous evening correlated with the presence of agitation at night (*p* < 0.05) |
| Bankole | 2020 | USA | | BESI system (developed by the authors) | Agitation | To develop and validate a home monitoring system for agitation and its environmental triggers in people with dementia. The system includes an actigraphy and environmental sensors for acoustic, light, humidity, barometric pressure, and movement. | Mixed-method study with two healthy volunteers and 12 community-dwelling people with dementia and their family caregivers. The BESI system was first tested in the laboratory and then applied in each home of the people with dementia for 30 days. Family caregivers recorded the activities and agitation events of the people with dementia. | Environmental factors (temperature, pressure, and humidity) strongly correlated with agitation (*r* = 0.70 to 0.85, *p* = 0.05). However, the predictors were person-specific. |
| Khan | 2019 | Canada | | Developed by the authors | Agitation | To develop a system to automatically detect agitation in people with dementia. The proposed system includes actigraphy, EDA monitor, pressure mat, video cameras, environmental motion sensors, and door sensors. | Quantitative cross-sectional study with two older adults with dementia at a rehabilitation institute. The participants were monitored for 15 and 13 days respectively. Agitation episodes were noted by nurses. Different algorithms were tested using different combinations of sensor data. | The highest AUC (89%) was achieved by combining actigraphy, skin temperature, and EDA data. |
| Davoudi | 2019 | USA | | Developed by the authors | Delirium | To pilot test a system that includes a camera, three actigraphy, a light sensor, and a microphone in the ICU. | Quantitative cross-sectional study with 17 older adults in the surgical ICU, nine of them had delirium. Participants were monitored continuously for a maximum of seven days. | Participants with and without delirium significant differed in facial expressions, functional status entailing extremity movement and postures, and environmental factors including the visitation frequency, light and sound pressure levels at night. |
| **Multi-modal systems – other multi-modal systems** | | | | | | | | |
| Chen | 2019 | Singapore | | Study 1: chest-worn biosensor (model and brand not reported), Study 2:  Everion® CD, analyzed by the Biovitals^TM^ analytics platform | Change in vital signs, hypoxia | To present an analytic platform to monitor the changes in vital signs, including heart rate, respiration rate and activity. | Quantitative non-randomized experimental studies. The first study used data of 26 older adults with heart failure after discharge. They were monitored for seven to 60 days. Disturbance was added by the researchers to a few days of the data to validate the system. In the second study, 50 healthy adults wore the device for four days in their daily life and for two hours in a hypobaric chamber where they performed controlled exercises. The Biovitals results of daily life was compared with those in the chamber. | Study 1: The system could recognize randomly added disturbance to the data with an AUC of 80%-94%.  Study 2: The system could distinguish the daily life from the hypobaric chamber condition with an AUC of 99%, a sensitivity of 91%, and a specificity of 98%. |
| Ramirez-Moreno | 2021 | Mexico | | Ultracortex “Mark IV” EEG headset, (OpenBCI, New York, NY, USA) Empatica E4 (Empatica, Milano, Italy) | Mental fatigue | To develop a model to detect mental fatigue in workplace. | Quantitative non-randomized experimental study with 17 healthy adults in the laboratory. Participants were monitored for 30s with eyes open, 30s with eyes closed, and four minutes during an auditory oddball task. Correlations to self-reported mental fatigue levels (using the fatigue assessment scale) were calculated to find the best predictors. | EEG features correlated with the self-report of mental fatigue. The parameters measured by Empatica E4 did not have statistically significant correlations. The best model using three EEG features had an accuracy of 88%. |
| Gelinas | 2021 | Canada | | Nociception Level (NOL) Index PMD200^TM^ (Medasense Biometrics Ltd, Ramat Gan, Israel) | Pain | To test a system to monitor pain in patients after an operation. The device is worn on the finger and monitors EDA, and heart rate. | Quantitative non-randomized experimental study with 70 participants in the ICU after cardiac surgery. The NOL index calculated by the system was compared to self-reports of pain and anxiety (0-10 numeric rating scale), as well as observation of pain using the Critical-Care Pain Observation Tool (CPOT) measured before and during a non-nociceptive procedure and before, during, and after a nociceptive procedure. | The NOL index was higher during nociceptive procedures and correlated with self-reported pain intensity and unpleasantness. It did not correlate anxiety and CPOT. The NOL recognized pain with a self-reported intensity above 1 (out of 10) with an AUC of 70%, sensitivity of 76%, and specificity of 61%. For pain intensity above 5, the AUC was 74%, the sensitivity was 85%, and the specificity was 75%. |
| Jiang | 2019 | Finland, USA, Austria | | Developed by the authors | Pain | To develop a system to continuously monitor pain by measuring respiration rate, heart rate, EDA, and face sEMG. | Quantitative non-randomized experimental study with 30 healthy participants in the laboratory. Participants received electrical or thermal pain stimuli on the forearm of ring finger while being monitored by the system. The monitoring results were compared to self-report of no pain, mild pain, or moderate/severe pain based on a VAS. | They system could classify pain in three severity categories with an accuracy of 70.6%. Respiration rate, heart rate, EDA correlate more strongly with pain intensity than face sEMG. |
| Lai Kwan | 2019 | Canada | | Triple Point Sensor (TPS) (ought Technology Ltd. ) | Significant moments | To present a system which detects significant moments of people with dementia and their family caregivers by measuring EDA, skin temperature, and heart rate. | Quantitative cross-sectional study with three older people with dementia and their family caregivers. Participants wore the sensor during 45-min sessions of a movement program for 8 times. Significant moments, carrying physical, emotional, or interpersonal significance, were detected by the system and participants were asked to watch 20s recordings of these moments and evaluate whether these were significant or memorable. | The agreement between the automatic detection and subjective report was 70%. |
| Miranda | 2016 | USA | | Heart rate: Hxm (Zephyr); EDA: Empatica E3; EEG: Muse band | Anxiety | To describe an experiment eliciting anxiety in informal caregivers for people with dementia, while measuring heart rate, EDA, and EEG. | Quantitative non-randomized experimental study with 10 healthy adults in the laboratory. During 30 min sessions, participants had a relaxation stage and performed therapy with a simulated person with dementia. The level of anxiety was recorded by the participants and researchers. Models were developed using the physiological parameters to predict the presence of anxiety. | The average precision of different models using EDA and inter-beat interval was 78%. The best model used only inter-beat interval, reaching a precision of 80%. Specificity was 38% on average and 47% for the inter-beat interval model. |
| Rajasekaran | 2011 | USA | | Portable Autonomous Multisensory Intervention Device (PAMID, developed by the authors) | Agitation | To test the reliability of the chest belt which integrated measurements for heart rate, skin temperature and EDA, and to evaluate its appearance and comfortability. | Quantitative non-randomized experimental study with six community-dwelling older adults in the laboratory. During a 40-minute experiment, participants took four sets of STROOP tests while being monitored by the device and conventional instruments. | PAMID was sensitive in detecting changes in heart rate, skin temperature and EDA. |
| Spasojevic | 2021 | Canada | | Empatica E4 | Agitation | To detect agitation in people with dementia using a multi-modal wearable device which monitored movement, blood volume pulse, EDA, and skin temperature. | Quantitative cross-sectional study with 17 people with dementia in a dementia unit. Participants wore the device in day time for a maximum of two months. Agitation events were noted with observation through video cameras. | Multi-modal prediction models were better than single-model ones. The best model had an AUC of 82%. Skin temperature was the least relevant signal. |
| Choi | 2012 | USA, Qatar | | Developed by the authors | Stress | To validate a system to distinguish stress from relaxation by monitoring respiration, HRV, EDA, and sEMG. | Quantitative non-randomized experimental study with 10 healthy adults in the laboratory. During a 48-minute experiment, participants performed various mentally stressful tasks, with deep breathing in between. | The system could distinguish mental stress from relaxation with a classification rate of 81%, a true positive rate of 87%, and a true negative rate of 71%. |
| Wijsman | 2011 | Netherlands | | Developed by the authors | Stress | To find physiological parameters that can best predict mental stress. ECG, respiration, EDA, and sEMG were measured. | Quantitative non-randomized experimental study with 30 healthy adults in the laboratory. During an experiment of around 20 minutes, the participants performed three cognitive tasks with time pressure and distraction. Social stress was also induced. Resting periods were scheduled between the tests. | Using seven physiological features, the system could distinguish stressful periods from rest periods with an accuracy of 80%. |
| Wu | 2012 | South Korea | | Developed by the authors | Stress | To test the effectiveness of stress reduction training with a wearable biofeedback system, consisting of ECG, Pulse rate, respiration, ambient and breath temperature measurements. | Quantitative non-randomized experimental study with 67 adults in the laboratory and during a 3-week biofeedback training for stress reduction. For the validation of the device, several adults were measured by it and a commercially available PolyG-A system in the laboratory. | The proposed device produced similar ECG, photoplethysmography and respiration signals compared to the commercial system. HRV and pulse rate variability were extracted accurately. |
| **Non-contact monitoring systems - pressure mats** | | | | | | | | |
| Boissy | 2011 | Canada | | Developed by Université de Sherbrooke, Québec, Canada | (risk of) pressure ulcer | To present the functioning of two type of carbon nano tube films (multi-layered vs purified) in detecting pressure. | Quantitative experimental study without participants. The electrical and mechanical properties of the films were tested. | The loosely woven, multi-layered film had lower sensitivity (resistance change was 8 times higher) compared to the purified film. The purified film was less affected by the mechanical noise of the testing system (noise ratio 5% vs. 50%). |
| Dietz-Terjung | 2020 | Germany | | VitaLog (SWGSportwerkGmbH&Co. KG, Dortmund, Germany) | Sleep – disordered breathing | To assess the accuracy of VitaLog in estimating respiratory rate variability. | Quantitative cross-sectional study with 103 adults referred to the sleep lab. The VitaLog data were compared to nasal flow signal obtained by PSG.* | The VitaLog estimation of respiration rate variability had a correlation of *r* = 0.99 and a bias of 0.2 inhale-exhale cycles per minute compared with nasal flow signal. The Mann-Whitney U test showed no significant difference between participants with or without sleep disturbed breathing. |
| Kobayashi | 2012 | Japan | | SD-101 (manufacturer not reported); Percutaneous oxygen saturation (SpO2 )sensor: SX Module SX-2007 (Kenzmedico Co. Ltd., Saitama, Japan) | Sleep – apnea | To investigate whether adding a SpO2 measurement increases the accuracy of recognizing mild to moderate obstructive sleep apnea syndrome by SD-101. | Quantitative cross-sectional study with 60 adult patients from an out-patient clinic. Overnight sleep was simultaneously monitored by PSG and SD-101 at the laboratory. | Adding SpO2 measurement improved the accuracy of the SD-101 for OSAS detection for AHI >15 (sensitivity: 97% vs. 88%; specificity: 90.5 % vs. 85.7 %, compared to the SD-101 alone).  The SD-101 measurements significantly correlated with the PSG data (SD-101 alone: *r* = 0.871, *p* < 0.0001; SD-101 with SpO2: *r* = 0.965, *p* < 0.0001). Bland–Altman plots showed a smaller dispersion for the SD-101 with SpO2 than for the SD-101 alone. |
| Motoi | 2009 | Japan | | Developed by the authors | Sleep – apnea and hypopnea | To study the applicability of non-contact sensors in a hospital room. Only the results about the pressure mat were presented in this manuscript. | Quantitative cross-sectional study with two adults with sleep apnea in the hospital. Participants were monitored for one night with both the pressure mat under the pillow and a standard PSG. | There was good agreement on the number of apnea or hypopnea events per hour between the pressure mat and the PSG. |
| **Non-contact monitoring systems – video/facial expression analysis** | | | | | | | | |
| Becouze | 2007 | New Zealand | | Camera: Canon IXUS 40 digital camera; Algorithm developed by the authors | Agitation | To develop an algorithm to detect grimacing independent of the head movement. | Quantitative non-randomized experimental study with two simulated videos of the face, one grimacing and the other with a neutral expression. | The algorithm could successfully track the reference point on the forehead and identify the face area when the head was moving. The grimacing level determined by the system corresponded with observation. |
| Palestra | 2020 | Italy | | RGB cameras; Algorithm developed by the authors | Emotions | To evaluate a robot-facilitated memory training in its effects of engaging the participants. | Quantitative cross-sectional study with 21 adults with Mild Cognitive Impairment. Videos of an hour were taken during robot-facilitated group memory training sessions and emotions were identified by automatic facial expression analysis. | The system could detect all faces, including partially covered faces (success rate 56%), and recognize emotions reliably when the face was detected. |
| Castillo | 2020 | Canada | | Algorithm: FaceReader version 8 (Noldus Technology Information) | Pain | To validate the FaceReader in its estimation of pain and heart rate. | Quantitative cross-sectional study with pre-existing video recordings of two older long-term care residents with dementia and two community dwelling older adults. FaceReader automatically analysed pain based on facial expressions and estimated the heart rate. Pain was also manually scored using the Facial Action Coding System (FACS) and heart rate was estimated with the well-established Video Magnification (VM) algorithm. | FaceReader rating of pain scores strongly correlated with FACS during and around facial pain expression (*r* = 0.99 – 1.00). Its heart rate estimation strongly correlated with VM (*r* = 0.99 – 1.00). |
| Rezaei | 2021 | Canada | | Algorithm developed by the authors | Pain | To develop a technology to automatically recognize pain in long-term care facilities. | Quantitative experimental study with pre-existing video recordings of the faces of 25 adults with shoulder injury, 47 community-dwelling older adults, and 48 older adults with severe dementia in long-term care facilities. Automatic recognition of pain was compared with Prkachin and Solomon Pain Index (PSPI), scored by trained human annotators. | The algorithm outperformed baseline in recognizing pain. Pain prediction with the final model correlated with PSPI (*r* = 0.48 – 0.70), had an average precision of 0.71 (people with dementia) and 0.84 (in older adults without dementia). The AUC was 0.85 – 0.86. |
| **Non-contact monitoring systems - infrared** | | | | | | | | |
| Casaccia | 2019 | Italy | | Passive InfraRed motion sensor (brand not reported) | Sleep - stages | To test the possibility of classifying sleep stages using infrared signal. | Quantitative case report on 25 nights of sleep data of one healthy adult. The sleep stages classified by the Passive InfraRed system was compared to the sleep pattern measured by a Ballistocardiographic bed sensor (Nokia Sleep bed sensor) under the mattress. | Passive InfraRed sensor could detect the waking states (R = 0.67 - 0.86, R^2^ = 0.45 - 0.69) better than REM (R = 0.40, R^2^ = 0.16), deep sleep (R = -0.03, R^2^ = 0.00). and light sleep (R = 0.61, R^2^ =0.37). |
| Tejedor | 2020 | Spain | | Camera: FLIR E60bx | Thermal comfort | To propose a model to determine indoor thermal comfort for nursing home residents. | Quantitative case study with 15 older adults in a nursing home during winter. Participants were monitored for five minutes. A thermal questionnaire was filled in by the participants. | The proposed numerical model was reliably. The temperature of the nose could reliably indicate feeling cold. The self-report of the participants was concluded as not reliable. |
| **Non-contact monitoring systems - radar** | | | | | | | | |
| Hsu | 2017 | USA | | EZ-Sleep (developed by the authors) | Sleep | To present a radio-based sleep monitor. | Quantitative cross-sectional study with 10 healthy community-duelling adults. More than 100 nights of sleep was monitored by EZ-Sleep and compared to a wearable SleepProfiler and sleep diary. | Compared to SleepProfiler, the EZ-Sleep had an average error of 3.15 min in estimating time in bed, 10.3 min in total sleep time, 4.9 min in sleep latency, 8.2 min in wake after sleep onset, and 2.8% in sleep efficiency.  EZ-Sleep can simultaneously monitor more than one people, although there can be a little disturbance if two people are sleeping close to each other. |
| Resuli | 2021 | USA | | Vayyar radar system | Sleep -respiration rate | To test the accuracy of respiration rate estimation in different sleeping positions with a radio frequency sensor. | Quantitative non-randomized experimental study with five healthy adults in a laboratory. Participants took different sleeping positions, each for at least six minutes. The radio frequency based estimation of breathing was compared with a respiration belt. | The accuracy of respiration estimation by the radar system was 92% in back postures, 84% in side postures, 76% in stomach position, 90% with a blanket covering the participant, 87% when sleeping on the back with the face turned sideways, and 86% in the sitting position. |
| Schnellenberge | 2020 | Germany | | Developed by the authors | Vital signs | To present a dataset with radar signals of the vital signs with synchronized reference. | Quantitative non-randomized experimental study with 30 healthy adults in the laboratory. Participants lay on a tilted table and five scenarios triggering the automatic nervous system and hemodynamics were simulated. Radar recording of the chest was conducted simultaneously with ECG, impedance cardiogram, and blood pressure. | The interbeat-intervals measured by the radar highly correlated with the ECG data (*r* = 0.96, *p* < 0.001) |
| **Non-contact monitoring systems - multiple sensors** | | | | | | | | |
| Dimitrievski | 2021 | Macedonia, Portugal, Spain | | Piezoelectric sensor: (Angelcare, Montreal, QC, Canada), passive infrared sensors (Sunfounder, Shenzhen, China) | Sleep | To present a non-invasive system that monitors movement during sleep, aiming to detect sleep disturbances due to COVID-19. | Quantitative cross-sectional study with adults with COVID-19 in the hospital. Participants were monitored for eight hours. | Data from the Piezoelectric sensor correlated with the infrared sensors (*r* = 0.32 - 0.43). The five infrared sensors also correlated with each other (*r* = 0.83). There was built-in delay in the infrared sensors and oscillation in the piezoelectric sensor, but these could be adjusted with the post-processing of the data. |
| Ravindran | 2023 | UK | | Radar: Somnofy (VitalThings, Norway); Pressure mats: Withings Sleep Analyser (WSA, Withings, France), Emfit-QS (Emfit Ltd, Finland) | Sleep | To compare three non-contact sleep monitors with PSG and actigraphy (Actiwatch Spectrum). | Quantitative cross-sectional study with 35 older adults, some with sleep apnea. Participants used the non-contact devices at their own homes for 8-14 days and had one overnight measurement with all the devices in the laboratory. | Compared to PSG, all three devices overestimated total sleep time (bias > 90 min) and sleep efficiency (bias > 13 %), and underestimated wake after sleep onset (bias > 50 min). Bedside radar accurately measured sleep onset latency (bias < 6 mins) while the under-mattress devices overestimated it (bias >16 mins).  These performances were less optimal than actigraphy. The bedside radar performed better in discriminating sleep vs wake (Matthews Correlation Coefficient (MCC) [mean and 95% CI] = 0.63 [0.57 0.69]) than the under-mattress devices (MCC = 0.41 [0.36 0.46]; Emfit-QS = 0.35 [0.26 0.43]). Accuracy of identifying REM and Light sleep was poor for all three devices, but deep sleep was predicted with moderate accuracy (MCC >0.45) by both Somnofy and Withings Sleep Analyser. |
| Kroll | 2020 | Germany | | Camera: Mini-Webcam (Conrad Electronics SE, Hirschau, Germany);  Acoustic sensor: ME32 (Olympus Imaging Europa GmbH, Hamburg, Germany)  Pressure mat: SafeBed IP system (Emfit® Ltd., Vaajakoski, Finland) | Agitation | To validate the non-contact monitoring system and a tent-like shelter for people with dementia. | Quantitative cross-sectional study with six healthy adult volunteers in the laboratory and 19 patients, most of whom had dementia, in the emergency department and a geriatric-psychiatric ward. Participants were monitored for two hours, the first hour without the tent-like shelter, the second hour with it. | In the laboratory test, the measurement of heart rate was reliable between the first and second hour, but that of respiration rate was not. The camera and mattress registration of movement complimented each other. The measurements in the emergency department and geriatric-gerontopsychiatric ward were reliable. |
| *Notes:* Adults = mean age between 18 and 65; Older adults = mean age over 65; ICU = intensive care unit  References can be found in Supplement IV.  *The setting was not reported. | | | | | | | | |
