# Supplementary material for: Noninvasive monitoring technologies to identify discomfort and distressing symptoms in persons with limited communication at the end of life: a scoping review
Source: BMC Palliat Care. 2024 Mar 21;23:78. doi: 10.1186/s12904-024-01371-0 (PMC10956214; doi:10.1186/s12904-024-01371-0)
Supplement: Supplementary file 2 — Additional file 2: eTable 2 Manuscripts reporting on the acceptability or feasibility of the monitoring technologies to detect distress and discomfort. [file 12904_2024_1371_MOESM2_ESM.docx]

**Supplement II of “Noninvasive monitoring technologies to identify discomfort and distressing symptoms in persons with limited communication at the end of life: A scoping review”**

| eTable 2  *Manuscripts reporting on the acceptability or feasibility of the monitoring technologies to detect distress and discomfort* | | | | | | | | |
| --- | --- | --- | --- | --- | --- | --- | --- | --- |
| **Actigraphy** | | | | | | | | |
| **First author** | **Year** | **Country** | | **Monitoring technology (model and brand)** | **Symptom monitored** | **Aim of study** | **Methods: Study design; Participants being monitored; Setting; Procedures; Participants evaluating the technology;** | **Acceptability or feasibility of the technology** |
| Knuff | 2019 | USA | | wGT3x+ activity monitors (Actigraph,  LLC, Pensacola, FL) | Agitation | To evaluate the feasibility and validity of actigraphy as a measurement of agitation in dementia. | Quantitative cross-sectional study. 20 older adults with dementia living in long-term care facilities wore the wristband continuously for a minimum of 24 hours and maximum of 7 days. Feasibility was evaluated by the researchers. | Actigraph monitors recorded for almost 1 week. Agitation severity did not influence compliance. The authors concluded that multi-day continuous actigraphic monitoring is feasible. |
| Favela | 2020 | Mexico | | Fitbit Charge 2 HR, Fitbit Alta (Fitbit Inc, CA, USA) | Behavioral symptoms of dementia | To evaluate an intervention program lead by a social robot. | Mixed methods non-randomised experimental study with 10 older adults in a long-term care facility. The wristwatch form of actigraphy was worn for 54 days on average. Feasibility was evaluated by the researchers. | One participant refused to wear the device at night. Two other participants needed persuasion to take the device off for charging. One participant feared losing or damaging the device and discontinued wearing it. The other participants did not experience anxiety about the device. |
| LeBlanc | 2022 | USA | | MisFit Shine | Sleep | To describe the experience of using a sleep monitor for sleep self-management. | Qualitative study with 25 community-dwelling older adults with sleep disturbances. The participants wore the device for four weeks and received an interview afterwards. | Regarding the device, participants could understand how it worked and considered it easy to use. The plain design was appreciated, although some participants wanted the device to look more like a watch and found it bothersome having to tap it to see the time. The device was comfortable and light-weighted. The wrist clamp and battery life could be improved. There was concern about privacy of the data and some did not trust the results of the device. Both financial and time costs were important issues to consider. More than half of the participants had the intention to continue using the device. |
| Svetnik | 2021 | USA | Garmin Vivosmart | | Sleep | To investigate the usefulness of a wearable in measuring treatment effects of insomnia in people with dementia. | Randomized experimental study with 285 older adults with Alzheimer’s disease and insomnia. During insomnia treatment of four weeks, participants were instructed to wear the device all the nights at home. Three overnight measurements with standard polysomnography (PSG) were performed in the laboratory at screening, baseline, and at the end of the treatment. The challenges of using the device were reported by the researchers. | 74% of the participants had usable data of the device, which is at least three nights of measurements in a week. The challenges were patient compliance, battery life and Bluetooth connectivity between the watch and tablet. |
| Van Dijk | 2012 | Netherlands | | Actiwatch AW7 (Cambridge Neurotechnology Ltd, Cambridge, UK) | Sleep | To evaluate the feasibility of using actigraphy to monitor sleep in older people with intellectual disability. | Quantitative cross-sectional study. 563 older adults with intellectual disability wore the actiwatch continuously for a maximum of 14 days. Feasibility was evaluated by the researchers.* | A successful measurement was obtained from 200 (35%) of the participants. Unsuccessful measurements were mainly due to problems wearing the device and incomplete bedtime information. 354 (92%) of those who started wearing the device wore it for more than 7 days. |
| **Brain activity monitors – Bispectural Index (BIS)** | | | | | | | | |
| Gambrell | 2005 | USA | | Not reported | Depth of sedation | To present a case where the BIS monitor is used successfully in the ICU. | Case study of a 78-year-old woman with intraventricular haemorrhage. She was monitored in the last few hours of her life in the ICU. The nurse and family expressed their opinions on the device. | The nurse saw BIS as an additional tool to help titration and to educate the family. The family is more confident of a comfortable death and are pleased to see the BIS values fluctuate when they talked to the patient. |
| Pedrao | 2020 | Brazil | | BIS™ Vista Monitoring System (Covidien LLC, Mansfield, United States) | Sleep | To test the feasibility of using BIS to evaluate sleep in critically ill patients. | Quantitative cross-sectional study with 29 lucid patients in ICU. BIS monitoring was performed for a whole night (12 hours). The participants filled in the Richards-Campbell Sleep Questionnaire (RCSQ) the next morning. The feasibility was evaluated by the researchers. | Among 49 patients who were eligible for the study, seven refused to participate, two could not be monitored because of delirium, five could not be monitored because of other procedures they underwent, five dropped out because of discomfort of the sensors or displacement of the sensors.  The authors concluded that BIS had limited feasibility in lucid patients in the ICU. |
| **Brain activity monitors – other** **electroencephalography(EEG)-based technologies** | | | | | | | | |
| Pu | 2021 | Australia | | MUSE 2 (InteraXon Inc., Toronto ON, Canada) | Pain | To assess the feasibility of a headband EEG to monitor pain in long-term care residents with dementia. | Quantitative cross-sectional study with 4 older adults with dementia and chronic pain in a long-term care facility. Each participant was monitored for 10 minutes. The Pain Assessment in Advanced Dementia (PAINAD) scale was administered before and after the measurement. | The device was acceptable to people with dementia and no participant tried to remove the device. No discomfort was reported. Appropriately positioning the device was challenging. |
| Vacas | 2016 | USA | | SedLine Brain Function Monitor (Masimo Corp., Irvine, California) | Sleep - stages | To validate the device in laboratory setting and to test the feasibility of its use in the ICU. | Quantitative cross-sectional study with three adult outpatients in the laboratory and 23 ICU patients. | The system is feasible in the ICU. |
| **Brain activity monitors –** **the concept of sedation monitors** | | | | | | | | |
| Six | 2020 | Belgium | | Not reported | Sedation | To investigate what influences professional caregivers and family members’ attitudes towards the use of monitors during continuous sedation until death. | Qualitative interview study with 20 professional caregivers and 15 family members of patients in a palliative care setting. | Family members and professional caregivers found the use of monitors during continuous sedation until death acceptable because this would be a more objective assessment of consciousness and pain, which gave reassurance to family and guides titration for the professional caregivers. |
| **Electrocardiography (ECG)** | | | | | | | | |
| Li | 2022 | China | | myBeat-WHS-1 (Union Tool Co., Ltd., Japan) | Stress | To use a wearable ECG monitor to measure workplace stress in nurses. | Quantitative cross-sectional study with 17 healthy nurses during one work day at the hospital. Participants wore the device and filled in the Chinese Nurses Stress Response Scale (CNSRS) after work. The feasibility was evaluated by the authors. | The authors concluded that it was feasible to use the portable device to monitor stress in nurses in their work environment. |
| Tao | 2021 | China, United Arab Emirates, Bahrain | | Not reported | Atrial fibrillation | To improve diagnostic efficiency of severe fever with thrombocytopenia syndrome bunyavirus (SFTSV) complicated by atrial fibrillation | Quantitative randomized controlled study with 200 older adults with SFTSV and hypertrophic cardiomyopathy in the hospital and 20 healthy adults. After surgery for cardiac sympathetic block, patients were randomly assigned to a monitoring group using ECG, supported by a new algorithm, or a routine observation group to monitor atrial fibrillation. Patients reported their satisfaction. | Patients were significantly more satisfied with the ECG atrial fibrillation monitoring technology than clinical observation (*p* < 0.05). |
| **EEG and ECG** | | | | | | | | |
| Tiihonen | 2008 | Finland | | Developed by the authors | Depth of sedation | To develop and validate a portable device to measure the depth of sedation with auditory event-related potentials (ERP). | Quantitative cross-sectional study with four healthy adults in the laboratory. Technical validation of the device was performed. ERP, EEG, ECG, and somatosensory-evoked potentials were measured. The quality of the signals were evaluated by an experienced neurophysiologist. | ERP and somatosensory-evoked potential measurements generated distinct responses. The quality of the signal recordings was good. The noise amplitudes were acceptable. The interface was easy to use. (Note: The study reported results of the technical validation, but no clinimetrics.) |
| **Electrodermal activity (EDA) monitors** | | | | | | | | |
| Leborgne | 2023 | Netherlands | | Developed by the author | Stress | To develop a sock garment to detect stress in people with intellectual disabilities. | Quantitative non-randomized experimental study. Sixty adults with severe intellectual disabilities in long-term care facilities tried on the garment. It was not clear whether they also participated in the experiment. The feasibility was evaluated by the researchers. | Of the 60 clients, 48 accepted and wore the sock. The others did not wear it due to other garments necessary for their comfort, such as compression socks, or due to repeated behavior resulting in removing the clothing. The researchers concluded that the sock was comfortable and user-friendly for most people with intellectual disabilities. |
| **Incontinence sensors** | | | | | | | | |
| Boerakker | 2022 | Netherlands | | Abena Nova (Abena Group, Denmark), WeSense (Seneca Sense Technologies, Canada) | Incontinence | To investigate the use of intelligent incontinence material in nursing homes. | Mixed-methods pilot study with 25 older adults in nursing homes with urinary incontinence. Abena Nova and WeSense were evaluated by the clients, family caregivers, and staff. | The clients did not feel the presence of extra sensors in the incontinence material. Family caregivers valued less dirty laundry and less unnecessary care moments. The staff reported that the technology is time-saving and they could trust it. The downsides were that the bowel incontinence is not measured and that the sensors are sometimes difficult to attach. |
| Nikoletti | 2004 | Australia | | Not reported | Incontinence | To evaluate an incontinence alarm in acute elderly care setting. | Mixed-method study with 41 older adults on acute care rehabilitation wards. Incontinence was monitored for 72 hours for each patient. | Numerous problems were reported: false alarms, false negatives, shorter transmission range than claimed by the manufacturer, the need to recharge frequently, and sensors being discarded with pads.  Resolving these issues was time-consuming and frustrating for staff and patients. There was resistance of experienced staff to learn the technology and to change personal routines. |
| Toba | 1996 | Japan | | Not reported | Incontinence | To evaluate the effects of wearing a thin-film urinary drainage sensor in the incontinence material on urinary quality of life and nursing work hours. | Mixed method cross-sectional study with 4 older adults with urinary incontinence in nursing homes. Participants were asked about the comfort of the sensors once a day for four days. Nursing staff filled in a survey about the burden. | Among the 16 responses from the older adults, 14 (87.5%) were "not at all uncomfortable," 2 (12.5%) were "it does not bother me". 3 out of 4 nursing staff commented that the interruption of other working activities led to reduced efficiency. They didn’t perceive the changing and cleaning of the incontinence materials as burdensome. |
| Van der Hurk | 1998 | Netherlands | | Developed by the authors | Incontinence | To evaluate the applicability and validity of the Urine Leakage Recording Device. | Quantitative non-randomized experimental study with 5 older adults. Participants were instructed to turn and void during the experiment. The experimenter evaluated the operation of the device. The setting was likely a laboratory, but not reported in the manuscript. | No restriction nor inconvenience was reported by the participants. The device was easy to operate. |
| **Multi-modal monitoring systems – polysomnography (PSG)** | | | | | | | | |
| Abdenbi | 2002 | France | | CID 102 and CID 108 (CIDELEC, Sainte-Gemmes-sur-Loire, France) | Sleep | To evaluate an ambulatory sleep monitoring set-up. | Quantitative cross-sectional study with 25 adults with possible sleep apnea. 15 had both 102 (respiratory recordings) and 108 (full polysomnographic recordings) at home for one night. 10 had CID 102 set up at the hospital and recorded at home. | Technical failures occurred in 2/15 with CID 108. No failure with CID 102. |
| Koskinen | 2021 | Finland | | Collare (Nukute Ltd, Finland) | Sleep - apnea | To validate the Nukute Collare in diagnosing sleep apnea. | Mixed-methods cross-sectional study with 71 adults with or without sleep apnea. Collare measurement was compared with standard PSG. Participants filled in a questionnaire to evaluate the device.* | 45 out of 71 measurements were completed valid. Most participants (93%) considered Collare easy to use, 85% reported that it did not disturb their sleep, 90% did not feel pain or tenderness in the neck after the measurement, 75% considered Nukute Collare more comfortable than the standard PSG, 85% said that they would use it again. |
| Morales | 2012 | USA | | ResCare AutoSet™ (ResMed, Sydney, Australia) | Sleep - apnea | To assess the feasibility of a two-stage screening procedure for obstructive sleep apnea syndrome (OSAS). | Quantitative cross-sectional study with 452 older adults in the community. Participants conducted an overnight home recording of nasal pressure on their own, kept a diary and received a separate, standard PSG. | Complete data on 442 of 452 (98%) home recordings |
| Silvia | 2014 | USA | | M1 (SleepImage, Denver, CO, USA) | Sleep - insomnia | To evaluate a brief sleep intervention and to investigate the feasibility and effectiveness of sleep monitoring of the M1 device. | Quantitative cross-sectional study with 8 adults with bipolar disorder. Participants wore the device for two seven-night periods at home and kept a sleep diary. They filled in a M1 acceptability questionnaire after each week of wearing the device. | The M1 device was evaluated as easy to use, easy to apply and did not interfere with the participants’ sleep. The device was worn for at least two nights by all participants and on average 6.12 and 5.50 days in each week. The willingness to wear the device again declined after the second week, but was still high. |
| Lazazzera | 2022 | France | UpNEA (developed by the authors) | | Sleep - apnea | To present a system aimed to predict respiratory and cardiovascular disorders. UpNEA was part of the whole system. | Quantitative cross-sectional study with one healthy adult. The participant wore the glove-life device for four nights and reported on its comfort.* | The device did not cause discomfort to the participant. |
| **Multi-modal monitoring systems – with environmental sensors** | | | | | | | | |
| Au-Yeung | 2020 | USA | | Ambient sensors: model not reported (NYCE Sensors, Vancouver, BC),  Pressure mats: model not reported (Emfit, Finland),  Actigraphy: Actiwatch Spectrums (Philips Respironics, Murrysville, PA),  Environmental sensors: Thunderboard Sense 2-SLTB004A (Silicon Labs, Austin, TX) | Agitation | To provide a proof of concept of monitoring and predicting agitation in memory care facilities with by monitoring the activity, sleep, and the environment (temperature, light, sound, humidity). | Quantitative case study with one adult with dementia in a memory care unit. The participant was continuously monitored by the system for 138 days. Agitation was recorded by nurses. The feasibility was evaluated by the researchers. | Ambient motion sensors functioned well with no technical problems. Sleep was successfully monitored for 91 out of 137 nights due to Wi-Fi problems or the participant not sleeping on the bed. Actigraphy was off-wrist 2% of the time. Environmental data was successfully collected 48% of the time. |
| Khan | 2019 | Canada | | Developed by the authors | Agitation | To develop a system to automatically detect agitation in people with dementia. The proposed system includes actigraphy, EDA monitor, pressure mat, video cameras, environmental motion sensors, and door sensors. | Quantitative cross-sectional study with two older adults with dementia at a rehabilitation institute. The participants were monitored for 15 and 13 days respectively. Agitation episodes were noted by nurses. Different algorithms were tested using different combinations of sensor data. The feasibility was evaluated by the authors. | The system was feasible to monitor people with dementia. |
| Davidoff | 2022 | Belgium, Netherlands | | Wearables: Chill+, LYS (LYS Technologies), Blooloc tracker;  Pressure mat: EMFIT QS (EMFIT, Finland);  Environmental sensors: Metatracker (MbientLab), micrphone: Nokia 6.1 Android phone, Blooloc | Anxiety | To pilot test a system that identifies physiological markers and contextual triggers of agitation in people with dementia. EDA, heart rate, activity, skin temperature, light, sleep, position of the participant, and environmental sound were monitored. | Quantitative cross-sectional study with older adults with dementia with severe behavioral symptoms in a neuropsychiatric ward. The number of participants was not reported. Agitation was scored by the nurse at nine random moments per day using the Pittsburgh Agitation Scale and the Richmond Agitation Sedation Scale. | Some participants took off the wearables when they were most agitated. Connectivity problems and some rooms not installing the devices complicated the data collection. Synchronizing all the data was intensive and errors lead to loss of data. |
| Davoudi | 2019 | USA | | Developed by the authors | Delirium | To pilot test a system that includes a camera, three actigraphy, a light sensor, and a microphone in the ICU. | Quantitative cross-sectional study with 17 older adults in the surgical ICU, nine of them had delirium. Participants were monitored continuously for a maximum of seven days. The feasibility was evaluated by the authors. | The system was feasible to monitor critically ill patients in the ICU. |
| Rose | 2015 | USA | | Tab-like bed sensor: Wireless Identification and Sensing Platform (WISP, brand not reported); Watch: Technology-Enabled Medical Precision Observation (TEMPO, brand not reported); Incontinence sensor: DryBuddy (brand not reported)  Microphone: model and brand not reported | Sleep, agitation, and urinary incontinence | To investigate the feasibility and acceptability of the wireless devices, including a tab-like bed sensor, a smart watch, an incontinence sensor, and a microphone, in community-dwelling people with dementia. | Quantitative cross-sectional study with 50 community-dwelling older adults with dementia. Participants were monitored for five to seven days. Family caregivers set up the devices with help from the study team via the phone and they received an interview about the feasibility after the study. | Minimal effort was required to operate the system, but it was still challenging for caregivers who were not very familiar with technology. Troubleshooting via the phone was challenging, but reviewing the data helped. Adding another bedside receiver solved problems with the incontinence sensors. Bowel incontinence complicated the measurement. |
| **Multi-modal monitoring systems – other multi-modal systems** | | | | | | | | |
| Iaboni | 2022 | Canada | | Empatica E4 (Empatica) | Agitation and aggression | To develop personalized machine learning models for detecting behavioral and psychological symptoms of dementia (BPSD). The device monitored motion, blood volume pulse, EDA, and skin temperature. | Quantitative cross-sectional study with 17 older adults with dementia and BPSD in a dementia care unit. Participants wore the Empatica wristband for eight weeks during waking hours. Agitation and aggression events were annotated by trained nurses. | The models could recognize the presence of motor agitation (median Area under the receiver-operating characteristic curves (AUC) = 90%), verbal aggression (median AUC = 86%), and physical aggression (median AUC = 82%) |
| Rajasekaran | 2011 | USA | | Portable Autonomous Multisensory Intervention Device (PAMID, developed by the authors) | Agitation | To test the reliability of the chest belt which integrated measurements for heart rate, skin temperature and EDA, and to evaluate its appearance and comfortability. | Quantitative non-randomized experimental study with six community-dwelling older adults in the laboratory. During a 40-minute experiment, participants took four sets of STROOP tests while being monitored by the device and conventional instruments. The appearance and comfort of the device were evaluated by the participants. | The appearance of the device was rated 2.7 out of 3, and the comfortability was rated 2.8 out of 3. |
| Miranda | 2016 | USA | | Heart rate: Hxm (Zephyr); EDA: Empatica E3; EEG: Muse band | Anxiety | To describe an experiment eliciting anxiety in informal caregivers for people with dementia, while measuring heart rate, EDA, and EEG. | Quantitative non-randomized experimental study with 10 healthy adults in the laboratory. During 30-min sessions, participants had a relaxation stage and performed therapy with a simulated person with dementia. The level of anxiety was recorded by the participants and researchers. Models were developed using the physiological parameters to predict the presence of anxiety. The feasibility was evaluated by the authors. | When participants moved during the sessions, EEG signal was poor. No reliability information was reported about the other sensors. |
| **Non-contact monitoring systems – pressure mats** | | | | | | | | |
| Sakai | 2009 | Japan | | KINOTEX sensor (NITTA Corp. Osaka, Japan) | Risk of pressure ulcers | To examine the feasibility of using continuous interface pressure monitoring to prevent pressure ulcers in the ICU. | Quantitative cross-sectional study with 30 postoperative patients in the ICU. They were monitored for up to 48 hours. | Continuous monitoring of the intensity and duration of whole-body interface pressure using the KINOTEX sensor is feasible in intensive care patients. |
| **Non-contact monitoring systems – multiple sensors** | | | | | | | | |
| Kroll | 2020 | Germany | | Camera: Mini-Webcam (Conrad Electronics SE, Hirschau, Germany);  Acoustic sensor: ME32 (Olympus Imaging Europa GmbH, Hamburg, Germany)  Pressure mat: SafeBed IP system (Emfit® Ltd., Vaajakoski, Finland) | Agitation | To validate the non-contact monitoring system and a tent-like shelter for people with dementia. | Quantitative cross-sectional study with six healthy adult volunteers in the laboratory and 19 patients, most of whom had dementia, in the emergency department and a geriatric-gerontopsychiatric ward. Participants were monitored for two hours, the first hour without the tent-like shelter, the second hour with it. | There were little perturbations during the measurements in the emergency department and geriatric-gerontopsychiatric ward. One out of 19 measurements did not take place because of non-cooperation of the patient. |
| *Notes:* Adults = mean age between 18 and 65; Older adults = mean age over 65; ICU = intensive care unit.  References can be found in Supplement IV.  *The setting was not reported. | | | | | | | | |
