# Supplementary material for: Noninvasive monitoring technologies to identify discomfort and distressing symptoms in persons with limited communication at the end of life: a scoping review
Source: BMC Palliat Care. 2024 Mar 21;23:78. doi: 10.1186/s12904-024-01371-0 (PMC10956214; doi:10.1186/s12904-024-01371-0)
Supplement: Supplementary file 3 — Additional file 3: eTable 3 Manuscripts in which the clinimetrics, acceptability or feasibility of the technologies that monitor distress and discomfort were not reported (the technology is either used as standard measurement or is newly developed). [file 12904_2024_1371_MOESM3_ESM.docx]

**Supplement III of “Noninvasive monitoring technologies to identify discomfort and distressing symptoms in persons with limited communication at the end of life: A scoping review”**

| eTable 3  *Manuscripts in which the clinimetrics, acceptability or feasibility of the technologies that monitor distress and discomfort were not reported (the technology is either used as standard measurement or is newly developed)* | | | | | |
| --- | --- | --- | --- | --- | --- |
| First author | Year | Country | Monitoring technology (model and brand) | Symptom monitored | Participant population and setting |
| **Actigraphy** | | | | | |
| Burnett-Zeigler | 2018 | USA | Not reported | Stress | Adults with depressive symptoms* |
| Godfrey | 2009 | UK, Ireland | activPAL Professional (PAL Technologies Ltd, Glasgow, UK) | Delirium (motor subtypes) | Adults receiving palliative care in hospice |
| Mahlberg | 2007 | Germany | Actiwatch (Cambridge Neurotechnology Ltd, Cambridge, UK) | Agitation | Older adults with Alzheimer’s disease from a geriatric psychiatry unit |
| Blytt | 2017 | Norway | Actiwatch Spectrum (Philips Respironics Inc, Murrysville, Pennsylvania) | Sleep | Older adults with or without dementia, living in nursing homes |
| Buratti | 2021 | Italy | Actiwatch Spectrum or Actiwatch-2 (Philips Respironics Inc, Murrysville, Pennsylvania) | Sleep | Older adults with Alzheimer’s disease, mild cognitive impairment, or no cognitive impairments living at home |
| Jaiswal | 2023 | USA | Actiwatch Spectrum Plus (Phillips Respironics Inc, Murrysville, Pennsylvania) | Sleep | Adults in the ICU, with or without delirium |
| Wilcox | 2019 | Canada | Actiwatch Spectrum Plus (Phillips Respironics, Bend, Oregon, USA) | Sleep | Adults in the hospital, after discharge from the ICU where they received at least 3 days of mechanical ventilation |
| Hoekert | 2006 | Netherlands | Actiwatch (Cambridge Neurotechnology Ltd., Cambridge, U.K.) | Sleep | Older adults with dementia living in group care facilities |
| Meadows | 2010 | UK | Actiwatch (Cambridge Neurotechnology Ltd, Cambridge, UK) | Sleep | Older adults both institutional care residents and community dwelling poor sleepers |
| Most | 2012 | Netherlands | Actiwatch (Cambridge Neurotechnology Ltd., Cambridge, UK) | Sleep | Older adults with Alzheimer’s disease and control group* |
| van Someren | 2019 | Netherlands and Italy | Actiwatch (Cambridge Neurotechnology Ltd, Cambridge, UK) | Sleep | Older adults* |
| de Feijter | 2021 | Netherlands | Actiwatch AW4 (Cambridge Neurotechnology Ltd, Cambridge, UK) | Sleep | Adults and older adults living at home |
| van den Berg | 2008 | Netherlands | Actiwatch AW4 (Cambridge Neurotechnology Ltd, Cambridge, UK) | Sleep | Older adults living at home |
| van den Berg | 2009 | Netherlands | Actiwatch AW4 (Cambridge Neurotechnology Ltd, Cambridge, UK) | Sleep | Older adults living at home |
| Kazui | 2017 | Japan | Actiwatch-L (Mini Mitter Co., Inc.-Respironics, Inc. Co. Bend, Oregon, USA) | Sleep | Older adults with Lewy Body dementia and control group* |
| Rowe | 2010 | USA | Actiwatch-L (Mini Mitter Co., Inc.-Respironics, Inc. Co. Bend, Oregon, USA) | Sleep | Adults who take care of their family members with dementia at home |
| Leary | 1998 | Ireland | Accelerometer (Gaewihler Electronics, Hombrechtikon, Switzerland) | Sleep | Adults: hypertensive patients and control group* |
| van Hilten | 1994 | Netherlands and Canada | Activity monitor (Gaehwiler Electronic, CH-8634, Hombrechtikon, Switzerland) | Sleep | Older adults with Parkinson’s disease and control group at an outpatient neurology department |
| Guarnieri | 2020 | Italy | Fitbit Flex (Fitbit Inc., San Francisco, California) | Sleep | Adults and older adults with Alzheimer’s disease or amnestic Mild Cognitive Impairment in a multicenter setting |
| Yesavage | 2020 | USA | Motionwatch (Ambulatory Monitoring Inc., Ardsley, New York) | Sleep | Older adults with Alzheimer’s disease* |
| Mulin | 2011 | France | Micro- Mini MotionLogger (Ambulatory-Monitoring, Inc., Ardsley, New York) | Sleep | Older adults with Alzheimer’s disease, with or without apathy* |
| Currie | 2003 | Canada | Mini-motion logger actigraph units (Ambulatory Monitoring Inc., Ardsley, New York) | Sleep | Adults: insomnia patients and control group* |
| Watanabe | 2002 | Japan | Mini Motion Logger (Ambulatory Monitoring Inc., Ardsley, New York) | Sleep | Older adults with or without hypertension living at home |
| Kotschet | 2023 | Australia | Parkinson's Kinetigraph (PKG, Global Kinetics Corporation^TM^, Australia) | Sleep | Adults with Huntington’s disease |
| Jones | 2020 | Australia | SenseWear Professional 8.0 (Temple Healthcare, BodyMedia, Inc.) | Sleep | Older adults with dementia and sleep problems* |
| Spira | 2017 | USA | SleepWatch-O (Ambulatory Monitoring Inc., Ardsley, New York) | Sleep | Older adults living at home |
| Yaffe | 2007 | USA | Sleepwatch-O (Ambulatory Monitoring Inc., Ardsley, New York) | Sleep | Older adults. Data was taken from a previous study about osteoporotic fractures* |
| Cabanel | 2020 | Germany | SOMNOwatch system (Somnomedics, Randersacker, Germany) | Sleep | Older adults: memory clinic patients |
| Westerberg | 2010 | USA | Not reported | Sleep | Older adults with amnestic Mild Cognitive Impairment and control group living at home |
| Elías | 2020 | USA | Not reported | Sleep | Older adults: ICU survivors |
| **Brain activity monitors – Bispectural Index (BIS)** | | | | | |
| Barbato | 2017 | Australia | Bispectral Index monitor (Covidien Pty Ltd.), with Quatro sensors. | Sedation and comfort | Adult patients at the end of life in palliative care units of the hospital |
| Bass | 2019 | USA | Not reported | Sedation and pain | Adults in the ICU |
| De Deyne^o^ | 1998 | Belgium | Aspect A-1000 EEG analyser (Aspect, Natick, USA) | Sedation | Adult patients with a Ramsay sedation score of 6 in the ICU |
| Monreal-Carrillo | 2017 | USA | BIS Vista Bilateral Monitoring System 1.2 (Aspect Medical Systems, Inc. Norwood, MA, USA) | Sedation | Adults on palliative sedation in the hospital |
| Quraishi | 2011 | USA | BIS (Aspect Medical Systems, Inc. Norwood, MA) | Sedation | Adult with locked-in syndrome in the ICU |
| **Brain activity monitors – other electroencephalography (EEG)-based technologies** | | | | | |
| Espie | 1998 | UK | Walter-Graphtex System and Oxford Medilog 9000-II ambulatory system | Sleep | Adults with severe or profound intellectual disability and epilepsy. Assessment was performed in the hospital and at home. |
| Sato | 2002 | USA, Japan | Not reported | Sleep | Older adults in the community. |
| **Electrocardiography (ECG)** | | | | | |
| Estrada | 2000 | USA | 78720 ASDN system (Hewlett Packard) | Arrhythmia | Adults in non–intensive-care telemetry unit |
| Gerber | 2019 | Switzerland | Carescape Monitor B650 (GE Healthcare, Little Chalfont, United Kingdom) | Stress | Critically ill adults in the ICU |
| Gerber | 2019 | Switzerland | Carescape Monitor B650 (GE Healthcare, Little Chalfont, United Kingdom) | Stress | Healthy adults. The experiment was conducted in the ICU environment. |
| Park | 2022 | Korea | Apple Watch Series 4, 5, or 6 smartwatch (Apple Inc) | Stress | Healthy nurses during cardiopulmonary resuscitation training |
| **EEG and ECG** | | | | | |
| Six | 2019 | Belgium | EEG: NeuroWave Systems Inc.  ECG: ANI (Mdoloris Medical Systems SAS) | Depth of sedation and pain | Older adult in the palliative care unit undergoing palliative sedation |
| Six | 2021 | Belgium | EEG: NeuroWave Systems Inc.  ECG: ANI (Mdoloris Medical Systems SAS) | Depth of sedation and pain | Older adults undergoing palliative sedation until death |
| **Electrodermal activity (EDA) monitors** | | | | | |
| Jusilla | 2018 | Finland | Moodmetric smart ring (Vigofere Oy, Finland) | Stress | No participants. The manuscript presented the design of a system |
| Massot | 2010 | France | EmoSense (developed by the authors, based on Programmable Systemon-Chip, Cypress MicroSystems, Inc.) | Stress | Healthy blind adults in an urban space |
| Melander | 2018 | Sweden, Norway | Discrete Tension Indicator (DTI-2) (  Philips Research); Empatica E4 (Empatica Incorporation) | Stress | Older adults with dementia in nursing homes |
| Quad Industries | 2022 | Netherlands | Model not reported, developed by Mentech (the Netherlands) and Quad Industries (Belgium) | Stress | No participants. The manuscript presented a printed electronic patch for people with severe intellectual disability. |
| **Incontinence sensors** | | | | | |
| Fischer | 2019 | Austria | Developed by the authors | Incontinence, occupancy of the bed | No participants. The manuscript reports the development of the electronic system. |
| Wai | 2008 | Singapore | Developed by the authors | Incontinence | No participants. The manuscript reports the design of the system. |
| **Multi-modal systems – Polysomnography (PSG)** | | | | | |
| Bell | 1996 | USA | Model 8-16 EEG machines (Grass Instruments) | Sleep | Older healthy adults at a sleep laboratory |
| Bugalho | 2019 | Portugal | XLTEK-TREX (Natus Medical Inc., Middleton, USA) | Sleep | Older adults with dementia, Parkinson Disease, or ideopathic rapid eye movement (REM) sleep behavior disorder at a sleep laboratory |
| Bugalho | 2019 | Portugal | XLTEK-TREX (Natus Medical Inc., Middleton, USA) | Sleep | Older adults with REM sleep behavior disorder at a sleep laboratory |
| Cooke | 2009 | USA | Embla recording system (Flaga Medical Devices/Medcare, Reykjavik, Iceland) | Sleep | Older adults with Alzheimer's disease and obstructive sleep apnea at home and at a sleep laboratory |
| Dew | 2003 | USA | Not reported | Sleep | Healthy older adults at a sleep laboratory |
| Dijkstra | 2019 | Belgium | BrainnetMorpheus (MEDATEC) | Sleep - Isolated REM sleep without atonia | Adults with sleep problems at a sleep laboratory |
| Djonlagic | 2019 | USA, Germany | Compumedics Siesta Portable PSG (Abottsville, Australia) | Sleep - stages | Older adults with or without cognitive impairment at home |
| Eisensehr | 2001 | Germany | A digital 32-channel system (Brainlab, Schwarzer, Munich, Germany) | Sleep - REM behavioral disorder | Older adults with Parkinson Disease and adults without Parkinson Disease at a sleep laboratory |
| Fanfulla | 2011 | Italy | N-S 7000 (Embla, Denver, CO, USA) | Sleep | Older adults in the step-down units after ICU discharge |
| Fleming | 2015 | USA | ExSpiron (Respiratory Motion, Inc. USA) | Hypopnea | Adult with obstructive sleep apnea in post-anesthesia care unit |
| Gelber | 2015 | USA | Not reported | Sleep - disordered breathing | Older adults without moderate or severe dementia at home |
| Gebran | 2009 | Canada | AR-B1831 single-board-computer (Accroser Technology Co. Ltd., Cypress, California) | Sleep – narcolepsy episodes | No participants. The manuscript introduces a prototype of the devices. |
| Herring | 2020 | USA | Not reported | Sleep - insomnia | Older adults with Alzheimer’s Disease and insomnia at a sleep laboratory |
| Heude | 1996 | France | Schwarzer ED24 (Madhaus, Germany) | Sleep | Adults with snoring and daytime somnolence at a laboratory |
| Jiang | 2013 | China, Italy | Polysmith 5.0 (Nihon Khoden, U.S.) | Sleep | Adults with VCIND (vascular cognitive impairment, no dementia), stroke or healthy controls at sleep laboratory |
| Joo | 2011 | South Korea | ApneaLink (ResMed, Australia) | Sleep - apnea | Older adults with acute cerebral infarction and a healthy control group at a hospital |
| Kim | 2017 | South Korea | Embla™ N7000 (Embla, Reykjavik, Iceland) | Sleep - disordered breathing and sleep disturbances | Older adults with or without asthma* |
| Kim | 2011 | South Korea | Embla S7000 (Medcare system, NY) | Sleep - apnea and hypopnea | Older adults with mild cognitive impairment and controls at a laboratory |
| Kardiol | 1999 | Germany | 4-channel recording system, brand not recorded | Sleep - apnea | Adults with acute myocardial infarction in the ICU |
| Koo | 2019 | USA | Not reported | Sleep - apnea | Older adults with acute stroke or transient ischemic attack in the hospital |
| Lankford | 2008 | Canada, USA | Not reported | Sleep | Adults and older adults with insomnia at a sleep laboratory |
| Low | 2012 | USA | Not reported | Sleep | Adults with insomnia, with or without Human Immunodeficiency Virus (HIV) at a sleep laboratory |
| McCall | 2006 | USA | Not reported | Sleep - insomnia | Adults with insomnia at a sleep laboratory |
| Maglavera | 2006 | Greece | SENSATION Medical System (developed by the authors) | Sleep | No participants. The manuscript presented a system and proposed its possible applications. |
| Martinez-Nicolas | 2021 | Spain | Thermochron iButton DS1921H (Maxim Integrated Products, Sunnyvale, CA), HOBO Pendant G Acceleration Data Logger UA- 004–64 actimeter (Onset Computer, Bourne, MA). Standard PSG: model and brand not reported | Sleep and circadian rhythm | Adults with or without sleep disordered breathing at home and in the hospital |
| Onen | 2008 | France | Embla digital portable recording system (Flaga hf., Reykjavik, Iceland) | Sleep - apnea | Older adults with possible sleep apnea at a sleep center |
| Pao | 2013 | USA | Not reported | Sleep | Older adults with Lewy body dementia at a hospital |
| Patout | 2019 | UK | ALICE 5 (Philips-Respironics, Murrysville, PA, USA) | Respiration | Adult patients with chronic obstructive pulmonary disease (COPD) – obstructive sleep apnea overlap* |
| Piano | 2017 | Italy | Not reported | Sleep | Adult patients with Huntington Disease at a center for movement disorders |
| Piano | 2015 | Italy | Not reported | Sleep | Adult patients with Huntington Disease at a laboratory |
| Pittsley | 2005 | USA | a modified Respitrace/Medilog portable recorder | Sleep - disordered breathing | Older adults at home |
| Prasad | 2016 | USA | Embletta^TM^ PDS system (Natus Neurology Inc., Middleton, WI) | Sleep - apnea | Older adults with obstructive sleep apnea at home |
| Smith | 2009 | Australia | E series (Compumedics, Melbourne, Vic., Australia) | Sleep - apnea | Adults with obstructive sleep apnea* |
| Soderstrom | 2004 | Sweden | Embla (Flaga hf Reykjavik, Iceland) | Sleep | Adults at home |
| Targa | 2020 | Spain | Embletta (Embla, Canada);  Sibelmed Exea Serie 5 (Sibel SAU, Spain);  Alice 6 LDx (Philips Respironics, USA);  ApneaLink (Resmed, Canada) | Sleep - apnea | Older adults with Alzheimer's disease* |
| Terzaghi | 2013 | Italy | SD LTM 32 BS (Micromed, Treviso, Italy) | Sleep | Older adults with Lewy body dementia or Parkinson’s Disease at a hospital |
| Várady | 2002 | Hungary | Not reported, algorism developed by the authors | Sleep - apnea | Adults* |
| Varri | 2001 | Finland, France | Not reported, scoring system developed by the authors | Sleep | No participants. The manuscript reports the development of an algorism. |
| Walsh | 2010 | USA, UK, Germany | Not reported | Sleep | Older adults with insomnia at a laboratory |
| Wang | 2019 | China | Embletta (Embla, Suffolk, UK)  BI9800 (Biomedical Instruments Co., Ltd., Osaka, Japan) | Sleep - apnea | Adults with or without obstructive sleep apnea at a hospital |
| Wilcock | 2008 | UK | Pulsox 3i (Minolta, USA) | Sleep - hypoxemia | Adults with advanced cancer in a specialist palliative care unit |
| Xun | 2019 | China | Model not reported (Weinmann, Germany) | Sleep – structure and hypoxia | Adults with obstructive sleep apnea monitored in the laboratory |
| Zhang | 2017 | China, Germany | Alice 5 Diagnostic Sleep System (Philips Healthcare, Andover, USA) | Sleep – apnea and structure | Adults with snoring, with or without stroke history at a hospital |
| Tateishi | 1994 | Japan | Fukuda SM28 (Fukuda, Tokyo, Japan) | Sleep - apnea | Adults with coronary artery problems monitored in their normal daily life |
| **Multi-modal systems – with environmental sensors** | | | | | |
| Hoehn-Saric | 2004 | USA | Developed by other researchers, monitoring EDA, heart rate, activity level and ambient temperature | Anxiety | Adults with panic disorder or general anxiety disorder and healthy controls in their own environment |
| **Multi-modal systems – other multi-model systems** | | | | | |
| Hassan | 2020 | Pakistan, Saudi Arabia, Korea | Developed by the authors, including sEMG, EEG, and ECG | Pain | No participants. The manuscript proposed a fog computer architecture for remote pain monitoring. |
| **Non-contact monitoring systems - pressure mats** | | | | | |
| Fukuda | 2022 | Japan | Nemuri Scan (Paramount Bed, Tokyo, Japan) | Sleep | Older adults with Lewy body dementia in nursing homes |
| Higami | 2019 | Japan | Nemuri Scan (Paramount Bed, Tokyo, Japan) | Sleep | Older adults with Alzheimer’s disease in nursing homes |
| Tong | 2015 | China | RS-611 (Beijing Xinxing Yangsheng Technology Co., Ltd.) | Sleep – apnea and structure | Adults and older adults with obstructive sleep apnea-hypopnea syndrome* |
| Peterson | 2013 | USA | Pressure mapping system (XSENSOR Technology Corporation, Calgary, Canada) | Risk of pressure ulcers | Adults in intensive care and intermediate care units |
| Sofronova | 2021 | Bulgaria | Developed by the authors | Risk of pressure ulcers | One healthy adult. The manuscript reported the development of a an e-textile mat. |
| *Note*: Adults = mean age between 18 and 65; Older adults = mean age over 65; ICU = intensive care unit  References can be found in Supplement IV.  * The setting was not reported.  ^o^ This study aimed to test the feasibility, but did not report about it in the results or discussions. | | | | | |
