# Supplementary material for: Noninvasive monitoring technologies to identify discomfort and distressing symptoms in persons with limited communication at the end of life: a scoping review
Source: BMC Palliat Care. 2024 Mar 21;23:78. doi: 10.1186/s12904-024-01371-0 (PMC10956214; doi:10.1186/s12904-024-01371-0)
Supplement: Supplementary file 4 — Additional file 4. References of included articles. [file 12904_2024_1371_MOESM4_ESM.docx]

**Supplement IV of “Noninvasive monitoring technologies to identify discomfort and distressing symptoms in persons with limited communication at the end of life: A scoping review”**

References of included articles

1. AB EHaH. [TENA Identifi TM is bewezen effectief] TENA Identifi TM is proved effective [2023] [Available from: https://tena-images.essity.com/images-c5/789/261789/original/essitynl9049-def-identifi-factsheet-nw.pdf.

2. Abdenbi F, Ahnaou A, Royant-Parola S, Nedelcoux H, Rouault S, Alfandary D, et al. Ambulatory sleep recording in a healthcare network: A feasibility study. Comptes Rendus - Biologies. 2002;325(4):401-5.

3. Acebo C, Watson RK, Bakos L, Thoman EB. Sleep and apnea in the elderly: reliability and validity of 24-hour recordings in the home. Sleep. 1991;14(1):56-64.

4. Alam R, Bankole A, Anderson M, Lach J. Multiple-Instance Learning for Sparse Behavior Modeling from Wearables: Toward Dementia-Related Agitation Prediction. Annu Int Conf IEEE Eng Med Biol Soc. 2019;2019:1330-3.

5. Alessi CA, Yoon EJ, Schnelle JF, Al-Samarrai NR, Cruise PA. A randomized trial of a combined physical activity and environmental intervention in nursing home residents: Do sleep and agitation improve? Journal of the American Geriatrics Society. 1999;47(7):784-91.

6. Altini M, Kinnunen H. The Promise of Sleep: A Multi-Sensor Approach for Accurate Sleep Stage Detection Using the Oura Ring. Sensors (Basel). 2021;21(13).

7. Alvarez RF, Rabec C, Rubinos Cuadrado G, Cascon Hernandez JA, Rodriguez P, Georges M, et al. Monitoring Noninvasive Ventilation in Patients with Obesity Hypoventilation Syndrome: Comparison between Ventilator Built-in Software and Respiratory Polygraphy. Respiration. 2017;93(3):162-9.

8. Anusha G, Sujatha V, Swarnalatha M, Hema B, Lakshmi Devi M. An advanced Nursing homes activity tracking for Elderly Care support. Journal of Cardiovascular Disease Research (Journal of Cardiovascular Disease Research). 2021;12(3):3319-23.

9. Arbour C, Gélinas C, Loiselle CG, Bourgault P. An exploratory study of the bilateral bispectral index for pain detection in traumatic-brain-injured patients with altered level of consciousness. J Neurosci Nurs. 2015;47(3):166-77.

10. Arbour RB, Dissin J. Predictive value of the bispectral index for burst suppression on diagnostic electroencephalogram during drug-induced coma. The Journal of neuroscience nursing : journal of the American Association of Neuroscience Nurses. 2015;47(2):113-22.

11. Arnal PJ, Thorey V, Debellemaniere E, Ballard ME, Bou Hernandez A, Guillot A, et al. The Dreem Headband compared to polysomnography for electroencephalographic signal acquisition and sleep staging. Sleep. 2020;43(11).

12. Aslanidis T, Grosomanidis V, Karakoulas K, Chatzisotiriou A. Electrodermal Activity Monitoring During Painful Stimulation in Sedated Adult Intensive Care Unit Patients: a Pilot Study. Acta Medica (Hradec Kralove). 2018;61(2):47-52.

13. Au-Yeung WTM, Miller L, Beattie Z, Dodge HH, Reynolds C, Vahia I, et al. Sensing a problem: Proof of concept for characterizing and predicting agitation. Alzheimers Dement-Transl Res Clin Interv. 2020;6(1):10.

14. Bankole A, Anderson M, Smith-Jackson T, Knight A, Oh K, Brantley J, et al. Validation of noninvasive body sensor network technology in the detection of agitation in dementia. Am J Alzheimers Dis Other Demen. 2012;27(5):346-54.

15. Bankole A, Anderson MS, Homdee N, Alam R, Lofton A, Fyffe N, et al. BESI: Behavioral and Environmental Sensing and Intervention for Dementia Caregiver Empowerment-Phases 1 and 2. Am J Alzheimers Dis Other Demen. 2020;35:1533317520906686.

16. Barbato M, Barclay G, Potter J, Yeo W, Chung J. Correlation Between Observational Scales of Sedation and Comfort and Bispectral Index Scores. J Pain Symptom Manage. 2017;54(2):186-93.

17. Bass S, Vance ML, Reddy A, Bauer SR, Roach E, Torbic H, et al. Bispectral Index for Titrating Sedation in ARDS Patients During Neuromuscular Blockade. Am J Crit Care. 2019;28(5):377-84.

18. Becouze P, Hann CE, Chase JG, Shaw GM. Measuring facial grimacing for quantifying patient agitation in critical care. Comput Methods Programs Biomed. 2007;87(2):138-47.

19. Bell IR, Bootzin RR, Ritenbaugh C, Wyatt JK, DeGiovanni G, Kulinovich T, et al. A polysomnographic study of sleep disturbance in community elderly with self-reported environmental chemical odor intolerance. Biol Psychiatry. 1996;40(2):123-33.

20. Blytt KM, Bjorvatn B, Husebo B, Flo E. Clinically significant discrepancies between sleep problems assessed by standard clinical tools and actigraphy. BMC Geriatr. 2017;17(1):253.

21. Boerakker R. Project Eind Rapport (PER) - Pilot slim incontinentiemateriaal. Beneden-Leeuwen, Netherlands: Zorggroep Maas en Waal; 2022 21 February 2022.

22. Boissy P, Genest J, Patenaude J, Poirier MS, Chenel V, Beland JP, et al. Carbon nanotubes (CNTs) based strain sensors for a wearable monitoring and biofeedback system for pressure ulcer prevention and rehabilitation. Conference proceedings : . 2011;Annual International Conference of the IEEE Engineering in Medicine and Biology Society. IEEE Engineering in Medicine and Biology Society. Conference.:5824-7.

23. Brooks IJO, Friedman L, Bliwise DL, Yesavage JA. Use of the wrist actigraph to study insomnia in older adults. Sleep. 1993;16(2):151-5.

24. Bugalho P, Salavisa M. Factors Influencing the Presentation of REM Sleep Behavior Disorder: The Relative Importance of Sex, Associated Neurological Disorder, and Context of Referral to Polysomnography. J Clin Sleep Med. 2019;15(12):1789-98.

25. Bugalho P, Salavisa M, Marto JP, Borbinha C, Alves L. Polysomnographic data in Dementia with Lewy Bodies: correlation with clinical symptoms and comparison with other α-synucleinopathies. Sleep Med. 2019;55:62-8.

26. Buratti L, Camilletti R, Pulcini A, Rocchi C, Viticchi G, Falsetti L, et al. Sleep actigraphic patterns and cognitive status. J Integr Neurosci. 2021;20(2):385-92.

27. Burnett-Zeigler IE, Waldron EM, Hong S, Yang A, Wisner KL, Ciolino JD. Accessibility and feasibility of using technology to support mindfulness practice, reduce stress and promote long term mental health. Complementary therapies in clinical practice. 2018;33:93-9.

28. Buyse B, Borzee P, Kalkanis A, Testelmans D. In search of a cut-off apnea-hypopnea index in type 3 home portable monitors to diagnose and treat obstructive sleep apnea: a mathematical simulation. Journal of Sleep Research. 2023;32(1) (no pagination).

29. Cabanel N, Speier C, Müller MJ, Kundermann B. Actigraphic, but not subjective, sleep measures are associated with cognitive impairment in memory clinic patients. Psychogeriatrics. 2020;20(2):133-9.

30. Carlson CR, Wynn KT, Edwards J, Okeson JP, Nitz AJ, Workman DE, et al. Ambulatory electromyogram activity in the upper trapezius region: Patients with muscle pain vs. pain-free control subjects. Spine. 1996;21(5):595-9.

31. Casaccia S, Braccili E, Scalise L, Revel GM. Experimental Assessment of Sleep-Related Parameters by Passive Infrared Sensors: Measurement Setup, Feature Extraction, and Uncertainty Analysis. Sensors. 2019;19(17).

32. Castillo LI, Browne ME, Hadjistavropoulos T, Prkachin KM, Goubran R. Automated vs. manual pain coding and heart rate estimations based on videos of older adults with and without dementia. J Rehabil Assist Technol Eng. 2020;7:2055668320950196.

33. Chang Y, Xu L, Han F, Keenan BT, Kneeland-Szanto E, Zhang R, et al. Validation of the Nox-T3 portable monitor for diagnosis of obstructive sleep apnea in patients with chronic obstructive pulmonary disease. Journal of Clinical Sleep Medicine. 2019;15(4):587-96.

34. Chen J, Yan M, Chin Howe RL, Tong NW, Chee CS, Niu W, et al. BiovitalsTM: A Personalized Multivariate Physiology Analytics Using Continuous Mobile Biosensors. Annual International Conference of the IEEE Engineering in Medicine and Biology Society. 2019;IEEE Engineering in Medicine and Biology Society. Annual International Conference. 2019:3243-8.

35. Chikhaoui B, Ye B, Mihailidis A, editors. Ensemble learning-based algorithms for aggressive and agitated behavior recognition. Ubiquitous Computing and Ambient Intelligence: 10th International Conference, UCAmI 2016, San Bartolomé de Tirajana, Gran Canaria, Spain, November 29–December 2, 2016, Part II 10; 2016: Springer.

36. Choi J, Ahmed B, Gutierrez-Osuna R. Development and evaluation of an ambulatory stress monitor based on wearable sensors. IEEE transactions on information technology in biomedicine : a publication of the IEEE Engineering in Medicine and Biology Society. 2012;16(2):279-86.

37. Cicceri G, De Vita F, Bruneo D, Merlino G, Puliafito A. A deep learning approach for pressure ulcer prevention using wearable computing. Human-centric Comput Inf Sci. 2020;10(1):21.

38. Cooke JR, Ancoli-Israel S, Liu L, Loredo JS, Natarajan L, Palmer BS, et al. Continuous positive airway pressure deepens sleep in patients with Alzheimer's disease and obstructive sleep apnea. Sleep Med. 2009;10(10):1101-6.

39. Cooray N, Andreotti F, Lo C, Symmonds M, Hu MTM, De Vos M. Proof of concept: Screening for REM sleep behaviour disorder with a minimal set of sensors. Clin Neurophysiol. 2021;132(4):904-13.

40. Currie SR, Clark S, Rimac S, Malhotra S. Comprehensive assessment of insomnia in recovering alcoholics using daily sleep diaries and ambulatory monitoring. Alcoholism: Clinical and Experimental Research. 2003;27(8):1262-9.

41. Cusick G, Birkett A, Clarke-O'Neill S, Fader M, Cottenden AM. A system for logging incontinence events using a simple disposable sensor. Proceedings of the Institution of Mechanical Engineers. 2003;Part H, Journal of engineering in medicine. 217(4):305-10.

42. Dahaba AA, Xue JX, Xu GX, Liu QH, Metzler H. Bilateral Bispectral Index (BIS)-Vista as a measure of physiologic sleep in sleep-deprived anesthesiologists. Minerva Anestesiol. 2011;77(4):388-93.

43. Davidoff H, van den Bulcke L, Vandenbulcke M, De Vos M, van den Stock J, Van Helleputte N, et al. Toward Quantification of Agitation in People With Dementia Using Multimodal Sensing. Innovation in Aging. 2022;6(7):9.

44. Davoudi A, Malhotra KR, Shickel B, Siegel S, Williams S, Ruppert M, et al. Intelligent ICU for Autonomous Patient Monitoring Using Pervasive Sensing and Deep Learning. Sci Rep. 2019;9(1):8020.

45. De Deyne C, Struys M, Decruyenaere J, Creupelandt J, Hoste E, Colardyn F. Use of continuous bispectral EEG monitoring to assess depth of sedation in ICU patients. Intensive Care Med. 1998;24(12):1294-8.

46. de Feijter M, O'Connor MF, Arizmendi BJ, Ikram MA, Luik AI. The longitudinal association of actigraphy-estimated sleep with grief in middle-aged and elderly persons. J Psychiatr Res. 2021;137:66-72.

47. De jonckheere J, Dassonneville A, Flocteil M, Delecroix M, Seoane G, Jeanne M, et al. Ambulatory pain evaluation based on heart rate variability analysis: Application to physical therapy. Annu Int Conf IEEE Eng Med Biol Soc. 2014;2014:5502-5.

48. de Vries S, Smits R, Tataj M, Ronckers M, van der Pol M, van Oost F, et al. Accurate Stress Detection from Novel Real-Time Electrodermal Activity Signals and Multi-Task Learning Models. Cognitive Computing and Internet of Things. 2022;43:111-7.

49. Delaney L, Litton E, Melehan K, Huang H-C, Lopez V, Van Haren F. The feasibility and reliability of actigraphy to monitor sleep in intensive care patients: an observational study. Critical Care. 2021;25(1):1-12.

50. Dew MA, Hoch CC, Buysse DJ, Monk TH, Begley AE, Houck PR, et al. Healthy older adults' sleep predicts all-cause mortality at 4 to 19 years of follow-up. Psychosom Med. 2003;65(1):63-73.

51. Dietz-Terjung S, Geldmacher J, Brato S, Linker CM, Welsner M, Schobel C, et al. A novel minimal-contact biomotion method for long-term respiratory rate monitoring. Sleep and Breathing. 2020.

52. Dijkstra F, Viaene M, Crosiers D, De Volder I, Cras P. Frequency and characteristic features of REM sleep without atonia. Clin Neurophysiol. 2019;130(10):1825-32.

53. Dimitrievski A, Zdravevski E, Lameski P, Villasana MV, Pires IM, Garcia NM, et al. Towards Detecting Pneumonia Progression in COVID-19 Patients by Monitoring Sleep Disturbance Using Data Streams of Non-Invasive Sensor Networks. Sensors. 2021;21(9):14.

54. Djonlagic I, Aeschbach D, Harrison SL, Dean D, Yaffe K, Ancoli-Israel S, et al. Associations between quantitative sleep EEG and subsequent cognitive decline in older women. J Sleep Res. 2019;28(3):e12666.

55. Eisensehr I, v Lindeiner H, Jäger M, Noachtar S. REM sleep behavior disorder in sleep-disordered patients with versus without Parkinson's disease: is there a need for polysomnography? J Neurol Sci. 2001;186(1-2):7-11.

56. EJ VANS. Improving actigraphic sleep estimates in insomnia and dementia: how many nights? J Sleep Res. 2007;16(3):269-75.

57. Elías MN, Munro CL, Liang Z. Sleep Quality Associated With Motor Function Among Older Adult Survivors of Critical Illness. Nurs Res. 2020;69(4):322-8.

58. Espie CA, Paul A, McFie J, Amos P, Hamilton D, McColl JH, et al. Sleep studies of adults with severe or profound mental retardation and epilepsy. Am J Ment Retard. 1998;103(1):47-59.

59. Estrada CA, Rosman HS, Prasad NK, Battilana G, Alexander M, Held AC, et al. Evaluation of guidelines for the use of telemetry in the non-intensive-care setting. J Gen Intern Med. 2000;15(1):51-5.

60. Fanfulla F, Ceriana P, Lupo ND, Trentin R, Frigerio F, Nava S. Sleep Disturbances in Patients Admitted to a Step-Down Unit After ICU Discharge: the Role of Mechanical Ventilation. Sleep. 2011;34(3):355-62.

61. Favela J, Cruz-Sandoval D, Morales-Tellez A, Lopez-Nava IH. Monitoring behavioral symptoms of dementia using activity trackers. Journal of Biomedical Informatics. 2020;109:103520.

62. Fischer M, Renzler M, Ussmueller T. Development of a Smart Bed Insert for Detection of Incontinence and Occupation in Elder Care. IEEE Access. 2019;7:118498-508.

63. Fleming E, Voscopoulos C, George E. Non-invasive respiratory volume monitoring identifies opioid-induced respiratory depression in an orthopedic surgery patient with diagnosed obstructive sleep apnea: A case report. Journal of Medical Case Reports. 2015;9(1).

64. Fukuda C, Higami Y, Shigenobu K, Kanemoto H, Yamakawa M. Using a Non-Wearable Actigraphy in Nursing Care for Dementia With Lewy Bodies. Am J Alzheimers Dis Other Demen. 2022;37:15333175221082747.

65. Gabran SI, Moussa WW, Salama MA, George C. Portable real-time support-vector-machine-based automated diagnosis and detection device of narcolepsy episodes. Conference proceedings : . 2009;Annual International Conference of the IEEE Engineering in Medicine and Biology Society. IEEE Engineering in Medicine and Biology Society. Conference. 2009:903-6.

66. Gambrell M. Using the BIS monitor in palliative care: a case study. J Neurosci Nurs. 2005;37(3):140-3.

67. Ganglberger W, Bucklin AA, Tesh RA, Da Silva Cardoso M, Sun H, Leone MJ, et al. Sleep apnea and respiratory anomaly detection from a wearable band and oxygen saturation. Sleep Breath. 2022;26(3):1033-44.

68. Gelber RP, Redline S, Ross GW, Petrovitch H, Sonnen JA, Zarow C, et al. Associations of brain lesions at autopsy with polysomnography features before death. Neurology. 2015;84(3):296-303.

69. Gelinas C, Shahiri TS, Richard-Lalonde M, Laporta D, Morin JF, Boitor M, et al. Exploration of a Multi-Parameter Technology for Pain Assessment in Postoperative Patients After Cardiac Surgery in the Intensive Care Unit: The Nociception Level Index (NOL)TM. Journal of Pain Research. 2021;14:3723-31.

70. Gerber SM, Jeitziner M-M, Knobel SE, Mosimann UP, Müri RM, Jakob SM, et al. Perception and performance on a virtual reality cognitive stimulation for use in the intensive care unit: a non-randomized trial in critically ill patients. Frontiers in medicine. 2019:287.

71. Gerber SM, Jeitziner M-M, Sänger SD, Knobel SE, Marchal-Crespo L, Müri RM, et al. Comparing the relaxing effects of different virtual reality environments in the intensive care unit: observational study. JMIR perioperative medicine. 2019;2(2):e15579.

72. Ghorbani S, Golkashani HA, Chee N, Teo TB, Dicom AR, Yilmaz G, et al. Multi-Night at-Home Evaluation of Improved Sleep Detection and Classification with a Memory-Enhanced Consumer Sleep Tracker. Nat Sci Sleep. 2022;14:645-60.

73. Gibson RH, Gander PH. Monitoring the sleep patterns of people with dementia and their family carers in the community. Australas J Ageing. 2019;38(1):47-51.

74. Giménez S, Romero S, Alonso JF, Mañanas M, Pujol A, Baxarias P, et al. Monitoring sleep depth: analysis of bispectral index (BIS) based on polysomnographic recordings and sleep deprivation. J Clin Monit Comput. 2017;31(1):103-10.

75. Godfrey A, Conway R, Leonard M, Meagher D, Olaighin G. A classification system for delirium subtyping with the use of a commercial mobility monitor. Gait Posture. 2009;30(2):245-52.

76. Grap MJ, Hamilton VA, McNallen A, Ketchum JM, Best AM, Arief NY, et al. Actigraphy: analyzing patient movement. Heart Lung. 2011;40(3):e52-9.

77. Grasso I, Haigney M, Mortara D, Collen JF, Hostler J, Moores A, et al. Detection of sleep-disordered breathing with ambulatory Holter monitoring. Sleep and Breathing. 2018;22(4):1021-8.

78. Guarnieri B, Maestri M, Cucchiara F, Lo Gerfo A, Schirru A, Arnaldi D, et al. Multicenter Study on Sleep and Circadian Alterations as Objective Markers of Mild Cognitive Impairment and Alzheimer's Disease Reveals Sex Differences. Journal of Alzheimer's Disease. 2020;78(4):1707-19.

79. Haenggi M, Ypparila H, Takala J, Korhonen I, Luginbuhl M, Petersen-Felix S, et al. Measuring depth of sedation with auditory evoked potentials during controlled infusion of propofol and remifentanil in healthy volunteers. Anesthesia and Analgesia. 2004;99(6):1728-36.

80. Hassan SR, Ahmad I, Ahmad S, Alfaify A, Shafiq M. Remote Pain Monitoring Using Fog Computing for e-Healthcare: An Efficient Architecture. Sensors. 2020;20(22):21.

81. Herring WJ, Ceesay P, Snyder E, Bliwise D, Budd K, Hutzelmann J, et al. Polysomnographic assessment of suvorexant in patients with probable Alzheimer's disease dementia and insomnia: a randomized trial. Alzheimers Dement. 2020;16(3):541-51.

82. Heude E, Bourgin P, Feigel P, Escourrou P. Ambulatory monitoring of blood pressure disturbs sleep and raises systolic pressure at night in patients suspected of suffering from sleep-disordered breathing. Clinical Science. 1996;91(1):45-50.

83. Higami Y, Yamakawa M, Shigenobu K, Kamide K, Makimoto K. High frequency of getting out of bed in patients with Alzheimer's disease monitored by non-wearable actigraphy. Geriatr Gerontol Int. 2019;19(2):130-4.

84. Hirvonen K, Hasan J, Hakkinen V, Varri A, Loula P. The detection of drowsiness and sleep onset periods from ambulatory recorded polygraphic data. Electroencephalography and Clinical Neurophysiology. 1997;102(2):132-7.

85. Hoehn-Saric R, McLeod DR, Funderburk F, Kowalski P. Somatic symptoms and physiologic responses in generalized anxiety disorder and panic disorder: An ambulatory monitor study. Archives of General Psychiatry. 2004;61(9):913-21.

86. Hoekert M, der Lek RF, Swaab DF, Kaufer D, Van Someren EJ. Comparison between informant-observed and actigraphic assessments of sleep-wake rhythm disturbances in demented residents of homes for the elderly. Am J Geriatr Psychiatry. 2006;14(2):104-11.

87. Hsu C-Y, Ahuja A, Yue S, Hristov R, Kabelac Z, Katabi D. Zero-effort in-home sleep and insomnia monitoring using radio signals. Proceedings of the ACM on Interactive, mobile, wearable and ubiquitous technologies. 2017;1(3):1-18.

88. Iaboni A, Spasojevic S, Newman K, Schindel Martin L, Wang A, Ye B, et al. Wearable multimodal sensors for the detection of behavioral and psychological symptoms of dementia using personalized machine learning models. Alzheimer's and Dementia: Diagnosis, Assessment and Disease Monitoring. 2022;14(1) (no pagination).

89. Industries Q. Printed electronics for the health care sector: a real-life business case. Organic and Printed Electronics - OPE Journal. 2022:8-9.

90. Jaiswal SJ, Bagsic SRS, Takata E, Kamdar BB, Ancoli-Israel S, Owens RL. Actigraphy-based sleep and activity measurements in intensive care unit patients randomized to ramelteon or placebo for delirium prevention. Sci Rep. 2023;13(1):1450.

91. Jiang B, Ding C, Yao G, Yao C, Zhang Y, Ge J, et al. Polysomnographic abnormalities in patients with vascular cognitive impairment-no dementia. Sleep Med. 2013;14(11):1071-5.

92. Jiang M, Mieronkoski R, Syrjälä E, Anzanpour A, Terävä V, Rahmani AM, et al. Acute pain intensity monitoring with the classification of multiple physiological parameters. J Clin Monit Comput. 2019;33(3):493-507.

93. Jones C, Moyle W. A feasibility study of Dreampad (TM) on sleep, wandering and agitated behaviors in people living with dementia. Geriatr Nurs. 2020;41(6):782-9.

94. Joo BE, Seok HY, Yu SW, Kim BJ, Park KW, Lee DH, et al. Prevalence of sleep-disordered breathing in acute ischemic stroke as determined using a portable sleep apnea monitoring device in Korean subjects. Sleep and Breathing. 2011;15(1):77-82.

95. Jungquist CR, Chandola V, Spulecki C, Nguyen KV, Crescenzi P, Tekeste D, et al. Identifying Patients Experiencing Opioid-Induced Respiratory Depression During Recovery From Anesthesia: The Application of Electronic Monitoring Devices. Worldviews on Evidence-Based Nursing. 2019;16(3):186-94.

96. Jussila J, Venho N, Salonius H, Moilanen J, Liukkonen J, Rinnetmäki M, editors. Towards ecosystem for research and development of electrodermal activity applications. Proceedings of the 22nd International Academic Mindtrek Conference; 2018.

97. Kang D, Ye JY, Zheng L, Zhang JB, Bian QL. [Evaluation of Watch PAT as a diagnosing test for patients with obstructive sleep apnea hypopnea syndrome]. [Chinese]. Zhonghua er bi yan hou tou jing wai ke za zhi = Chinese journal of otorhinolaryngology head and neck surgery. 2012;47(10):813-6.

98. Kazui H, Adachi H, Kanemoto H, Yoshiyama K, Wada T, Tokumasu Nomura K, et al. Effects of donepezil on sleep disturbances in patients with dementia with Lewy bodies: An open-label study with actigraphy. Psychiatry Res. 2017;251:312-8.

99. Khan SS, Spasojevic S, Nogas J, Ye B, Mihailidis A, Iaboni A, et al. Agitation Detection in People Living with Dementia using Multimodal Sensors. Annu Int Conf IEEE Eng Med Biol Soc. 2019;2019:3588-91.

100. Kikhia B, Stavropoulos TG, Andreadis S, Karvonen N, Kompatsiaris I, Sävenstedt S, et al. Utilizing a Wristband Sensor to Measure the Stress Level for People with Dementia. Sensors (Basel). 2016;16(12).

101. Kim SH, Won HK, Moon SD, Kim BK, Chang YS, Kim KW, et al. Impact of self-reported symptoms of allergic rhinitis and asthma on sleep disordered breathing and sleep disturbances in the elderly with polysomnography study. PLoS One. 2017;12(2):e0173075.

102. Kim SJ, Lee JH, Lee DY, Jhoo JH, Woo JI. Neurocognitive dysfunction associated with sleep quality and sleep apnea in patients with mild cognitive impairment. Am J Geriatr Psychiatry. 2011;19(4):374-81.

103. Kinnunen H, Rantanen A, Kentta T, Koskimaki H. Feasible assessment of recovery and cardiovascular health: accuracy of nocturnal HR and HRV assessed via ring PPG in comparison to medical grade ECG. Physiol Meas. 2020;41(4).

104. Knuff A, Leung RH, Seitz DP, Pallaveshi L, Burhan AM. Use of Actigraphy to Measure Symptoms of Agitation in Dementia. Am J Geriatr Psychiatry. 2019;27(8):865-9.

105. Kobayashi M, Namba K, Tsuiki S, Nakamura M, Hayashi M, Mieno Y, et al. Validity of sheet-type portable monitoring device for screening obstructive sleep apnea syndrome. Sleep and Breathing. 2013;17(2):589-95.

106. Koehler U, Trautmann M, Trautmann R, Tabarelli O, Cassel W, Heitmann J, et al. [Does sleep apnea increase the risk of myocardial infarct during sleep?]. Z Kardiol. 1999;88(6):410-7.

107. Koley BL, Dey D. Real-time adaptive apnea and hypopnea event detection methodology for portable sleep apnea monitoring devices. IEEE Transactions on Biomedical Engineering. 2013;60(12):3354-63.

108. Koo BB, Sico JJ, Myers LJ, Perkins AJ, Levine D, Miech EJ, et al. Polysomnography Utilization in Veterans Presenting Acutely with Ischemic Stroke or Transient Ischemic Attack. Cerebrovasc Dis. 2019;48(3-6):179-83.

109. Koskinen K, Hannila E, Kallio M, Huuskonen U, Himanen S-L. CLINICAL STUDY: EVALUATING THE USABILITY AND CLINICAL PERFORMANCE OF THE NUKUTE COLLARE SYSTEM. Oulu Finland2021. p. https://nukute.com/products/clinical-validation

110. Kotschet K, Osborn S, Horne M. Measurement of bradykinesia and chorea in Huntington's Disease using ambulatory monitoring. Clinical Parkinsonism and Related Disorders. 2023;8 (no pagination).

111. Kroll L, Bohning N, Mussigbrodt H, Stahl M, Halkin P, Liehr B, et al. Non-contact monitoring of agitation and use of a sheltering device in patients with dementia in emergency departments: a feasibility study. Bmc Psychiatry. 2020;20(1):8.

112. Lachenmeier W, Lachenmeier DW. Home Monitoring of Oxygen Saturation Using a Low-Cost Wearable Device with Haptic Feedback to Improve Sleep Quality in a Lung Cancer Patient: A Case Report. Geriatrics (Switzerland). 2022;7(2).

113. Lai Kwan C, Mahdid Y, Motta Ochoa R, Lee K, Park M, Blain-Moraes S. Wearable Technology for Detecting Significant Moments in Individuals with Dementia. Biomed Res Int. 2019:1-13.

114. Lankford DA, Corser BC, Zheng YP, Li Z, Snavely DB, Lines CR, et al. Effect of gaboxadol on sleep in adult and elderly patients with primary insomnia: results from two randomized, placebo-controlled, 30-night polysomnography studies. Sleep. 2008;31(10):1359-70.

115. Lazazzera R, Carrault G. MonEco: a Novel Health Monitoring Ecosystem to Predict Respiratory and Cardiovascular Disorders. Irbm. 2022.

116. Leary AC, Murphy MB. Sleep disturbance during ambulatory blood pressure monitoring of hypertensive patients. Blood Pressure Monitoring. 1998;3(1):11-5.

117. Leblanc RG, Czarnecki P, Howard J, Jacelon CS, Marquard J. Usability Experience of a Personal Sleep Monitoring Device to Self-manage Sleep Among Persons 65 Years or Older With Self-reported Sleep Disturbances. CIN - Computers Informatics Nursing. 2022;40(9):598-605.

118. Leborgne F, Smits R, Gencheva M, De Vries S, Meinders E, Cluitmans P, et al. The development of a washable and durable smart textile to measure electrodermal activity for early stress recognition. Intelligent Human Systems Integration (IHSI 2023): Integrating People and Intelligent Systems. 2023;69(69).

119. Lee SB, Kwon JW, Sung S, Moon SH, Lee BH. Delirium after Spinal Surgery: A Pilot Study of Electroencephalography Signals from a Wearable Device. Appl Sci-Basel. 2022;12(19):10.

120. Li S, Xu L, Dong X, Zhang X, Keenan BT, Han F, et al. Home sleep apnea testing of adults with chronic heart failure. Journal of Clinical Sleep Medicine. 2021;17(7):1453-63.

121. Li X, Zhu W, Sui X, Zhang A, Chi L, Lv L. Assessing Workplace Stress Among Nurses Using Heart Rate Variability Analysis With Wearable ECG Device-A Pilot Study. Front Public Health. 2021;9:810577.

122. Low Y, Goforth HW, Omonuwa T, Preud'homme X, Edinger J, Krystal A. Comparison of polysomnographic data in age-, sex- and Axis I psychiatric diagnosis matched HIV-seropositive and HIV-seronegative insomnia patients. Clin Neurophysiol. 2012;123(12):2402-5.

123. Luo A, Muraida S, Pinchotti D, Richardson E, Ye E, Hollingsworth B, et al. Bispectral Index Monitoring With Density Spectral Array for Delirium Detection. Psychosomatics. 2020.

124. Maglavera S, Maglaveras N, Lekka I, Bekiaris A, Penzel T, Canisius S, et al. SENSATION remote monitoring system for enabling the "anytime, anywhere" monitoring of patients with selected sleep disorders. Conference proceedings : . 2006;Annual International Conference of the IEEE Engineering in Medicine and Biology Society. IEEE Engineering in Medicine and Biology Society. Conference. 1:3869-72.

125. Mahlberg R, Walther S. Actigraphy in agitated patients with dementia. Monitoring treatment outcomes. Z Gerontol Geriatr. 2007;40(3):178-84.

126. Mahlberg R, Walther S, Eichmann U, Tracik F, Kunz D. Effects of rivastigmine on actigraphically monitored motor activity in severe agitation related to Alzheimer's disease: a placebo-controlled pilot study. Archives of gerontology and geriatrics. 2007;45(1):19‐26.

127. Mäkinen N, Huuskonen U, Hannila E, Pisilä A-P, Alaniemi L, Koskinen K, et al. System validation study for novel wearable sleep apnea screening device. Sleep Medicine. 2022;100:S279.

128. Martinez-Nicolas A, Guaita M, Santamaria J, Montserrat JM, Madrid JA, Rol MA. Ambulatory circadian monitoring in sleep disordered breathing patients and CPAP treatment. Sci. 2021;11(1):14711.

129. Maskevich S, Jumabhoy R, Dao PDM, Stout JC, Drummond SPA. Pilot Validation of Ambulatory Activity Monitors for Sleep Measurement in Huntington's Disease Gene Carriers. J Huntingtons Dis. 2017;6(3):249-53.

130. Massot B, Baltenneck N, Gehin C, Dittmar A, McAdams E. Objective evaluation of stress with the blind by the monitoring of autonomic nervous system activity. Conference proceedings : . 2010;Annual International Conference of the IEEE Engineering in Medicine and Biology Society. IEEE Engineering in Medicine and Biology Society. Conference. 2010:1429-32.

131. McCall WV, Erman M, Krystal AD, Rosenberg R, Scharf M, Zammit GK, et al. A polysomnography study of eszopiclone in elderly patients with insomnia. Curr Med Res Opin. 2006;22(9):1633-42.

132. McClure K, Erdreich B, Bates JHT, McGinnis RS, Masquelin A, Wshah S. Classification and Detection of Breathing Patterns with Wearable Sensors and Deep Learning. Sensors (Basel). 2020;20(22).

133. Meadows R, Luff R, Eyers I, Venn S, Cope E, Arber S. An actigraphic study comparing community dwelling poor sleepers with non-demented care home residents. Chronobiol Int. 2010;27(4):842-54.

134. Mehra R, Stone KL, Ancoli-Israel S, Litwack-Harrison S, Ensrud KE, Redline S. Interpreting wrist actigraphic indices of sleep in epidemiologic studies of the elderly: the Study of Osteoporotic Fractures. Sleep. 2008;31(11):1569-76.

135. Melander CA, Kikhia B, Olsson M, Walivaara BM, Savenstedt S. The Impact of Using Measurements of Electrodermal Activity in the Assessment of Problematic Behaviour in Dementia. Dement Geriatr Cogn Dis Extra. 2018;8(3):333-47.

136. Minteer DM, Simon P, Taylor DP, Jia W, Li Y, Sun M, et al. Pressure Ulcer Monitoring Platform - A Prospective, Human Subject Clinical Study to Validate Patient Repositioning Monitoring Device to Prevent Pressure Ulcers. Advances in Wound Care. 2020;9(1):28-33.

137. Miranda D, Favela J, Arnrich B. Detecting Anxiety States when Caring for People with Dementia. Methods Inf Med. 2017;56(1):55-62.

138. Miranda D, Favela J, Ibarra C, Cruz N. Naturalistic Enactment to Elicit and Recognize Caregiver State Anxiety. J Med Syst. 2016;40(9):7.

139. Mokhtaran M, Sacchi L, Tibollo V, Risi I, Ramella V, Quaglini S, et al. Obstructive Sleep Apnea Home-Monitoring Using a Commercial Wearable Device. MEDINFO 2021: One World, One Health - Global Partnership for Digital Innovation - Proceedings of the 18th World Congress on Medical and Health Informatics Studies in Health Technology and Informatics. 2022;290:522-5.

140. Monreal-Carrillo E, Allende-Pérez S, Hui D, García-Salamanca MF, Bruera E, Verástegui E. Bispectral Index monitoring in cancer patients undergoing palliative sedation: a preliminary report. Support Care Cancer. 2017;25(10):3143-9.

141. Morales CR, Hurley S, Wick LC, Staley B, Pack FM, Gooneratne NS, et al. In-home, self-assembled sleep studies are useful in diagnosing sleep apnea in the elderly. Sleep. 2012;35(11):1491-501.

142. Most EI, Aboudan S, Scheltens P, Van Someren EJ. Discrepancy between subjective and objective sleep disturbances in early- and moderate-stage Alzheimer disease. Am J Geriatr Psychiatry. 2012;20(6):460-7.

143. Motoi K, Ogawa M, Ueno H, Kuwae Y, Ikarashi A, Yuji T, et al. A fully automated health-care monitoring at home without attachment of any biological sensors and its clinical evaluation. Conference proceedings : . 2009;Annual International Conference of the IEEE Engineering in Medicine and Biology Society. IEEE Engineering in Medicine and Biology Society. Conference.:4323-6.

144. Mulin E, Zeitzer JM, Friedman L, Le Duff F, Yesavage J, Robert PH, et al. Relationship between apathy and sleep disturbance in mild and moderate Alzheimer's disease: an actigraphic study. J Alzheimers Dis. 2011;25(1):85-91.

145. Nagels G, Engelborghs S, Vloeberghs E, Van Dam D, Pickut BA, De Deyn PP. Actigraphic measurement of agitated behaviour in dementia. International Journal of Geriatric Psychiatry. 2006;21(4):388-93.

146. Nakamura T, Alqurashi YD, Morrell MJ, Mandic DP. Hearables: Automatic Overnight Sleep Monitoring With Standardized In-Ear EEG Sensor. IEEE Trans Biomed Eng. 2020;67(1):203-12.

147. Nikoletti S, Young J, King M. Evaluation of an electronic monitoring device for urinary incontinence in elderly patients in an acute care setting. J Wound Ostomy Continence Nurs. 2004;31(3):138-49.

148. Onen SH, Dubray C, Decullier E, Moreau T, Chapuis F, Onen F. Observation-based nocturnal sleep inventory: screening tool for sleep apnea in elderly people. J Am Geriatr Soc. 2008;56(10):1920-5.

149. Ouslander JG, Buxton WG, Al-Samarrai NR, Cruise PA, Alessi C, Schnelle JF. Nighttime urinary incontinence and sleep disruption among nursing home residents. Journal of the American Geriatrics Society. 1998;46(4):463-6.

150. Palestra G, Pino O. Detecting emotions during a memory training assisted by a social robot for individuals with Mild Cognitive Impairment (MCI). Multimed Tools Appl. 2020;79(47-48):35829-44.

151. Pao WC, Boeve BF, Ferman TJ, Lin SC, Smith GE, Knopman DS, et al. Polysomnographic findings in dementia with Lewy bodies. Neurologist. 2013;19(1):1-6.

152. Park HJ, Choi D, Park HA, Lee CA. Nurse evaluation of stress levels during CPR training with heart rate variability using smartwatches according to their personality: A prospective, observational study. PLoS ONE. 2022;17(6 June) (no pagination).

153. Patout M, Arbane G, Cuvelier A, Muir JF, Hart N, Murphy PB. Polysomnography versus limited respiratory monitoring and nurse-led titration to optimise non-invasive ventilation set-up: A pilot randomised clinical trial. Thorax. 2019;74(1):83-6.

154. Pedrao RAA, Riella RJ, Richards K, Valderramas SR. Viability and validity of the bispectral index to measure sleep in patients in the intensive care unit. Rev. 2020;32(4):535-41.

155. Peterson MJ, Gravenstein N, Schwab WK, van Oostrom JH, Caruso LJ. Patient repositioning and pressure ulcer risk--monitoring interface pressures of at-risk patients. J Rehabil Res Dev. 2013;50(4):477-88.

156. Piano C, Della Marca G, Losurdo A, Imperatori C, Solito M, Calandra-Buonaura G, et al. Subjective Assessment of Sleep in Huntington Disease: Reliability of Sleep Questionnaires Compared to Polysomnography. Neurodegener Dis. 2017;17(6):330-7.

157. Piano C, Losurdo A, Della Marca G, Solito M, Calandra-Buonaura G, Provini F, et al. Polysomnographic Findings and Clinical Correlates in Huntington Disease: A Cross-Sectional Cohort Study. Sleep. 2015;38(9):1489-95.

158. Pickham D, Berte N, Pihulic M, Valdez A, Mayer B, Desai M. Effect of a wearable patient sensor on care delivery for preventing pressure injuries in acutely ill adults: a pragmatic randomized clinical trial (LS-HAPI study). International journal of nursing studies. 2018;80:12‐9.

159. Pittsley M, Gehrman P, Cohen-Zion M, Stepnowsky C, Marler M, Ancoli-Israel S. Comparing night-to-night variability of sleep measures in elderly African Americans and Whites. Behav Sleep Med. 2005;3(2):63-72.

160. Polese JF, Santos-Silva R, de Oliveira Ferrari PM, Sartori DE, Tufik S, Bittencourt L. Is portable monitoring for diagnosing obstructive sleep apnea syndrome suitable in elderly population? Sleep Breath. 2013;17(2):679-86.

161. Prasad B, Usmani S, Steffen AD, Van Dongen HPA, Pack FM, Strakovsky I, et al. Short-term variability in apnea-hypopnea index during extended home portable monitoring. Journal of Clinical Sleep Medicine. 2016;12(6):855-63.

162. Pu L, Lion KM, Todorovic M, Moyle W. Portable EEG monitoring for older adults with dementia and chronic pain - A feasibility study. Geriatr Nurs. 2021;42(1):124-8.

163. Quraishi SA, Blosser SA, Cherry RA. Bispectral index monitoring in the management of sedation in an intensive care unit patient with locked-in syndrome. American Journal of Critical Care. 2011;20(6):487-90.

164. Raj R, Ussavarungsi K, Nugent K. Accelerometer-based devices can be used to monitor sedation/agitation in the intensive care unit. J Crit Care. 2014;29(5):748-52.

165. Rajasekaran S, Luteran C, Qu H, Riley-Doucet C. A portable autonomous multisensory intervention device (PAMID) for early detection of anxiety and agitation in patients with cognitive impairments. Annu Int Conf IEEE Eng Med Biol Soc. 2011;2011:4733-6.

166. Ramirez-Moreno MA, Carrillo-Tijerina P, Candela-Leal MO, Alanis-Espinosa M, Tudon-Martinez JC, Roman-Flores A, et al. Evaluation of a Fast Test Based on Biometric Signals to Assess Mental Fatigue at the Workplace-A Pilot Study. International Journal of Environmental Research and Public Health. 2021;18(22):20.

167. Ravindran KKG, Monica CD, Atzori G, Enshaeifar S, Mahvash-Mohammadi S, Dijk DJ, et al. Validation of technology to monitor sleep and bed occupancy in older men and women. Alzheimer's & dementia : the journal of the Alzheimer's Association. 2021;17(Supplement 8):e056018.

168. Ravishankar H, Saha A, Swamy G, Genc S. An early respiratory distress detection method with Markov models. Annu Int Conf IEEE Eng Med Biol Soc. 2014;2014:3438-41.

169. Resuli N, Skubic M, Myungki J, editors. Noninvasive respiration monitoring of different sleeping postures using an rf sensor. 2021 IEEE International Conference on Bioinformatics and Biomedicine (BIBM); 2021: IEEE.

170. Rezaei S, Moturu A, Zhao S, Prkachin KM, Hadjistavropoulos T, Taati B. Unobtrusive Pain Monitoring in Older Adults with Dementia using Pairwise and Contrastive Training. IEEE J Biomed Health Inform. 2021;Pp.

171. Roh T, Bong K, Hong S, Cho H, Yoo HJ. Wearable mental-health monitoring platform with independent component analysis and nonlinear chaotic analysis. Conference proceedings : . 2012;Annual International Conference of the IEEE Engineering in Medicine and Biology Society. IEEE Engineering in Medicine and Biology Society. Conference.:4541-4.

172. Rose K, Specht J, Forch W. Correlates among nocturnal agitation, sleep, and urinary incontinence in dementia. Am J Alzheimers Dis Other Demen. 2015;30(1):78-84.

173. Rotariu C, Costin H. Remote respiration monitoring system for sleep apnea detection. Revista medico-chirurgicala a Societatii de Medici si Naturalisti din Iasi. 2013;117(1):268-74.

174. Rowe MA, Kairalla JA, McCrae CS. Sleep in dementia caregivers and the effect of a nighttime monitoring system. J Nurs Scholarsh. 2010;42(3):338-47.

175. Sakai K, Sanada H, Matsui N, Nakagami G, Sugama J, Komiyama C, et al. Continuous monitoring of interface pressure distribution in intensive care patients for pressure ulcer prevention. J Adv Nurs. 2009;65(4):809-17.

176. Sato R, Kanda K, Anan M, Watanuki S. Sleep EEG patterns and fatigue of middle-aged and older female family caregivers providing routine nighttime care for elderly persons at home. Percept Mot Skills. 2002;95(3 Pt 1):815-29.

177. Schellenberger S, Shi KL, Steigleder T, Malessa A, Michler F, Hameyer L, et al. A dataset of clinically recorded radar vital signs with synchronised reference sensor signals. Sci Data. 2020;7(1).

178. Setz C, Arnrich B, Schumm J, La Marca R, Troster G, Ehlert U. Discriminating stress from cognitive load using a wearable EDA device. IEEE transactions on information technology in biomedicine : a publication of the IEEE Engineering in Medicine and Biology Society. 2010;14(2):410-7.

179. Six S, Laureys S, Poelaert J, Bilsen J, Theuns P, Musch L, et al. Should we include monitors to improve assessment of awareness and pain in unconscious palliatively sedated patients? A case report. Palliative Medicine. 2019;33(6):712-6.

180. Six S, Laureys S, Poelaert J, Maîresse O, Theuns P, Bilsen J, et al. Neurophysiological assessments during continuous sedation until death put validity of observational assessments into question: a prospective observational study. Pain and therapy. 2021;10(1):377-90.

181. Six S, Van Overmeire R, Bilsen J, Laureys S, Poelaert J, Theuns P, et al. Attitudes of Professional Caregivers and Family Members Regarding the Use of Monitoring Devices to Improve Assessments of Pain and Discomfort During Continuous Sedation Until Death. J Pain Symptom Manage. 2020;60(2):390-9.

182. Smith JH, Baumert M, Nalivaiko E, McEvoy RD, Catcheside PG. Arousal in obstructive sleep apnoea patients is associated with ECG RR and QT interval shortening and PR interval lengthening. J Sleep Res. 2009;18(2):188-95.

183. Soderstrom M, Ekstedt M, Akerstedt T, Nilsson J, Axelsson J. Leep and sleepiness in young individuals with high burnout scores. Sleep. 2004;27(7):1369-77.

184. Sofronova D, Angelova RA, Sofronov Y. Design and Development of an E-Textile Mat for Assuring the Comfort of Bedridden Persons. Materials. 2021;14(18):11.

185. Spasojevic S, Nogas J, Iaboni A, Ye B, Mihailidis A, Wang A, et al. A Pilot Study to Detect Agitation in People Living with Dementia Using Multi-Modal Sensors. J Healthc Inform Res. 2021;5(3):342-58.

186. Spira AP, Stone KL, Redline S, Ensrud KE, Ancoli-Israel S, Cauley JA, et al. Actigraphic Sleep Duration and Fragmentation in Older Women: Associations With Performance Across Cognitive Domains. Sleep. 2017;40(8).

187. Stavitsky K, Saurman JL, McNamara P, Cronin-Golomb A. Sleep in Parkinson's disease: a comparison of actigraphy and subjective measures. Parkinsonism Relat Disord. 2010;16(4):280-3.

188. Supe D, Baron L, Decker T, Parker K, Venella J, Williams S, et al. Research: Continuous Surveillance of Sleep Apnea Patients in a Medical-Surgical Unit. Biomed Instrum Technol. 2017;51(3):236-51.

189. Svetnik V, Wang TC, Ceesay P, Snyder E, Ceren O, Bliwise D, et al. Pilot evaluation of a consumer wearable device to assess sleep in a clinical polysomnography trial of suvorexant for treating insomnia in patients with Alzheimer's disease. J Sleep Res. 2021;30(6):e13328.

190. Sylvia LG, Salcedo S, Bianchi MT, Urdahl AK, Nierenberg AA, Deckersbach T. A novel home sleep monitoring device and brief sleep intervention for bipolar disorder: Feasibility, tolerability, and preliminary effectiveness. Cognitive Therapy and Research. 2014;38(1):55-61.

191. Tao L, Yi YP, Shan Y, Yu D, Zhang J, Qu YS, et al. Analysis on severe fever with thrombocytopenia syndrome bunyavirus infection combined with atrial fibrillation under digital model detection. Results Phys. 2021;26:8.

192. Targa A, Dakterzada F, Benitez ID, de Gonzalo-Calvo D, Moncusi-Moix A, Lopez R, et al. Circulating MicroRNA Profile Associated with Obstructive Sleep Apnea in Alzheimer's Disease. Molecular Neurobiology. 2020;57(11):4363-72.

193. Tateishi O, Okamura T, Itou T, Murakami M, Suda T, Nishimuta I, et al. Observation of sleep-related breathing disorders in patients with coronary artery disease by ambulatory electrocardiogram-respiration monitoring system. Jpn Circ J. 1994;58(11):831-5.

194. Tedeschi E, Carratu P, Damiani MF, Ventura VA, Drigo R, Enzo E, et al. Home unattended portable monitoring and automatic CPAP titration in patients with high risk for moderate to severe obstructive sleep apnea. Respiratory Care. 2013;58(7):1179-83.

195. Tejedor B, Casals M, Gangolells M, Macarulla M, Forcada N. Human comfort modelling for elderly people by infrared thermography: Evaluating the thermoregulation system responses in an indoor environment during winter. Build Environ. 2020;186:18.

196. Tekcin M, Sayar E, Yalcin MK, Bahadir SK. Wearable and Flexible Humidity Sensor Integrated to Disposable Diapers for Wetness Monitoring and Urinary Incontinence. Electronics. 2022;11(7):14.

197. Terzaghi M, Arnaldi D, Rizzetti MC, Minafra B, Cremascoli R, Rustioni V, et al. Analysis of video-polysomnographic sleep findings in dementia with Lewy bodies. Mov Disord. 2013;28(10):1416-23.

198. Tiihonen P, Kinnunen J, Töyräs J, Mervaala E, Pääkkönen A. A portable device for intensive care brain function monitoring with event-related potentials. Computer Methods & Programs in Biomedicine. 2008;89(1):83-92.

199. To KW, Chan TO, Chan WC, Choo KL, Hui DSC. Using a portable monitoring device for diagnosing obstructive sleep apnea in patients with multiple coexisting medical illnesses. Clinical Respiratory Journal. 2021.

200. Toba K, Sudo N, Nagano K, Eto M, Kozaki K, Akishita M, et al. Use of a micturition-monitoring device in elderly inpatients. Nihon Ronen Igakkai zasshi Japanese Journal of Geriatrics. 1996;33(9):681-5.

201. Tong Y, Zhang Q, Cheng C, She C, Song W, Cui S. [Analysis of monitoring results of Mattress-type of sleep monitoring system in elderly patients with OSAHS]. Lin Chung Er Bi Yan Hou Tou Jing Wai Ke Za Zhi. 2015;29(18):1615-7.

202. Urdanibia-Centelles O, Nielsen RM, Rostrup E, Vedel-Larsen E, Thomsen K, Nikolic M, et al. Automatic continuous EEG signal analysis for diagnosis of delirium in patients with sepsis. Clin Neurophysiol. 2021;132(9):2075-82.

203. Vacas S, McInrue E, Gropper MA, Maze M, Zak R, Lim E, et al. The Feasibility and Utility of Continuous Sleep Monitoring in Critically Ill Patients Using a Portable Electroencephalography Monitor. Anesth Analg. 2016;123(1):206-12.

204. Valembois L, Oasi C, Pariel S, Jarzebowski W, Lafuente-Lafuente C, Belmin J. Wrist actigraphy: A simple way to record motor activity in elderly patients with dementia and apathy or aberrant motor behavior. J Nutr Health Aging. 2015;19(7):759-64.

205. van den Berg JF, Miedema HM, Tulen JH, Hofman A, Neven AK, Tiemeier H. Sex differences in subjective and actigraphic sleep measures: a population-based study of elderly persons. Sleep. 2009;32(10):1367-75.

206. Van Den Berg JF, Van Rooij FJ, Vos H, Tulen JH, Hofman A, Miedema HM, et al. Disagreement between subjective and actigraphic measures of sleep duration in a population-based study of elderly persons. J Sleep Res. 2008;17(3):295-302.

207. van der Hurk PR, Middelkoop HA, van Waalwijk-van Doorn ES, Roos RA, Cools HJ. Long-term ambulatory monitoring of urine leakage in the elderly: an evaluation of the validity and clinical applicability of thermistor signalling. J Med Eng Technol. 1998;22(2):91-3.

208. van Dijk E, Hilgenkamp TI, Evenhuis HM, Echteld MA. Exploring the use of actigraphy to investigate sleep problems in older people with intellectual disability. J Intellect Disabil Res. 2012;56(2):204-11.

209. van Hilten B, Hoff JI, Middelkoop HA, van der Velde EA, Kerkhof GA, Wauquier A, et al. Sleep disruption in Parkinson's disease. Assessment by continuous activity monitoring. Arch Neurol. 1994;51(9):922-8.

210. Van Someren EJ, Oosterman J, Van Harten B, Vogels R, Gouw A, Weinstein H, et al. Medial temporal lobe atrophy relates more strongly to sleep-wake rhythm fragmentation than to age or any other known risk. Neurobiology of learning and memory. 2019;160:132-8.

211. Várady P, Micsik T, Benedek S, Benyó Z. A novel method for the detection of apnea and hypopnea events in respiration signals. IEEE Trans Biomed Eng. 2002;49(9):936-42.

212. Varri A, Koivuluoma M, Morvan C. A computer-assisted visual sleep scoring program. Stud Health Technol Inform. 2000;78:285-97.

213. Wai AA, Fook VF, Jayachandran M, Biswas J, Nugent C, Mulvenna M, et al. Smart wireless continence management system for persons with dementia. Telemed J E Health. 2008;14(8):825-32.

214. Walsh JK, Salkeld L, Knowles LJ, Tasker T, Hunneyball IM. Treatment of elderly primary insomnia patients with EVT 201 improves sleep initiation, sleep maintenance, and daytime sleepiness. Sleep Med. 2010;11(1):23-30.

215. Wang B, Han F, Li Q, Chen X, Li J, An P, et al. [Value of pulse oximetry for diagnosing obstructive sleep apnea-hypopnea syndrome and evaluation of the effect of the continuous positive airway pressure therapy]. Zhonghua Yi Xue Za Zhi. 2015;95(40):3273-6.

216. Wang D, Timm GW, Erdman AG, Tewfik AH. Ambulatory device for urinary incontinence detection in females. Conference proceedings : . 2009;Annual International Conference of the IEEE Engineering in Medicine and Biology Society. IEEE Engineering in Medicine and Biology Society. Conference. 2009:5405-8.

217. Wang S, Cui H, Song C, Zhu C, Wu R, Meng L, et al. Obstructive sleep apnea is associated with nonsustained ventricular tachycardia in patients with hypertrophic obstructive cardiomyopathy. Heart Rhythm. 2019;16(5):694-701.

218. Watanabe T, Matsuura T, Watanabe M, Dote T, Simizu H, Kono K. The relationship between ambulatory blood pressure variation and symptoms of depression and sleep disturbance in community-dwelling elderly persons with independent activities of daily living. [Nihon Koshu Eisei Zasshi] Japanese Journal of Public Health. 2002;49(3):178-87.

219. Westerberg CE, Lundgren EM, Florczak SM, Mesulam MM, Weintraub S, Zee PC, et al. Sleep influences the severity of memory disruption in amnestic mild cognitive impairment: Results from Sleep self-assessment and continuous activity monitoring. Alzheimer Disease and Associated Disorders. 2010;24(4):325-33.

220. Wijsman J, Grundlehner B, Liu H, Hermens H, Penders J. Towards mental stress detection using wearable physiological sensors. Conference proceedings : . 2011;Annual International Conference of the IEEE Engineering in Medicine and Biology Society. IEEE Engineering in Medicine and Biology Society. Conference.:1798-801.

221. Wilcock A, England R, El Khoury B, Frisby J, Howard P, Bell S, et al. The prevalence of nocturnal hypoxemia in advanced cancer. J Pain Symptom Manage. 2008;36(4):351-7.

222. Wilcox ME, Rubenfeld GD, Walczak KD, Black SE, McAndrews MP, Lim AS. Actigraphic measures of sleep on the wards after ICU discharge. J Crit Care. 2019;54:163-9.

223. Wu W, Gil Y, Lee J. Combination of wearable multi-biosensor platform and resonance frequency training for stress management of the unemployed population. Sensors (Switzerland). 2012;12(10):13225-48.

224. Xu L, Han F, Keenan BT, Kneeland-Szanto E, Yan H, Dong X, et al. Validation of the Nox-T3 portable monitor for diagnosis of obstructive sleep apnea in Chinese adults. Journal of Clinical Sleep Medicine. 2017;13(5):675-83.

225. Xun YF, Wang MH, Sun HY, Guan B. [Comparative analysis of sleep monitoring between young and middle-aged and elderly OSA patients]. Lin Chung Er Bi Yan Hou Tou Jing Wai Ke Za Zhi. 2019;33(7):643-6.

226. Yaffe K, Blackwell T, Barnes DE, Ancoli-Israel S, Stone KL, Study Osteoporotic Fractures G. Preclinical cognitive decline and subsequent sleep disturbance in older women. Neurology. 2007;69(3):237-42.

227. Yang G, Jiang M, Ouyang W, Ji G, Xie H, Rahmani AM, et al. IoT-Based Remote Pain Monitoring System: From Device to Cloud Platform. IEEE J Biomed Health Inform. 2018;22(6):1711-9.

228. Yesavage JA, Noda A, Heath A, McNerney MW, Domingue BW, Hernandez Y, et al. Sleep-wake disorders in Alzheimer's disease: Further genetic analyses in relation to objective sleep measures. International Psychogeriatrics. 2020;32(7):807-13.

229. Zhang Y, Wang W, Cai S, Sheng Q, Pan S, Shen F, et al. Obstructive sleep apnea exaggerates cognitive dysfunction in stroke patients. Sleep Med. 2017;33:183-90.
