# Supplementary material for: Noninvasive monitoring technologies to identify discomfort and distressing symptoms in persons with limited communication at the end of life: a scoping review
Source: BMC Palliat Care. 2024 Mar 21;23:78. doi: 10.1186/s12904-024-01371-0 (PMC10956214; doi:10.1186/s12904-024-01371-0)
Supplement: Supplementary file 5 — Additional file 5. Search strategy. [file 12904_2024_1371_MOESM5_ESM.docx]

**Supplement V of “Noninvasive monitoring technologies to identify discomfort and distressing symptoms in persons with limited communication at the end of life: A scoping review”**

Search strategy for “Noninvasive monitoring technologies to identify discomfort and distressing symptoms in persons with limited communication at the end of life: a scoping review ”

**Main topics:** Non-invasive monitoring AND distressing symptoms.

**Other topics:** Dementia OR palliative care OR critically ill OR profound intellectual and multiple disability OR bedridden

**Details of the search strategies for each database:**

[PubMed: 1](#_Toc70083640)

[MEDLINE (OVID) 4](#_Toc70083641)

[Embase (OVID) 6](#_Toc70083642)

[Web of Science 8](#_Toc70083643)

[Cochrane Library 10](#_Toc70083644)

[Emcare (OVID) 14](#_Toc70083645)

[PsycINFO (IEbscoHOST) 16](#_Toc70083646)

[Academic Search Premier (EbscoHOST) 21](#_Toc70083647)

[Google Scholar 26](#_Toc70083648)

## PubMed:

(("Dementia"[Mesh] OR "Dementia"[tw] OR "dement*"[tw] OR "Alzheimer Disease"[tw] OR "Alzheimer*"[tw] OR "CADASIL"[tw] OR "Creutzfeldt-Jakob"[tw] OR "Creutzfeldt-Jakob Syndrome"[tw] OR "Diffuse Neurofibrillary Tangles with Calcification"[tw] OR "Frontotemporal Lobar Degeneration"[tw] OR "Huntington Disease"[tw] OR "Huntington*"[tw] OR "Kluver-Bucy"[tw] OR "Kluver-Bucy Syndrome"[tw] OR "Lewy Body"[tw] OR "Lewy Body Disease"[tw] OR "Pick Disease of the Brain"[tw] OR "Primary Progressive Aphasia"[tw] OR "Primary Progressive Nonfluent Aphasia"[tw] OR "Death"[Mesh] OR "dying"[tw] OR "end of life phase"[tw] OR "end of life"[tw] OR "end-of-life phase"[tw] OR "endoflife"[tw] OR "end-of-life"[tw] OR "Hospice and Palliative Care Nursing"[mesh] OR "Hospice Care"[Mesh] OR "Hospice Care"[tw] OR "Hospice Care"[tw] OR "Hospice"[tw] OR "Hospices"[Mesh] OR "Hospices"[tw] OR "palliat*"[tw] OR "Palliative Care"[Mesh] OR "Palliative Care"[tw] OR "Palliative Medicine"[mesh] OR "Palliative Phase"[tw] OR "Palliative Phases"[tw] OR "Palliative Stage"[tw] OR "Palliative Stages"[tw] OR "Palliative Supportive Care"[tw] OR "Palliative Surgery"[tw] OR "Palliative Therapy"[tw] OR "Palliative Treatment"[tw] OR "Palliative Treatments"[tw] OR "Terminal Care"[Mesh] OR "Terminal Care"[tw] OR "terminal stage"[tw] OR "elderly"[ti] OR "critical care"[mesh] OR "critical care"[tw] OR "intensive care"[tw] OR "Critical Illness"[Mesh] OR "Critical Illness"[tw] OR "critically ill"[tw] OR (("profound"[tw] OR "profound*"[tw]) AND ("Disabled Persons"[Mesh] OR "Intellectual Disability"[Mesh])) OR (("profound"[tw] OR "profound*"[tw]) AND ("intellectual"[tw] OR "intellectualism"[tw] OR "intellectually"[tw] OR "intellectuals"[tw]) AND ("multiple"[tw] OR "multiples"[tw]) AND ("disabilities"[tw] OR "disability"[tw] OR "disabled persons"[MeSH Terms] OR "disabled persons"[tw] OR "disabled"[tw] OR "disablement"[tw] OR "disablements"[tw] OR "disabling"[tw] OR "disablity"[tw])) OR "Bedridden Persons"[Mesh] OR "Bedridden"[tw] OR "Non-Mobile Person"[tw] OR "Non-Mobile Persons"[tw]) AND ("activity monitor"[ti] OR "activity monitors"[ti] OR "activity monitoring"[ti] OR "activity tracker"[ti] OR "activity trackers"[ti] OR "activity tracking"[ti] OR "Ambulatory Monitor*"[ti] OR "Ambulatory Monitoring"[ti] OR "Electronic Skin"[ti] OR (("monitor*"[ti] OR "monitoring"[ti]) AND ("Telemedicine"[majr] OR "telemed*"[ti] OR "telehealth*"[ti] OR "technol*"[ti])) OR "monitoring app"[ti] OR "monitoring application"[ti] OR "monitoring applications"[ti] OR "monitoring apps"[ti] OR "monitoring device"[ti] OR "monitoring devices"[ti] OR "monitoring technologies"[ti] OR "monitoring technology"[ti] OR "Monitoring, Ambulatory"[majr] OR "Monitoring, Physiologic"[majr] OR "Monitoring, Physiologicinstrumentation"[majr] OR "Noninvasive device"[ti] OR "Non-invasive device"[ti] OR "Noninvasive devices"[ti] OR "Non-invasive devices"[ti] OR "Noninvasive monitoring device"[ti] OR "Noninvasive monitoring device"[ti] OR "Non-invasive monitoring device"[ti] OR "Noninvasive monitoring devices"[ti] OR "Noninvasive monitoring devices"[ti] OR "Non-invasive monitoring devices"[ti] OR "Noninvasive monitoring technologies"[ti] OR "Noninvasive monitoring technologies"[ti] OR "Non-invasive monitoring technologies"[ti] OR "Noninvasive monitoring technology"[ti] OR "Noninvasive monitoring technology"[ti] OR "Non-invasive monitoring technology"[ti] OR "Noninvasive monitoring"[ti] OR "Non-invasive monitoring"[ti] OR "Noninvasive technologies"[ti] OR "Non-invasive technologies"[ti] OR "Noninvasive technology"[ti] OR "Non-invasive technology"[ti] OR "Outpatient Monitor*"[ti] OR "Outpatient Monitoring"[ti] OR "Portable Electronic Application"[ti] OR "Portable Electronic Applications"[ti] OR "Portable Software Application"[ti] OR "Portable Software Applications"[ti] OR "Smart watch"[ti] OR "Smart watches"[ti] OR "Smartwatch"[ti] OR "Smartwatches"[ti] OR "tracker"[ti] OR "trackers"[ti] OR "wearab*"[ti] OR "wearable activity tracker"[ti] OR "wearable activity trackers"[ti] OR "wearable activity tracking"[ti] OR "Wearable Device"[ti] OR "Wearable Devices"[ti] OR "Wearable Electronic Device"[ti] OR "Wearable Electronic Devices"[majr] OR "Wearable Electronic Devices"[ti] OR "Wearable Technologies"[ti] OR "Wearable Technology"[ti] OR "wearable"[ti] OR "wearables"[ti] OR "noninvasive body sensor"[ti] OR "noninvasive body sensors"[ti] OR "non invasive body sensor"[ti] OR "non invasive body sensors"[ti] OR "body sensor"[ti] OR "body sensors"[ti] OR "wristband sensor"[ti] OR "wristband sensors"[ti] OR "wearable computing"[ti] OR "wearable computer"[ti] OR "wearable computers"[ti] OR "point-of-care App"[ti] OR "point-of-care Apps"[ti] OR "point-of-care Application"[ti] OR "point-of-care Applications"[ti] OR "facial recognition technology"[ti] OR "face recognition technology"[ti] OR "Point-of-Care Technology"[ti] OR "Point-of-Care Techn*"[ti] OR "HUME"[ti] OR "ePAT"[ti] OR " electronic pain assessment"[ti] OR ("BIS"[ti] AND "bispectral"[ti]) OR "bispectral index"[ti] OR "Consciousness Monitors"[majr] OR "Biosensing Techniques"[majr] OR ("Facial Expression"[majr] AND "Point-of-Care Systems"[majr]) OR (("noninvasiv*"[ti] OR "non invasiv*"[ti] OR "unobtrusiv*"[ti] OR "portable"[ti] OR "Mobile Applications"[Mesh]) AND "monitor*"[ti]) OR "Painchek"[tw] OR "Paincheck"[tw] OR "automated facial analysis"[tw] OR "automated facial recognition"[tw] OR "automated facial expression analysis"[tw]) AND ("discomfort"[ti] OR "discomfort*"[ti] OR "Psychological Distress"[majr] OR "distressing symptoms"[ti] OR "distressing symptom"[ti] OR "distressing"[ti] OR "distress"[ti] OR "Patient Comfort"[majr] OR "comfort"[ti] OR "comfort*"[ti] OR "Psychomotor Agitation"[majr] OR "agitation"[ti] OR "agitat*"[ti] OR "restlessness"[ti] OR "restless*"[ti] OR "hyperactivity"[ti] OR "hyperactiv*"[ti] OR "akathisia"[ti] OR "Pain"[majr] OR "pain"[ti] OR "Pain Measurement"[majr] OR "pain assessment"[ti] OR "Stress, Psychological"[majr] OR "stress level"[ti] OR "stress levels"[ti] OR "pressure ulcer"[ti] OR "pressure ulcer"[ti] OR "life quality"[ti] OR "quality of life"[majr] OR "quality of life"[ti] OR "breathing difficulty"[ti] OR "difficulty breathing"[ti] OR "difficult breathing"[ti] OR "labored breathing"[ti] OR "laboured breathing"[ti] OR "Shortness of Breath"[ti] OR "Breath Shortness"[ti] OR "Breathlessness"[ti] OR "Dyspnea"[majr] OR "Dyspnea"[ti] OR "Anxiety"[majr] OR "Anxiety"[ti] OR "Nervousness"[ti] OR "Hypervigilance"[ti] OR "Anxiousness"[ti] OR "Sleep Wake Disorders"[majr] OR "sleep disturbances"[ti] OR "sleep disturbance"[ti] OR "Fatigue"[majr] OR "fatigue"[ti] OR "Nausea"[majr] OR "nausea"[ti] OR "nauseous"[ti] OR "Delirium"[majr] OR "delirium"[ti] OR "delirious"[ti] OR "Fever"[majr] OR "fever"[ti] OR "Urinary Incontinence"[majr] OR "Incontinence"[ti] OR "incontinen*"[ti] OR "Sedation depth"[ti] OR "Sedation depths"[ti] OR "Depth of sedation"[ti]))

## MEDLINE (OVID)

(exp "Dementia"/ OR "Dementia".mp OR "dement*".mp OR "Alzheimer Disease".mp OR "Alzheimer*".mp OR "CADASIL".mp OR "Creutzfeldt-Jakob".mp OR "Creutzfeldt-Jakob Syndrome".mp OR "Diffuse Neurofibrillary Tangles with Calcification".mp OR "Frontotemporal Lobar Degeneration".mp OR "Huntington Disease".mp OR "Huntington*".mp OR "Kluver-Bucy".mp OR "Kluver-Bucy Syndrome".mp OR "Lewy Body".mp OR "Lewy Body Disease".mp OR "Pick Disease of the Brain".mp OR "Primary Progressive Aphasia".mp OR "Primary Progressive Nonfluent Aphasia".mp OR exp "Death"/ OR "dying".mp OR "end of life phase".mp OR "end of life".mp OR "end-of-life phase".mp OR "endoflife".mp OR "end-of-life".mp OR exp "Hospice AND Palliative Care Nursing"/ OR exp "Hospice Care"/ OR "Hospice Care".mp OR "Hospice Care".mp OR "Hospice".mp OR exp "Hospices"/ OR "Hospices".mp OR "palliat*".mp OR exp "Palliative Care"/ OR "Palliative Care".mp OR exp "Palliative Medicine"/ OR "Palliative Phase".mp OR "Palliative Phases".mp OR "Palliative Stage".mp OR "Palliative Stages".mp OR "Palliative Supportive Care".mp OR "Palliative Surgery".mp OR "Palliative Therapy".mp OR "Palliative Treatment".mp OR "Palliative Treatments".mp OR exp "Terminal Care"/ OR "Terminal Care".mp OR "terminal stage".mp OR "elderly".ti. OR exp "critical care"/ OR "critical care".mp OR "intensive care".mp OR exp "Critical Illness"/ OR "Critical Illness".mp OR "critically ill".mp OR (("profound" OR "profound*").mp AND (exp "Disabled Persons"/ OR exp "Intellectual Disability"/)) OR ((("profound" OR "profound*") AND ("intellectual" OR "intellectualism" OR "intellectually" OR "intellectuals") AND ("multiple" OR "multiples")).mp AND (("disabilities" OR "disability").mp OR exp "disabled persons"/ OR "disabled persons".mp OR "disabled".mp OR "disablement".mp OR "disablements".mp OR "disabling".mp OR "disablity".mp)) OR exp "Bedridden Persons"/ OR "Bedridden".mp OR "Non-Mobile Person".mp OR "Non-Mobile Persons".mp) AND (("activity monitor" OR "activity monitors" OR "activity monitoring" OR "activity tracker" OR "activity trackers" OR "activity tracking" OR "Ambulatory Monitor*" OR "Ambulatory Monitoring" OR "Electronic Skin").ti. OR (("monitor*" OR "monitoring").ti. AND (exp *"Telemedicine"/ OR "telemed*".ti. OR "telehealth*".ti. OR "technol*".ti.)) OR "monitoring app".ti. OR "monitoring application".ti. OR "monitoring applications".ti. OR "monitoring apps".ti. OR "monitoring device".ti. OR "monitoring devices".ti. OR "monitoring technologies".ti. OR "monitoring technology".ti. OR exp *"Monitoring, Ambulatory"/ OR exp *"Monitoring, Physiologic"/ OR "Noninvasive device".ti. OR "Non-invasive device".ti. OR "Noninvasive devices".ti. OR "Non-invasive devices".ti. OR "Noninvasive monitoring device".ti. OR "Noninvasive monitoring device".ti. OR "Non-invasive monitoring device".ti. OR "Noninvasive monitoring devices".ti. OR "Noninvasive monitoring devices".ti. OR "Non-invasive monitoring devices".ti. OR "Noninvasive monitoring technologies".ti. OR "Noninvasive monitoring technologies".ti. OR "Non-invasive monitoring technologies".ti. OR "Noninvasive monitoring technology".ti. OR "Noninvasive monitoring technology".ti. OR "Non-invasive monitoring technology".ti. OR "Noninvasive monitoring".ti. OR "Non-invasive monitoring".ti. OR "Noninvasive technologies".ti. OR "Non-invasive technologies".ti. OR "Noninvasive technology".ti. OR "Non-invasive technology".ti. OR "Outpatient Monitor*".ti. OR "Outpatient Monitoring".ti. OR "Portable Electronic Application".ti. OR "Portable Electronic Applications".ti. OR "Portable Software Application".ti. OR "Portable Software Applications".ti. OR "Smart watch".ti. OR "Smart watches".ti. OR "Smartwatch".ti. OR "Smartwatches".ti. OR "tracker".ti. OR "trackers".ti. OR "wearab*".ti. OR "wearable activity tracker".ti. OR "wearable activity trackers".ti. OR "wearable activity tracking".ti. OR "Wearable Device".ti. OR "Wearable Devices".ti. OR "Wearable Electronic Device".ti. OR exp *"Wearable Electronic Devices"/ OR "Wearable Electronic Devices".ti. OR "Wearable Technologies".ti. OR "Wearable Technology".ti. OR "wearable".ti. OR "wearables".ti. OR "noninvasive body sensor".ti. OR "noninvasive body sensors".ti. OR "non invasive body sensor".ti. OR "non invasive body sensors".ti. OR "body sensor".ti. OR "body sensors".ti. OR "wristband sensor".ti. OR "wristband sensors".ti. OR "wearable computing".ti. OR "wearable computer".ti. OR "wearable computers".ti. OR "point-of-care App".ti. OR "point-of-care Apps".ti. OR "point-of-care Application".ti. OR "point-of-care Applications".ti. OR "facial recognition technology".ti. OR "face recognition technology".ti. OR "Point-of-Care Technology".ti. OR "Point-of-Care Techn*".ti. OR "HUME".ti. OR "ePAT".ti. OR " electronic pain assessment".ti. OR ("BIS" AND "bispectral").ti. OR "bispectral index".ti. OR exp *"Consciousness Monitors"/ OR exp *"Biosensing Techniques"/ OR (exp *"Facial Expression"/ AND exp *"Point-of-Care Systems"/) OR ((("noninvasiv*" OR "non invasiv*" OR "unobtrusiv*" OR "portable").ti. OR exp "Mobile Applications"/) AND "monitor*".ti.) OR "Painchek".mp OR "Paincheck".mp OR "automated facial analysis".mp OR "automated facial recognition".mp OR "automated facial expression analysis".mp) AND (("discomfort" OR "discomfort*").ti. OR exp *"Psychological Distress"/ OR "distressing symptoms".ti. OR "distressing symptom".ti. OR "distressing".ti. OR "distress".ti. OR exp *"Patient Comfort"/ OR "comfort".ti. OR "comfort*".ti. OR exp *"Psychomotor Agitation"/ OR "agitation".ti. OR "agitat*".ti. OR "restlessness".ti. OR "restless*".ti. OR "hyperactivity".ti. OR "hyperactiv*".ti. OR "akathisia".ti. OR exp *"Pain"/ OR "pain".ti. OR exp *"Pain Measurement"/ OR "pain assessment".ti. OR exp *"Stress, Psychological"/ OR "stress level".ti. OR "stress levels".ti. OR "pressure ulcer".ti. OR "pressure ulcer".ti. OR "life quality".ti. OR exp *"quality of life"/ OR "quality of life".ti. OR "breathing difficulty".ti. OR "difficulty breathing".ti. OR "difficult breathing".ti. OR "labored breathing".ti. OR "laboured breathing".ti. OR "Shortness of Breath".ti. OR "Breath Shortness".ti. OR "Breathlessness".ti. OR exp *"Dyspnea"/ OR "Dyspnea".ti. OR exp *"Anxiety"/ OR "Anxiety".ti. OR "Nervousness".ti. OR "Hypervigilance".ti. OR "Anxiousness".ti. OR exp *"Sleep Wake Disorders"/ OR "sleep disturbances".ti. OR "sleep disturbance".ti. OR exp *"Fatigue"/ OR "fatigue".ti. OR exp *"Nausea"/ OR "nausea".ti. OR "nauseous".ti. OR exp *"Delirium"/ OR "delirium".ti. OR "delirious".ti. OR exp *"Fever"/ OR "fever".ti. OR exp *"Urinary Incontinence"/ OR "Incontinence".ti. OR "incontinen*".ti. OR "Sedation depth".ti. OR "Sedation depths".ti. OR "Depth of sedation".ti.)

## Embase (OVID)

((exp *"Dementia"/ OR ("Dementia" OR "dement*" OR "Alzheimer Disease" OR "Alzheimer*" OR "CADASIL" OR "Creutzfeldt-Jakob" OR "Creutzfeldt-Jakob Syndrome" OR "Diffuse Neurofibrillary Tangles with Calcification" OR "Frontotemporal Lobar Degeneration" OR "Huntington Disease" OR "Huntington*" OR "Kluver-Bucy" OR "Kluver-Bucy Syndrome" OR "Lewy Body" OR "Lewy Body Disease" OR "Pick Disease of the Brain" OR "Primary Progressive Aphasia" OR "Primary Progressive Nonfluent Aphasia").ti,ab OR exp *"Death"/ OR exp *"Terminal Care"/ OR exp *"Hospice"/ OR exp *"Palliative Therapy"/ OR ("dying" OR "end of life phase" OR "end of life" OR "end-of-life phase" OR "endoflife" OR "end-of-life" OR "Hospice and Palliative Care Nursing" OR "Hospice Care" OR "Hospice Care" OR "Hospice Care" OR "Hospice" OR "Hospices" OR "Hospices" OR "palliat*" OR "Palliative Care" OR "Palliative Care" OR "Palliative Medicine" OR "Palliative Phase" OR "Palliative Phases" OR "Palliative Stage" OR "Palliative Stages" OR "Palliative Supportive Care" OR "Palliative Surgery" OR "Palliative Therapy" OR "Palliative Treatment" OR "Palliative Treatments" OR "Terminal Care" OR "Terminal Care" OR "terminal stage").ti,ab OR "elderly".ti OR exp *"Intensive Care"/ OR exp *"Critical Illness"/ OR exp *"critically ill patient"/ OR ("critical care" OR "critical care" OR "intensive care" OR "Critical Illness" OR "Critical Illness" OR "critically ill") OR (("profound" OR "profound*").ti,ab AND (exp *"Disabled Person"/ OR exp *"Intellectual Impairment"/)) OR (("profound" OR "profound*") AND ("intellectual" OR "intellectualism" OR "intellectually" OR "intellectuals") AND ("multiple" OR "multiples") AND ("disabilities" OR "disability" OR "disabled persons" OR "disabled persons" OR "disabled" OR "disablement" OR "disablements" OR "disabling" OR "disablity")).ti,ab OR exp *"Bedridden Patient"/ OR ("Bedridden" OR "Non-Mobile Person" OR "Non-Mobile Persons").ti,ab) AND (("activity monitor" OR "activity monitors" OR "activity monitoring" OR "activity tracker" OR "activity trackers" OR "activity tracking" OR "Ambulatory Monitor*" OR "Ambulatory Monitoring" OR "Electronic Skin").ti. OR (("monitor*" OR "monitoring").ti. AND (exp *"Telemedicine"/ OR "telemed*".ti. OR "telehealth*".ti. OR "technol*".ti.)) OR "monitoring app".ti. OR "monitoring application".ti. OR "monitoring applications".ti. OR "monitoring apps".ti. OR "monitoring device".ti. OR "monitoring devices".ti. OR "monitoring technologies".ti. OR "monitoring technology".ti. OR exp *"Monitoring, Ambulatory"/ OR exp *"Monitoring, Physiologic"/ OR exp *"Ambulatory Monitoring"/ OR exp *"Physiologic Monitoring"/ OR "Noninvasive device".ti. OR "Non-invasive device".ti. OR "Noninvasive devices".ti. OR "Non-invasive devices".ti. OR "Noninvasive monitoring device".ti. OR "Noninvasive monitoring device".ti. OR "Non-invasive monitoring device".ti. OR "Noninvasive monitoring devices".ti. OR "Noninvasive monitoring devices".ti. OR "Non-invasive monitoring devices".ti. OR "Noninvasive monitoring technologies".ti. OR "Noninvasive monitoring technologies".ti. OR "Non-invasive monitoring technologies".ti. OR "Noninvasive monitoring technology".ti. OR "Noninvasive monitoring technology".ti. OR "Non-invasive monitoring technology".ti. OR "Noninvasive monitoring".ti. OR "Non-invasive monitoring".ti. OR "Noninvasive technologies".ti. OR "Non-invasive technologies".ti. OR "Noninvasive technology".ti. OR "Non-invasive technology".ti. OR "Outpatient Monitor*".ti. OR "Outpatient Monitoring".ti. OR "Portable Electronic Application".ti. OR "Portable Electronic Applications".ti. OR "Portable Software Application".ti. OR "Portable Software Applications".ti. OR "Smart watch".ti. OR "Smart watches".ti. OR "Smartwatch".ti. OR "Smartwatches".ti. OR "tracker".ti. OR "trackers".ti. OR "wearab*".ti. OR "wearable activity tracker".ti. OR "wearable activity trackers".ti. OR "wearable activity tracking".ti. OR "Wearable Device".ti. OR "Wearable Devices".ti. OR "Wearable Electronic Device".ti. OR exp *"Wearable Electronic Devices"/ OR exp *"wearable computer"/ OR "Wearable Electronic Devices".ti. OR "Wearable Technologies".ti. OR "Wearable Technology".ti. OR "wearable".ti. OR "wearables".ti. OR "noninvasive body sensor".ti. OR "noninvasive body sensors".ti. OR "non invasive body sensor".ti. OR "non invasive body sensors".ti. OR "body sensor".ti. OR "body sensors".ti. OR "wristband sensor".ti. OR "wristband sensors".ti. OR "wearable computing".ti. OR "wearable computer".ti. OR "wearable computers".ti. OR "point-of-care App".ti. OR "point-of-care Apps".ti. OR "point-of-care Application".ti. OR "point-of-care Applications".ti. OR "facial recognition technology".ti. OR "face recognition technology".ti. OR "Point-of-Care Technology".ti. OR "Point-of-Care Techn*".ti. OR "HUME".ti. OR "ePAT".ti. OR " electronic pain assessment".ti. OR ("BIS" AND "bispectral").ti. OR "bispectral index".ti. OR exp *"Consciousness Monitor"/ OR exp *"Consciousness Monitors"/ OR exp *"Biosensing Techniques"/ OR (exp *"Facial Expression"/ AND (exp *"Point-of-Care Systems"/ OR exp *"Point of Care System"/)) OR ((("noninvasiv*" OR "non invasiv*" OR "unobtrusiv*" OR "portable").ti. OR exp "Mobile Applications"/ OR exp *"Mobile Application"/) AND "monitor*".ti.) OR "Painchek".mp OR "Paincheck".mp OR "automated facial analysis".mp OR "automated facial recognition".mp OR "automated facial expression analysis".mp) AND (("discomfort" OR "discomfort*").ti. OR exp *"Psychological Distress"/ OR exp *"distress syndrome"/ OR "distressing symptoms".ti. OR "distressing symptom".ti. OR "distressing".ti. OR "distress".ti. OR exp *"Patient Comfort"/ OR "comfort".ti. OR "comfort*".ti. OR exp *"Psychomotor Agitation"/ OR exp *"Restlessness"/ OR "agitation".ti. OR "agitat*".ti. OR "restlessness".ti. OR "restless*".ti. OR "hyperactivity".ti. OR "hyperactiv*".ti. OR "akathisia".ti. OR exp *"Pain"/ OR "pain".ti. OR exp *"Pain Measurement"/ OR "pain assessment".ti. OR exp *"Stress, Psychological"/ OR exp *"Mental stress"/ OR "stress level".ti. OR "stress levels".ti. OR "pressure ulcer".ti. OR "pressure ulcer".ti. OR "life quality".ti. OR exp *"quality of life"/ OR "quality of life".ti. OR "breathing difficulty".ti. OR "difficulty breathing".ti. OR "difficult breathing".ti. OR "labored breathing".ti. OR "laboured breathing".ti. OR "Shortness of Breath".ti. OR "Breath Shortness".ti. OR "Breathlessness".ti. OR exp *"Dyspnea"/ OR "Dyspnea".ti. OR exp *"Anxiety"/ OR "Anxiety".ti. OR "Nervousness".ti. OR "Hypervigilance".ti. OR "Anxiousness".ti. OR exp *"Sleep Disorder"/ OR exp *"Sleep Wake Disorders"/ OR "sleep disturbances".ti. OR "sleep disturbance".ti. OR exp *"Fatigue"/ OR "fatigue".ti. OR exp *"Nausea"/ OR "nausea".ti. OR "nauseous".ti. OR exp *"Delirium"/ OR "delirium".ti. OR "delirious".ti. OR exp *"Fever"/ OR "fever".ti. OR exp *"Urinary Incontinence"/ OR exp *"Urine Incontinence"/ OR "Incontinence".ti. OR "incontinen*".ti. OR "Sedation depth".ti. OR "Sedation depths".ti. OR "Depth of sedation".ti. OR exp *"anesthesia level"/))

## Web of Science

(TS=("Dementia" OR "Dementia" OR "dement*" OR "Alzheimer Disease" OR "Alzheimer*" OR "CADASIL" OR "Creutzfeldt-Jakob" OR "Creutzfeldt-Jakob Syndrome" OR "Diffuse Neurofibrillary Tangles with Calcification" OR "Frontotemporal Lobar Degeneration" OR "Huntington Disease" OR "Huntington*" OR "Kluver-Bucy" OR "Kluver-Bucy Syndrome" OR "Lewy Body" OR "Lewy Body Disease" OR "Pick Disease of the Brain" OR "Primary Progressive Aphasia" OR "Primary Progressive Nonfluent Aphasia" OR "Death" OR "dying" OR "end of life phase" OR "end of life" OR "end-of-life phase" OR "endoflife" OR "end-of-life" OR "Hospice and Palliative Care Nursing" OR "Hospice Care" OR "Hospice Care" OR "Hospice Care" OR "Hospice" OR "Hospices" OR "Hospices" OR "palliat*" OR "Palliative Care" OR "Palliative Care" OR "Palliative Medicine" OR "Palliative Phase" OR "Palliative Phases" OR "Palliative Stage" OR "Palliative Stages" OR "Palliative Supportive Care" OR "Palliative Surgery" OR "Palliative Therapy" OR "Palliative Treatment" OR "Palliative Treatments" OR "Terminal Care" OR "Terminal Care" OR "terminal stage" OR "elderly" OR "critical care" OR "critical care" OR "intensive care" OR "Critical Illness" OR "Critical Illness" OR "critically ill" OR (("profound" OR "profound*") AND ("Disabled Persons" OR "Intellectual Disability")) OR (("profound" OR "profound*") AND ("intellectual" OR "intellectualism" OR "intellectually" OR "intellectuals") AND ("multiple" OR "multiples") AND ("disabilities" OR "disability" OR "disabled persons" OR "disabled persons" OR "disabled" OR "disablement" OR "disablements" OR "disabling" OR "disablity")) OR "Bedridden Persons" OR "Bedridden" OR "Non-Mobile Person" OR "Non-Mobile Persons") AND TS=("activity monitor" OR "activity monitors" OR "activity monitoring" OR "activity tracker" OR "activity trackers" OR "activity tracking" OR "Ambulatory Monitor*" OR "Ambulatory Monitoring" OR "Electronic Skin" OR (("monitor*" OR "monitoring") AND ("Telemedicine" OR "telemed*" OR "telehealth*" OR "technol*")) OR "monitoring app" OR "monitoring application" OR "monitoring applications" OR "monitoring apps" OR "monitoring device" OR "monitoring devices" OR "monitoring technologies" OR "monitoring technology" OR "Monitoring, Ambulatory" OR "Monitoring, Physiologic" OR "Noninvasive device" OR "Non-invasive device" OR "Noninvasive devices" OR "Non-invasive devices" OR "Noninvasive monitoring device" OR "Noninvasive monitoring device" OR "Non-invasive monitoring device" OR "Noninvasive monitoring devices" OR "Noninvasive monitoring devices" OR "Non-invasive monitoring devices" OR "Noninvasive monitoring technologies" OR "Noninvasive monitoring technologies" OR "Non-invasive monitoring technologies" OR "Noninvasive monitoring technology" OR "Noninvasive monitoring technology" OR "Non-invasive monitoring technology" OR "Noninvasive monitoring" OR "Non-invasive monitoring" OR "Noninvasive technologies" OR "Non-invasive technologies" OR "Noninvasive technology" OR "Non-invasive technology" OR "Outpatient Monitor*" OR "Outpatient Monitoring" OR "Portable Electronic Application" OR "Portable Electronic Applications" OR "Portable Software Application" OR "Portable Software Applications" OR "Smart watch" OR "Smart watches" OR "Smartwatch" OR "Smartwatches" OR "tracker" OR "trackers" OR "wearab*" OR "wearable activity tracker" OR "wearable activity trackers" OR "wearable activity tracking" OR "Wearable Device" OR "Wearable Devices" OR "Wearable Electronic Device" OR "Wearable Electronic Devices" OR "Wearable Electronic Devices" OR "Wearable Technologies" OR "Wearable Technology" OR "wearable" OR "wearables" OR "noninvasive body sensor" OR "noninvasive body sensors" OR "non invasive body sensor" OR "non invasive body sensors" OR "body sensor" OR "body sensors" OR "wristband sensor" OR "wristband sensors" OR "wearable computing" OR "wearable computer" OR "wearable computers" OR "point-of-care App" OR "point-of-care Apps" OR "point-of-care Application" OR "point-of-care Applications" OR "facial recognition technology" OR "face recognition technology" OR "Point-of-Care Technology" OR "Point-of-Care Techn*" OR "HUME" OR "ePAT" OR " electronic pain assessment" OR ("BIS" AND "bispectral") OR "bispectral index" OR "Consciousness Monitors" OR "Biosensing Techniques" OR ("Facial Expression" AND "Point-of-Care Systems") OR (("noninvasiv*" OR "non invasiv*" OR "unobtrusiv*" OR "portable" OR "Mobile Applications") AND "monitor*") OR "Painchek" OR "Paincheck" OR "automated facial analysis" OR "automated facial recognition" OR "automated facial expression analysis") AND TI=("discomfort" OR "discomfort*" OR "Psychological Distress" OR "distressing symptoms" OR "distressing symptom" OR "distressing" OR "distress" OR "Patient Comfort" OR "comfort" OR "comfort*" OR "Psychomotor Agitation" OR "agitation" OR "agitat*" OR "restlessness" OR "restless*" OR "hyperactivity" OR "hyperactiv*" OR "akathisia" OR "Pain" OR "pain" OR "Pain Measurement" OR "pain assessment" OR "Stress, Psychological" OR "stress level" OR "stress levels" OR "pressure ulcer" OR "pressure ulcer" OR "life quality" OR "quality of life" OR "quality of life" OR "breathing difficulty" OR "difficulty breathing" OR "difficult breathing" OR "labored breathing" OR "laboured breathing" OR "Shortness of Breath" OR "Breath Shortness" OR "Breathlessness" OR "Dyspnea" OR "Dyspnea" OR "Anxiety" OR "Anxiety" OR "Nervousness" OR "Hypervigilance" OR "Anxiousness" OR "Sleep Wake Disorders" OR "sleep disturbances" OR "sleep disturbance" OR "Fatigue" OR "fatigue" OR "Nausea" OR "nausea" OR "nauseous" OR "Delirium" OR "delirium" OR "delirious" OR "Fever" OR "fever" OR "Urinary Incontinence" OR "Incontinence" OR "incontinen*" OR "Sedation depth" OR "Sedation depths" OR "Depth of sedation"))

## Cochrane Library

(("Dementia" OR "Dementia" OR "dement*" OR "Alzheimer Disease" OR "Alzheimer*" OR "CADASIL" OR "Creutzfeldt Jakob" OR "Creutzfeldt Jakob Syndrome" OR "Diffuse Neurofibrillary Tangles with Calcification" OR "Frontotemporal Lobar Degeneration" OR "Huntington Disease" OR "Huntington*" OR "Kluver Bucy" OR "Kluver Bucy Syndrome" OR "Lewy Body" OR "Lewy Body Disease" OR "Pick Disease of the Brain" OR "Primary Progressive Aphasia" OR "Primary Progressive Nonfluent Aphasia" OR "Death" OR "dying" OR "end of life phase" OR "end of life" OR "end of life phase" OR "endoflife" OR "end of life" OR "Hospice and Palliative Care Nursing" OR "Hospice Care" OR "Hospice Care" OR "Hospice Care" OR "Hospice" OR "Hospices" OR "Hospices" OR "palliat*" OR "Palliative Care" OR "Palliative Care" OR "Palliative Medicine" OR "Palliative Phase" OR "Palliative Phases" OR "Palliative Stage" OR "Palliative Stages" OR "Palliative Supportive Care" OR "Palliative Surgery" OR "Palliative Therapy" OR "Palliative Treatment" OR "Palliative Treatments" OR "Terminal Care" OR "Terminal Care" OR "terminal stage" OR "elderly" OR "critical care" OR "critical care" OR "intensive care" OR "Critical Illness" OR "Critical Illness" OR "critically ill" OR (("profound" OR "profound*") AND ("Disabled Persons" OR "Intellectual Disability")) OR (("profound" OR "profound*") AND ("intellectual" OR "intellectualism" OR "intellectually" OR "intellectuals") AND ("multiple" OR "multiples") AND ("disabilities" OR "disability" OR "disabled persons" OR "disabled persons" OR "disabled" OR "disablement" OR "disablements" OR "disabling" OR "disablity")) OR "Bedridden Persons" OR "Bedridden" OR "Non Mobile Person" OR "Non Mobile Persons"):ti,ab,kw AND ("activity monitor" OR "activity monitors" OR "activity monitoring" OR "activity tracker" OR "activity trackers" OR "activity tracking" OR "Ambulatory Monitor*" OR "Ambulatory Monitoring" OR "Electronic Skin" OR (("monitor*" OR "monitoring") AND ("Telemedicine" OR "telemed*" OR "telehealth*" OR "technol*")) OR "monitoring app" OR "monitoring application" OR "monitoring applications" OR "monitoring apps" OR "monitoring device" OR "monitoring devices" OR "monitoring technologies" OR "monitoring technology" OR "Monitoring, Ambulatory" OR "Monitoring, Physiologic" OR "Noninvasive device" OR "Non invasive device" OR "Noninvasive devices" OR "Non invasive devices" OR "Noninvasive monitoring device" OR "Noninvasive monitoring device" OR "Non invasive monitoring device" OR "Noninvasive monitoring devices" OR "Noninvasive monitoring devices" OR "Non invasive monitoring devices" OR "Noninvasive monitoring technologies" OR "Noninvasive monitoring technologies" OR "Non invasive monitoring technologies" OR "Noninvasive monitoring technology" OR "Noninvasive monitoring technology" OR "Non invasive monitoring technology" OR "Noninvasive monitoring" OR "Non invasive monitoring" OR "Noninvasive technologies" OR "Non invasive technologies" OR "Noninvasive technology" OR "Non invasive technology" OR "Outpatient Monitor*" OR "Outpatient Monitoring" OR "Portable Electronic Application" OR "Portable Electronic Applications" OR "Portable Software Application" OR "Portable Software Applications" OR "Smart watch" OR "Smart watches" OR "Smartwatch" OR "Smartwatches" OR "tracker" OR "trackers" OR "wearab*" OR "wearable activity tracker" OR "wearable activity trackers" OR "wearable activity tracking" OR "Wearable Device" OR "Wearable Devices" OR "Wearable Electronic Device" OR "Wearable Electronic Devices" OR "Wearable Electronic Devices" OR "Wearable Technologies" OR "Wearable Technology" OR "wearable" OR "wearables" OR "noninvasive body sensor" OR "noninvasive body sensors" OR "non invasive body sensor" OR "non invasive body sensors" OR "body sensor" OR "body sensors" OR "wristband sensor" OR "wristband sensors" OR "wearable computing" OR "wearable computer" OR "wearable computers" OR "point of care App" OR "point of care Apps" OR "point of care Application" OR "point of care Applications" OR "facial recognition technology" OR "face recognition technology" OR "Point of Care Technology" OR "Point of Care Techn*" OR "HUME" OR "ePAT" OR " electronic pain assessment" OR ("BIS" AND "bispectral") OR "bispectral index" OR "Consciousness Monitors" OR "Biosensing Techniques" OR ("Facial Expression" AND "Point of Care Systems") OR (("noninvasiv*" OR "non invasiv*" OR "unobtrusiv*" OR "portable" OR "Mobile Applications") AND "monitor*") OR "Painchek" OR "Paincheck" OR "automated facial analysis" OR "automated facial recognition" OR "automated facial expression analysis"):ti AND ("discomfort" OR "discomfort*" OR "Psychological Distress" OR "distressing symptoms" OR "distressing symptom" OR "distressing" OR "distress" OR "Patient Comfort" OR "comfort" OR "comfort*" OR "Psychomotor Agitation" OR "agitation" OR "agitat*" OR "restlessness" OR "restless*" OR "hyperactivity" OR "hyperactiv*" OR "akathisia" OR "Pain" OR "pain" OR "Pain Measurement" OR "pain assessment" OR "Stress, Psychological" OR "stress level" OR "stress levels" OR "pressure ulcer" OR "pressure ulcer" OR "life quality" OR "quality of life" OR "quality of life" OR "breathing difficulty" OR "difficulty breathing" OR "difficult breathing" OR "labored breathing" OR "laboured breathing" OR "Shortness of Breath" OR "Breath Shortness" OR "Breathlessness" OR "Dyspnea" OR "Dyspnea" OR "Anxiety" OR "Anxiety" OR "Nervousness" OR "Hypervigilance" OR "Anxiousness" OR "Sleep Wake Disorders" OR "sleep disturbances" OR "sleep disturbance" OR "Fatigue" OR "fatigue" OR "Nausea" OR "nausea" OR "nauseous" OR "Delirium" OR "delirium" OR "delirious" OR "Fever" OR "fever" OR "Urinary Incontinence" OR "Incontinence" OR "incontinen*" OR "Sedation depth" OR "Sedation depths" OR "Depth of sedation"):ti,ab,kw) OR (("Dementia" OR "Dementia" OR "dement*" OR "Alzheimer Disease" OR "Alzheimer*" OR "CADASIL" OR "Creutzfeldt Jakob" OR "Creutzfeldt Jakob Syndrome" OR "Diffuse Neurofibrillary Tangles with Calcification" OR "Frontotemporal Lobar Degeneration" OR "Huntington Disease" OR "Huntington*" OR "Kluver Bucy" OR "Kluver Bucy Syndrome" OR "Lewy Body" OR "Lewy Body Disease" OR "Pick Disease of the Brain" OR "Primary Progressive Aphasia" OR "Primary Progressive Nonfluent Aphasia" OR "Death" OR "dying" OR "end of life phase" OR "end of life" OR "end of life phase" OR "endoflife" OR "end of life" OR "Hospice and Palliative Care Nursing" OR "Hospice Care" OR "Hospice Care" OR "Hospice Care" OR "Hospice" OR "Hospices" OR "Hospices" OR "palliat*" OR "Palliative Care" OR "Palliative Care" OR "Palliative Medicine" OR "Palliative Phase" OR "Palliative Phases" OR "Palliative Stage" OR "Palliative Stages" OR "Palliative Supportive Care" OR "Palliative Surgery" OR "Palliative Therapy" OR "Palliative Treatment" OR "Palliative Treatments" OR "Terminal Care" OR "Terminal Care" OR "terminal stage" OR "elderly" OR "critical care" OR "critical care" OR "intensive care" OR "Critical Illness" OR "Critical Illness" OR "critically ill" OR (("profound" OR "profound*") AND ("Disabled Persons" OR "Intellectual Disability")) OR (("profound" OR "profound*") AND ("intellectual" OR "intellectualism" OR "intellectually" OR "intellectuals") AND ("multiple" OR "multiples") AND ("disabilities" OR "disability" OR "disabled persons" OR "disabled persons" OR "disabled" OR "disablement" OR "disablements" OR "disabling" OR "disablity")) OR "Bedridden Persons" OR "Bedridden" OR "Non Mobile Person" OR "Non Mobile Persons"):ti,ab,kw AND ("activity monitor" OR "activity monitors" OR "activity monitoring" OR "activity tracker" OR "activity trackers" OR "activity tracking" OR "Ambulatory Monitor*" OR "Ambulatory Monitoring" OR "Electronic Skin" OR (("monitor*" OR "monitoring") AND ("Telemedicine" OR "telemed*" OR "telehealth*" OR "technol*")) OR "monitoring app" OR "monitoring application" OR "monitoring applications" OR "monitoring apps" OR "monitoring device" OR "monitoring devices" OR "monitoring technologies" OR "monitoring technology" OR "Monitoring, Ambulatory" OR "Monitoring, Physiologic" OR "Noninvasive device" OR "Non invasive device" OR "Noninvasive devices" OR "Non invasive devices" OR "Noninvasive monitoring device" OR "Noninvasive monitoring device" OR "Non invasive monitoring device" OR "Noninvasive monitoring devices" OR "Noninvasive monitoring devices" OR "Non invasive monitoring devices" OR "Noninvasive monitoring technologies" OR "Noninvasive monitoring technologies" OR "Non invasive monitoring technologies" OR "Noninvasive monitoring technology" OR "Noninvasive monitoring technology" OR "Non invasive monitoring technology" OR "Noninvasive monitoring" OR "Non invasive monitoring" OR "Noninvasive technologies" OR "Non invasive technologies" OR "Noninvasive technology" OR "Non invasive technology" OR "Outpatient Monitor*" OR "Outpatient Monitoring" OR "Portable Electronic Application" OR "Portable Electronic Applications" OR "Portable Software Application" OR "Portable Software Applications" OR "Smart watch" OR "Smart watches" OR "Smartwatch" OR "Smartwatches" OR "tracker" OR "trackers" OR "wearab*" OR "wearable activity tracker" OR "wearable activity trackers" OR "wearable activity tracking" OR "Wearable Device" OR "Wearable Devices" OR "Wearable Electronic Device" OR "Wearable Electronic Devices" OR "Wearable Electronic Devices" OR "Wearable Technologies" OR "Wearable Technology" OR "wearable" OR "wearables" OR "noninvasive body sensor" OR "noninvasive body sensors" OR "non invasive body sensor" OR "non invasive body sensors" OR "body sensor" OR "body sensors" OR "wristband sensor" OR "wristband sensors" OR "wearable computing" OR "wearable computer" OR "wearable computers" OR "point of care App" OR "point of care Apps" OR "point of care Application" OR "point of care Applications" OR "facial recognition technology" OR "face recognition technology" OR "Point of Care Technology" OR "Point of Care Techn*" OR "HUME" OR "ePAT" OR " electronic pain assessment" OR ("BIS" AND "bispectral") OR "bispectral index" OR "Consciousness Monitors" OR "Biosensing Techniques" OR ("Facial Expression" AND "Point of Care Systems") OR (("noninvasiv*" OR "non invasiv*" OR "unobtrusiv*" OR "portable" OR "Mobile Applications") AND "monitor*") OR "Painchek" OR "Paincheck" OR "automated facial analysis" OR "automated facial recognition" OR "automated facial expression analysis"):ti,ab,kw AND ("discomfort" OR "discomfort*" OR "Psychological Distress" OR "distressing symptoms" OR "distressing symptom" OR "distressing" OR "distress" OR "Patient Comfort" OR "comfort" OR "comfort*" OR "Psychomotor Agitation" OR "agitation" OR "agitat*" OR "restlessness" OR "restless*" OR "hyperactivity" OR "hyperactiv*" OR "akathisia" OR "Pain" OR "pain" OR "Pain Measurement" OR "pain assessment" OR "Stress, Psychological" OR "stress level" OR "stress levels" OR "pressure ulcer" OR "pressure ulcer" OR "life quality" OR "quality of life" OR "quality of life" OR "breathing difficulty" OR "difficulty breathing" OR "difficult breathing" OR "labored breathing" OR "laboured breathing" OR "Shortness of Breath" OR "Breath Shortness" OR "Breathlessness" OR "Dyspnea" OR "Dyspnea" OR "Anxiety" OR "Anxiety" OR "Nervousness" OR "Hypervigilance" OR "Anxiousness" OR "Sleep Wake Disorders" OR "sleep disturbances" OR "sleep disturbance" OR "Fatigue" OR "fatigue" OR "Nausea" OR "nausea" OR "nauseous" OR "Delirium" OR "delirium" OR "delirious" OR "Fever" OR "fever" OR "Urinary Incontinence" OR "Incontinence" OR "incontinen*" OR "Sedation depth" OR "Sedation depths" OR "Depth of sedation"):ti)

## Emcare (OVID)

((exp *"Dementia"/ OR ("Dementia" OR "dement*" OR "Alzheimer Disease" OR "Alzheimer*" OR "CADASIL" OR "Creutzfeldt-Jakob" OR "Creutzfeldt-Jakob Syndrome" OR "Diffuse Neurofibrillary Tangles with Calcification" OR "Frontotemporal Lobar Degeneration" OR "Huntington Disease" OR "Huntington*" OR "Kluver-Bucy" OR "Kluver-Bucy Syndrome" OR "Lewy Body" OR "Lewy Body Disease" OR "Pick Disease of the Brain" OR "Primary Progressive Aphasia" OR "Primary Progressive Nonfluent Aphasia").ti,ab OR exp *"Death"/ OR exp *"Terminal Care"/ OR exp *"Hospice"/ OR exp *"Palliative Therapy"/ OR ("dying" OR "end of life phase" OR "end of life" OR "end-of-life phase" OR "endoflife" OR "end-of-life" OR "Hospice and Palliative Care Nursing" OR "Hospice Care" OR "Hospice Care" OR "Hospice Care" OR "Hospice" OR "Hospices" OR "Hospices" OR "palliat*" OR "Palliative Care" OR "Palliative Care" OR "Palliative Medicine" OR "Palliative Phase" OR "Palliative Phases" OR "Palliative Stage" OR "Palliative Stages" OR "Palliative Supportive Care" OR "Palliative Surgery" OR "Palliative Therapy" OR "Palliative Treatment" OR "Palliative Treatments" OR "Terminal Care" OR "Terminal Care" OR "terminal stage").ti,ab OR "elderly".ti OR exp *"Intensive Care"/ OR exp *"Critical Illness"/ OR exp *"critically ill patient"/ OR ("critical care" OR "critical care" OR "intensive care" OR "Critical Illness" OR "Critical Illness" OR "critically ill") OR (("profound" OR "profound*").ti,ab AND (exp *"Disabled Person"/ OR exp *"Intellectual Impairment"/)) OR (("profound" OR "profound*") AND ("intellectual" OR "intellectualism" OR "intellectually" OR "intellectuals") AND ("multiple" OR "multiples") AND ("disabilities" OR "disability" OR "disabled persons" OR "disabled persons" OR "disabled" OR "disablement" OR "disablements" OR "disabling" OR "disablity")).ti,ab OR exp *"Bedridden Patient"/ OR ("Bedridden" OR "Non-Mobile Person" OR "Non-Mobile Persons").ti,ab) AND (("activity monitor" OR "activity monitors" OR "activity monitoring" OR "activity tracker" OR "activity trackers" OR "activity tracking" OR "Ambulatory Monitor*" OR "Ambulatory Monitoring" OR "Electronic Skin").ti. OR (("monitor*" OR "monitoring").ti. AND (exp *"Telemedicine"/ OR "telemed*".ti. OR "telehealth*".ti. OR "technol*".ti.)) OR "monitoring app".ti. OR "monitoring application".ti. OR "monitoring applications".ti. OR "monitoring apps".ti. OR "monitoring device".ti. OR "monitoring devices".ti. OR "monitoring technologies".ti. OR "monitoring technology".ti. OR exp *"Monitoring, Ambulatory"/ OR exp *"Monitoring, Physiologic"/ OR exp *"Ambulatory Monitoring"/ OR exp *"Physiologic Monitoring"/ OR "Noninvasive device".ti. OR "Non-invasive device".ti. OR "Noninvasive devices".ti. OR "Non-invasive devices".ti. OR "Noninvasive monitoring device".ti. OR "Noninvasive monitoring device".ti. OR "Non-invasive monitoring device".ti. OR "Noninvasive monitoring devices".ti. OR "Noninvasive monitoring devices".ti. OR "Non-invasive monitoring devices".ti. OR "Noninvasive monitoring technologies".ti. OR "Noninvasive monitoring technologies".ti. OR "Non-invasive monitoring technologies".ti. OR "Noninvasive monitoring technology".ti. OR "Noninvasive monitoring technology".ti. OR "Non-invasive monitoring technology".ti. OR "Noninvasive monitoring".ti. OR "Non-invasive monitoring".ti. OR "Noninvasive technologies".ti. OR "Non-invasive technologies".ti. OR "Noninvasive technology".ti. OR "Non-invasive technology".ti. OR "Outpatient Monitor*".ti. OR "Outpatient Monitoring".ti. OR "Portable Electronic Application".ti. OR "Portable Electronic Applications".ti. OR "Portable Software Application".ti. OR "Portable Software Applications".ti. OR "Smart watch".ti. OR "Smart watches".ti. OR "Smartwatch".ti. OR "Smartwatches".ti. OR "tracker".ti. OR "trackers".ti. OR "wearab*".ti. OR "wearable activity tracker".ti. OR "wearable activity trackers".ti. OR "wearable activity tracking".ti. OR "Wearable Device".ti. OR "Wearable Devices".ti. OR "Wearable Electronic Device".ti. OR exp *"Wearable Electronic Devices"/ OR exp *"wearable computer"/ OR "Wearable Electronic Devices".ti. OR "Wearable Technologies".ti. OR "Wearable Technology".ti. OR "wearable".ti. OR "wearables".ti. OR "noninvasive body sensor".ti. OR "noninvasive body sensors".ti. OR "non invasive body sensor".ti. OR "non invasive body sensors".ti. OR "body sensor".ti. OR "body sensors".ti. OR "wristband sensor".ti. OR "wristband sensors".ti. OR "wearable computing".ti. OR "wearable computer".ti. OR "wearable computers".ti. OR "point-of-care App".ti. OR "point-of-care Apps".ti. OR "point-of-care Application".ti. OR "point-of-care Applications".ti. OR "facial recognition technology".ti. OR "face recognition technology".ti. OR "Point-of-Care Technology".ti. OR "Point-of-Care Techn*".ti. OR "HUME".ti. OR "ePAT".ti. OR " electronic pain assessment".ti. OR ("BIS" AND "bispectral").ti. OR "bispectral index".ti. OR exp *"Consciousness Monitor"/ OR exp *"Consciousness Monitors"/ OR exp *"Biosensing Techniques"/ OR (exp *"Facial Expression"/ AND (exp *"Point-of-Care Systems"/ OR exp *"Point of Care System"/)) OR ((("noninvasiv*" OR "non invasiv*" OR "unobtrusiv*" OR "portable").ti. OR exp "Mobile Applications"/ OR exp *"Mobile Application"/) AND "monitor*".ti.) OR "Painchek".mp OR "Paincheck".mp OR "automated facial analysis".mp OR "automated facial recognition".mp OR "automated facial expression analysis".mp) AND (("discomfort" OR "discomfort*").ti. OR exp *"Psychological Distress"/ OR exp *"distress syndrome"/ OR "distressing symptoms".ti. OR "distressing symptom".ti. OR "distressing".ti. OR "distress".ti. OR exp *"Patient Comfort"/ OR "comfort".ti. OR "comfort*".ti. OR exp *"Psychomotor Agitation"/ OR exp *"Restlessness"/ OR "agitation".ti. OR "agitat*".ti. OR "restlessness".ti. OR "restless*".ti. OR "hyperactivity".ti. OR "hyperactiv*".ti. OR "akathisia".ti. OR exp *"Pain"/ OR "pain".ti. OR exp *"Pain Measurement"/ OR "pain assessment".ti. OR exp *"Stress, Psychological"/ OR exp *"Mental stress"/ OR "stress level".ti. OR "stress levels".ti. OR "pressure ulcer".ti. OR "pressure ulcer".ti. OR "life quality".ti. OR exp *"quality of life"/ OR "quality of life".ti. OR "breathing difficulty".ti. OR "difficulty breathing".ti. OR "difficult breathing".ti. OR "labored breathing".ti. OR "laboured breathing".ti. OR "Shortness of Breath".ti. OR "Breath Shortness".ti. OR "Breathlessness".ti. OR exp *"Dyspnea"/ OR "Dyspnea".ti. OR exp *"Anxiety"/ OR "Anxiety".ti. OR "Nervousness".ti. OR "Hypervigilance".ti. OR "Anxiousness".ti. OR exp *"Sleep Disorder"/ OR exp *"Sleep Wake Disorders"/ OR "sleep disturbances".ti. OR "sleep disturbance".ti. OR exp *"Fatigue"/ OR "fatigue".ti. OR exp *"Nausea"/ OR "nausea".ti. OR "nauseous".ti. OR exp *"Delirium"/ OR "delirium".ti. OR "delirious".ti. OR exp *"Fever"/ OR "fever".ti. OR exp *"Urinary Incontinence"/ OR exp *"Urine Incontinence"/ OR "Incontinence".ti. OR "incontinen*".ti. OR "Sedation depth".ti. OR "Sedation depths".ti. OR "Depth of sedation".ti. OR exp *"anesthesia level"/))

## PsycINFO (EbscoHOST)

(TX("Dementia" OR "Dementia" OR "dement*" OR "Alzheimer Disease" OR "Alzheimer*" OR "CADASIL" OR "Creutzfeldt-Jakob" OR "Creutzfeldt-Jakob Syndrome" OR "Diffuse Neurofibrillary Tangles with Calcification" OR "Frontotemporal Lobar Degeneration" OR "Huntington Disease" OR "Huntington*" OR "Kluver-Bucy" OR "Kluver-Bucy Syndrome" OR "Lewy Body" OR "Lewy Body Disease" OR "Pick Disease of the Brain" OR "Primary Progressive Aphasia" OR "Primary Progressive Nonfluent Aphasia" OR "Death" OR "dying" OR "end of life phase" OR "end of life" OR "end-of-life phase" OR "endoflife" OR "end-of-life" OR "Hospice and Palliative Care Nursing" OR "Hospice Care" OR "Hospice Care" OR "Hospice Care" OR "Hospice" OR "Hospices" OR "Hospices" OR "palliat*" OR "Palliative Care" OR "Palliative Care" OR "Palliative Medicine" OR "Palliative Phase" OR "Palliative Phases" OR "Palliative Stage" OR "Palliative Stages" OR "Palliative Supportive Care" OR "Palliative Surgery" OR "Palliative Therapy" OR "Palliative Treatment" OR "Palliative Treatments" OR "Terminal Care" OR "Terminal Care" OR "terminal stage" OR "elderly" OR "critical care" OR "critical care" OR "intensive care" OR "Critical Illness" OR "Critical Illness" OR "critically ill" OR (("profound" OR "profound*") AND ("Disabled Persons" OR "Intellectual Disability")) OR (("profound" OR "profound*") AND ("intellectual" OR "intellectualism" OR "intellectually" OR "intellectuals") AND ("multiple" OR "multiples") AND ("disabilities" OR "disability" OR "disabled persons" OR "disabled persons" OR "disabled" OR "disablement" OR "disablements" OR "disabling" OR "disablity")) OR "Bedridden Persons" OR "Bedridden" OR "Non-Mobile Person" OR "Non-Mobile Persons") AND TI("activity monitor" OR "activity monitors" OR "activity monitoring" OR "activity tracker" OR "activity trackers" OR "activity tracking" OR "Ambulatory Monitor*" OR "Ambulatory Monitoring" OR "Electronic Skin" OR (("monitor*" OR "monitoring") AND ("Telemedicine" OR "telemed*" OR "telehealth*" OR "technol*")) OR "monitoring app" OR "monitoring application" OR "monitoring applications" OR "monitoring apps" OR "monitoring device" OR "monitoring devices" OR "monitoring technologies" OR "monitoring technology" OR "Monitoring, Ambulatory" OR "Monitoring, Physiologic" OR "Noninvasive device" OR "Non-invasive device" OR "Noninvasive devices" OR "Non-invasive devices" OR "Noninvasive monitoring device" OR "Noninvasive monitoring device" OR "Non-invasive monitoring device" OR "Noninvasive monitoring devices" OR "Noninvasive monitoring devices" OR "Non-invasive monitoring devices" OR "Noninvasive monitoring technologies" OR "Noninvasive monitoring technologies" OR "Non-invasive monitoring technologies" OR "Noninvasive monitoring technology" OR "Noninvasive monitoring technology" OR "Non-invasive monitoring technology" OR "Noninvasive monitoring" OR "Non-invasive monitoring" OR "Noninvasive technologies" OR "Non-invasive technologies" OR "Noninvasive technology" OR "Non-invasive technology" OR "Outpatient Monitor*" OR "Outpatient Monitoring" OR "Portable Electronic Application" OR "Portable Electronic Applications" OR "Portable Software Application" OR "Portable Software Applications" OR "Smart watch" OR "Smart watches" OR "Smartwatch" OR "Smartwatches" OR "tracker" OR "trackers" OR "wearab*" OR "wearable activity tracker" OR "wearable activity trackers" OR "wearable activity tracking" OR "Wearable Device" OR "Wearable Devices" OR "Wearable Electronic Device" OR "Wearable Electronic Devices" OR "Wearable Electronic Devices" OR "Wearable Technologies" OR "Wearable Technology" OR "wearable" OR "wearables" OR "noninvasive body sensor" OR "noninvasive body sensors" OR "non invasive body sensor" OR "non invasive body sensors" OR "body sensor" OR "body sensors" OR "wristband sensor" OR "wristband sensors" OR "wearable computing" OR "wearable computer" OR "wearable computers" OR "point-of-care App" OR "point-of-care Apps" OR "point-of-care Application" OR "point-of-care Applications" OR "facial recognition technology" OR "face recognition technology" OR "Point-of-Care Technology" OR "Point-of-Care Techn*" OR "HUME" OR "ePAT" OR " electronic pain assessment" OR ("BIS" AND "bispectral") OR "bispectral index" OR "Consciousness Monitors" OR "Biosensing Techniques" OR ("Facial Expression" AND "Point-of-Care Systems") OR (("noninvasiv*" OR "non invasiv*" OR "unobtrusiv*" OR "portable" OR "Mobile Applications") AND "monitor*") OR "Painchek" OR "Paincheck" OR "automated facial analysis" OR "automated facial recognition" OR "automated facial expression analysis") AND TI("discomfort" OR "discomfort*" OR "Psychological Distress" OR "distressing symptoms" OR "distressing symptom" OR "distressing" OR "distress" OR "Patient Comfort" OR "comfort" OR "comfort*" OR "Psychomotor Agitation" OR "agitation" OR "agitat*" OR "restlessness" OR "restless*" OR "hyperactivity" OR "hyperactiv*" OR "akathisia" OR "Pain" OR "pain" OR "Pain Measurement" OR "pain assessment" OR "Stress, Psychological" OR "stress level" OR "stress levels" OR "pressure ulcer" OR "pressure ulcer" OR "life quality" OR "quality of life" OR "quality of life" OR "breathing difficulty" OR "difficulty breathing" OR "difficult breathing" OR "labored breathing" OR "laboured breathing" OR "Shortness of Breath" OR "Breath Shortness" OR "Breathlessness" OR "Dyspnea" OR "Dyspnea" OR "Anxiety" OR "Anxiety" OR "Nervousness" OR "Hypervigilance" OR "Anxiousness" OR "Sleep Wake Disorders" OR "sleep disturbances" OR "sleep disturbance" OR "Fatigue" OR "fatigue" OR "Nausea" OR "nausea" OR "nauseous" OR "Delirium" OR "delirium" OR "delirious" OR "Fever" OR "fever" OR "Urinary Incontinence" OR "Incontinence" OR "incontinen*" OR "Sedation depth" OR "Sedation depths" OR "Depth of sedation")) OR (TI("Dementia" OR "Dementia" OR "dement*" OR "Alzheimer Disease" OR "Alzheimer*" OR "CADASIL" OR "Creutzfeldt-Jakob" OR "Creutzfeldt-Jakob Syndrome" OR "Diffuse Neurofibrillary Tangles with Calcification" OR "Frontotemporal Lobar Degeneration" OR "Huntington Disease" OR "Huntington*" OR "Kluver-Bucy" OR "Kluver-Bucy Syndrome" OR "Lewy Body" OR "Lewy Body Disease" OR "Pick Disease of the Brain" OR "Primary Progressive Aphasia" OR "Primary Progressive Nonfluent Aphasia" OR "Death" OR "dying" OR "end of life phase" OR "end of life" OR "end-of-life phase" OR "endoflife" OR "end-of-life" OR "Hospice and Palliative Care Nursing" OR "Hospice Care" OR "Hospice Care" OR "Hospice Care" OR "Hospice" OR "Hospices" OR "Hospices" OR "palliat*" OR "Palliative Care" OR "Palliative Care" OR "Palliative Medicine" OR "Palliative Phase" OR "Palliative Phases" OR "Palliative Stage" OR "Palliative Stages" OR "Palliative Supportive Care" OR "Palliative Surgery" OR "Palliative Therapy" OR "Palliative Treatment" OR "Palliative Treatments" OR "Terminal Care" OR "Terminal Care" OR "terminal stage" OR "elderly" OR "critical care" OR "critical care" OR "intensive care" OR "Critical Illness" OR "Critical Illness" OR "critically ill" OR (("profound" OR "profound*") AND ("Disabled Persons" OR "Intellectual Disability")) OR (("profound" OR "profound*") AND ("intellectual" OR "intellectualism" OR "intellectually" OR "intellectuals") AND ("multiple" OR "multiples") AND ("disabilities" OR "disability" OR "disabled persons" OR "disabled persons" OR "disabled" OR "disablement" OR "disablements" OR "disabling" OR "disablity")) OR "Bedridden Persons" OR "Bedridden" OR "Non-Mobile Person" OR "Non-Mobile Persons") AND TX("activity monitor" OR "activity monitors" OR "activity monitoring" OR "activity tracker" OR "activity trackers" OR "activity tracking" OR "Ambulatory Monitor*" OR "Ambulatory Monitoring" OR "Electronic Skin" OR (("monitor*" OR "monitoring") AND ("Telemedicine" OR "telemed*" OR "telehealth*" OR "technol*")) OR "monitoring app" OR "monitoring application" OR "monitoring applications" OR "monitoring apps" OR "monitoring device" OR "monitoring devices" OR "monitoring technologies" OR "monitoring technology" OR "Monitoring, Ambulatory" OR "Monitoring, Physiologic" OR "Noninvasive device" OR "Non-invasive device" OR "Noninvasive devices" OR "Non-invasive devices" OR "Noninvasive monitoring device" OR "Noninvasive monitoring device" OR "Non-invasive monitoring device" OR "Noninvasive monitoring devices" OR "Noninvasive monitoring devices" OR "Non-invasive monitoring devices" OR "Noninvasive monitoring technologies" OR "Noninvasive monitoring technologies" OR "Non-invasive monitoring technologies" OR "Noninvasive monitoring technology" OR "Noninvasive monitoring technology" OR "Non-invasive monitoring technology" OR "Noninvasive monitoring" OR "Non-invasive monitoring" OR "Noninvasive technologies" OR "Non-invasive technologies" OR "Noninvasive technology" OR "Non-invasive technology" OR "Outpatient Monitor*" OR "Outpatient Monitoring" OR "Portable Electronic Application" OR "Portable Electronic Applications" OR "Portable Software Application" OR "Portable Software Applications" OR "Smart watch" OR "Smart watches" OR "Smartwatch" OR "Smartwatches" OR "tracker" OR "trackers" OR "wearab*" OR "wearable activity tracker" OR "wearable activity trackers" OR "wearable activity tracking" OR "Wearable Device" OR "Wearable Devices" OR "Wearable Electronic Device" OR "Wearable Electronic Devices" OR "Wearable Electronic Devices" OR "Wearable Technologies" OR "Wearable Technology" OR "wearable" OR "wearables" OR "noninvasive body sensor" OR "noninvasive body sensors" OR "non invasive body sensor" OR "non invasive body sensors" OR "body sensor" OR "body sensors" OR "wristband sensor" OR "wristband sensors" OR "wearable computing" OR "wearable computer" OR "wearable computers" OR "point-of-care App" OR "point-of-care Apps" OR "point-of-care Application" OR "point-of-care Applications" OR "facial recognition technology" OR "face recognition technology" OR "Point-of-Care Technology" OR "Point-of-Care Techn*" OR "HUME" OR "ePAT" OR " electronic pain assessment" OR ("BIS" AND "bispectral") OR "bispectral index" OR "Consciousness Monitors" OR "Biosensing Techniques" OR ("Facial Expression" AND "Point-of-Care Systems") OR (("noninvasiv*" OR "non invasiv*" OR "unobtrusiv*" OR "portable" OR "Mobile Applications") AND "monitor*") OR "Painchek" OR "Paincheck" OR "automated facial analysis" OR "automated facial recognition" OR "automated facial expression analysis") AND TI("discomfort" OR "discomfort*" OR "Psychological Distress" OR "distressing symptoms" OR "distressing symptom" OR "distressing" OR "distress" OR "Patient Comfort" OR "comfort" OR "comfort*" OR "Psychomotor Agitation" OR "agitation" OR "agitat*" OR "restlessness" OR "restless*" OR "hyperactivity" OR "hyperactiv*" OR "akathisia" OR "Pain" OR "pain" OR "Pain Measurement" OR "pain assessment" OR "Stress, Psychological" OR "stress level" OR "stress levels" OR "pressure ulcer" OR "pressure ulcer" OR "life quality" OR "quality of life" OR "quality of life" OR "breathing difficulty" OR "difficulty breathing" OR "difficult breathing" OR "labored breathing" OR "laboured breathing" OR "Shortness of Breath" OR "Breath Shortness" OR "Breathlessness" OR "Dyspnea" OR "Dyspnea" OR "Anxiety" OR "Anxiety" OR "Nervousness" OR "Hypervigilance" OR "Anxiousness" OR "Sleep Wake Disorders" OR "sleep disturbances" OR "sleep disturbance" OR "Fatigue" OR "fatigue" OR "Nausea" OR "nausea" OR "nauseous" OR "Delirium" OR "delirium" OR "delirious" OR "Fever" OR "fever" OR "Urinary Incontinence" OR "Incontinence" OR "incontinen*" OR "Sedation depth" OR "Sedation depths" OR "Depth of sedation")) OR (TI("Dementia" OR "Dementia" OR "dement*" OR "Alzheimer Disease" OR "Alzheimer*" OR "CADASIL" OR "Creutzfeldt-Jakob" OR "Creutzfeldt-Jakob Syndrome" OR "Diffuse Neurofibrillary Tangles with Calcification" OR "Frontotemporal Lobar Degeneration" OR "Huntington Disease" OR "Huntington*" OR "Kluver-Bucy" OR "Kluver-Bucy Syndrome" OR "Lewy Body" OR "Lewy Body Disease" OR "Pick Disease of the Brain" OR "Primary Progressive Aphasia" OR "Primary Progressive Nonfluent Aphasia" OR "Death" OR "dying" OR "end of life phase" OR "end of life" OR "end-of-life phase" OR "endoflife" OR "end-of-life" OR "Hospice and Palliative Care Nursing" OR "Hospice Care" OR "Hospice Care" OR "Hospice Care" OR "Hospice" OR "Hospices" OR "Hospices" OR "palliat*" OR "Palliative Care" OR "Palliative Care" OR "Palliative Medicine" OR "Palliative Phase" OR "Palliative Phases" OR "Palliative Stage" OR "Palliative Stages" OR "Palliative Supportive Care" OR "Palliative Surgery" OR "Palliative Therapy" OR "Palliative Treatment" OR "Palliative Treatments" OR "Terminal Care" OR "Terminal Care" OR "terminal stage" OR "elderly" OR "critical care" OR "critical care" OR "intensive care" OR "Critical Illness" OR "Critical Illness" OR "critically ill" OR (("profound" OR "profound*") AND ("Disabled Persons" OR "Intellectual Disability")) OR (("profound" OR "profound*") AND ("intellectual" OR "intellectualism" OR "intellectually" OR "intellectuals") AND ("multiple" OR "multiples") AND ("disabilities" OR "disability" OR "disabled persons" OR "disabled persons" OR "disabled" OR "disablement" OR "disablements" OR "disabling" OR "disablity")) OR "Bedridden Persons" OR "Bedridden" OR "Non-Mobile Person" OR "Non-Mobile Persons") AND TI("activity monitor" OR "activity monitors" OR "activity monitoring" OR "activity tracker" OR "activity trackers" OR "activity tracking" OR "Ambulatory Monitor*" OR "Ambulatory Monitoring" OR "Electronic Skin" OR (("monitor*" OR "monitoring") AND ("Telemedicine" OR "telemed*" OR "telehealth*" OR "technol*")) OR "monitoring app" OR "monitoring application" OR "monitoring applications" OR "monitoring apps" OR "monitoring device" OR "monitoring devices" OR "monitoring technologies" OR "monitoring technology" OR "Monitoring, Ambulatory" OR "Monitoring, Physiologic" OR "Noninvasive device" OR "Non-invasive device" OR "Noninvasive devices" OR "Non-invasive devices" OR "Noninvasive monitoring device" OR "Noninvasive monitoring device" OR "Non-invasive monitoring device" OR "Noninvasive monitoring devices" OR "Noninvasive monitoring devices" OR "Non-invasive monitoring devices" OR "Noninvasive monitoring technologies" OR "Noninvasive monitoring technologies" OR "Non-invasive monitoring technologies" OR "Noninvasive monitoring technology" OR "Noninvasive monitoring technology" OR "Non-invasive monitoring technology" OR "Noninvasive monitoring" OR "Non-invasive monitoring" OR "Noninvasive technologies" OR "Non-invasive technologies" OR "Noninvasive technology" OR "Non-invasive technology" OR "Outpatient Monitor*" OR "Outpatient Monitoring" OR "Portable Electronic Application" OR "Portable Electronic Applications" OR "Portable Software Application" OR "Portable Software Applications" OR "Smart watch" OR "Smart watches" OR "Smartwatch" OR "Smartwatches" OR "tracker" OR "trackers" OR "wearab*" OR "wearable activity tracker" OR "wearable activity trackers" OR "wearable activity tracking" OR "Wearable Device" OR "Wearable Devices" OR "Wearable Electronic Device" OR "Wearable Electronic Devices" OR "Wearable Electronic Devices" OR "Wearable Technologies" OR "Wearable Technology" OR "wearable" OR "wearables" OR "noninvasive body sensor" OR "noninvasive body sensors" OR "non invasive body sensor" OR "non invasive body sensors" OR "body sensor" OR "body sensors" OR "wristband sensor" OR "wristband sensors" OR "wearable computing" OR "wearable computer" OR "wearable computers" OR "point-of-care App" OR "point-of-care Apps" OR "point-of-care Application" OR "point-of-care Applications" OR "facial recognition technology" OR "face recognition technology" OR "Point-of-Care Technology" OR "Point-of-Care Techn*" OR "HUME" OR "ePAT" OR " electronic pain assessment" OR ("BIS" AND "bispectral") OR "bispectral index" OR "Consciousness Monitors" OR "Biosensing Techniques" OR ("Facial Expression" AND "Point-of-Care Systems") OR (("noninvasiv*" OR "non invasiv*" OR "unobtrusiv*" OR "portable" OR "Mobile Applications") AND "monitor*") OR "Painchek" OR "Paincheck" OR "automated facial analysis" OR "automated facial recognition" OR "automated facial expression analysis") AND TX("discomfort" OR "discomfort*" OR "Psychological Distress" OR "distressing symptoms" OR "distressing symptom" OR "distressing" OR "distress" OR "Patient Comfort" OR "comfort" OR "comfort*" OR "Psychomotor Agitation" OR "agitation" OR "agitat*" OR "restlessness" OR "restless*" OR "hyperactivity" OR "hyperactiv*" OR "akathisia" OR "Pain" OR "pain" OR "Pain Measurement" OR "pain assessment" OR "Stress, Psychological" OR "stress level" OR "stress levels" OR "pressure ulcer" OR "pressure ulcer" OR "life quality" OR "quality of life" OR "quality of life" OR "breathing difficulty" OR "difficulty breathing" OR "difficult breathing" OR "labored breathing" OR "laboured breathing" OR "Shortness of Breath" OR "Breath Shortness" OR "Breathlessness" OR "Dyspnea" OR "Dyspnea" OR "Anxiety" OR "Anxiety" OR "Nervousness" OR "Hypervigilance" OR "Anxiousness" OR "Sleep Wake Disorders" OR "sleep disturbances" OR "sleep disturbance" OR "Fatigue" OR "fatigue" OR "Nausea" OR "nausea" OR "nauseous" OR "Delirium" OR "delirium" OR "delirious" OR "Fever" OR "fever" OR "Urinary Incontinence" OR "Incontinence" OR "incontinen*" OR "Sedation depth" OR "Sedation depths" OR "Depth of sedation"))

## Academic Search Premier (EbscoHOST)

(AB("Dementia" OR "Dementia" OR "dement*" OR "Alzheimer Disease" OR "Alzheimer*" OR "CADASIL" OR "Creutzfeldt-Jakob" OR "Creutzfeldt-Jakob Syndrome" OR "Diffuse Neurofibrillary Tangles with Calcification" OR "Frontotemporal Lobar Degeneration" OR "Huntington Disease" OR "Huntington*" OR "Kluver-Bucy" OR "Kluver-Bucy Syndrome" OR "Lewy Body" OR "Lewy Body Disease" OR "Pick Disease of the Brain" OR "Primary Progressive Aphasia" OR "Primary Progressive Nonfluent Aphasia" OR "Death" OR "dying" OR "end of life phase" OR "end of life" OR "end-of-life phase" OR "endoflife" OR "end-of-life" OR "Hospice and Palliative Care Nursing" OR "Hospice Care" OR "Hospice Care" OR "Hospice Care" OR "Hospice" OR "Hospices" OR "Hospices" OR "palliat*" OR "Palliative Care" OR "Palliative Care" OR "Palliative Medicine" OR "Palliative Phase" OR "Palliative Phases" OR "Palliative Stage" OR "Palliative Stages" OR "Palliative Supportive Care" OR "Palliative Surgery" OR "Palliative Therapy" OR "Palliative Treatment" OR "Palliative Treatments" OR "Terminal Care" OR "Terminal Care" OR "terminal stage" OR "elderly" OR "critical care" OR "critical care" OR "intensive care" OR "Critical Illness" OR "Critical Illness" OR "critically ill" OR (("profound" OR "profound*") AND ("Disabled Persons" OR "Intellectual Disability")) OR (("profound" OR "profound*") AND ("intellectual" OR "intellectualism" OR "intellectually" OR "intellectuals") AND ("multiple" OR "multiples") AND ("disabilities" OR "disability" OR "disabled persons" OR "disabled persons" OR "disabled" OR "disablement" OR "disablements" OR "disabling" OR "disablity")) OR "Bedridden Persons" OR "Bedridden" OR "Non-Mobile Person" OR "Non-Mobile Persons") AND TI("activity monitor" OR "activity monitors" OR "activity monitoring" OR "activity tracker" OR "activity trackers" OR "activity tracking" OR "Ambulatory Monitor*" OR "Ambulatory Monitoring" OR "Electronic Skin" OR (("monitor*" OR "monitoring") AND ("Telemedicine" OR "telemed*" OR "telehealth*" OR "technol*")) OR "monitoring app" OR "monitoring application" OR "monitoring applications" OR "monitoring apps" OR "monitoring device" OR "monitoring devices" OR "monitoring technologies" OR "monitoring technology" OR "Monitoring, Ambulatory" OR "Monitoring, Physiologic" OR "Noninvasive device" OR "Non-invasive device" OR "Noninvasive devices" OR "Non-invasive devices" OR "Noninvasive monitoring device" OR "Noninvasive monitoring device" OR "Non-invasive monitoring device" OR "Noninvasive monitoring devices" OR "Noninvasive monitoring devices" OR "Non-invasive monitoring devices" OR "Noninvasive monitoring technologies" OR "Noninvasive monitoring technologies" OR "Non-invasive monitoring technologies" OR "Noninvasive monitoring technology" OR "Noninvasive monitoring technology" OR "Non-invasive monitoring technology" OR "Noninvasive monitoring" OR "Non-invasive monitoring" OR "Noninvasive technologies" OR "Non-invasive technologies" OR "Noninvasive technology" OR "Non-invasive technology" OR "Outpatient Monitor*" OR "Outpatient Monitoring" OR "Portable Electronic Application" OR "Portable Electronic Applications" OR "Portable Software Application" OR "Portable Software Applications" OR "Smart watch" OR "Smart watches" OR "Smartwatch" OR "Smartwatches" OR "tracker" OR "trackers" OR "wearab*" OR "wearable activity tracker" OR "wearable activity trackers" OR "wearable activity tracking" OR "Wearable Device" OR "Wearable Devices" OR "Wearable Electronic Device" OR "Wearable Electronic Devices" OR "Wearable Electronic Devices" OR "Wearable Technologies" OR "Wearable Technology" OR "wearable" OR "wearables" OR "noninvasive body sensor" OR "noninvasive body sensors" OR "non invasive body sensor" OR "non invasive body sensors" OR "body sensor" OR "body sensors" OR "wristband sensor" OR "wristband sensors" OR "wearable computing" OR "wearable computer" OR "wearable computers" OR "point-of-care App" OR "point-of-care Apps" OR "point-of-care Application" OR "point-of-care Applications" OR "facial recognition technology" OR "face recognition technology" OR "Point-of-Care Technology" OR "Point-of-Care Techn*" OR "HUME" OR "ePAT" OR " electronic pain assessment" OR ("BIS" AND "bispectral") OR "bispectral index" OR "Consciousness Monitors" OR "Biosensing Techniques" OR ("Facial Expression" AND "Point-of-Care Systems") OR (("noninvasiv*" OR "non invasiv*" OR "unobtrusiv*" OR "portable" OR "Mobile Applications") AND "monitor*") OR "Painchek" OR "Paincheck" OR "automated facial analysis" OR "automated facial recognition" OR "automated facial expression analysis") AND TI("discomfort" OR "discomfort*" OR "Psychological Distress" OR "distressing symptoms" OR "distressing symptom" OR "distressing" OR "distress" OR "Patient Comfort" OR "comfort" OR "comfort*" OR "Psychomotor Agitation" OR "agitation" OR "agitat*" OR "restlessness" OR "restless*" OR "hyperactivity" OR "hyperactiv*" OR "akathisia" OR "Pain" OR "pain" OR "Pain Measurement" OR "pain assessment" OR "Stress, Psychological" OR "stress level" OR "stress levels" OR "pressure ulcer" OR "pressure ulcer" OR "life quality" OR "quality of life" OR "quality of life" OR "breathing difficulty" OR "difficulty breathing" OR "difficult breathing" OR "labored breathing" OR "laboured breathing" OR "Shortness of Breath" OR "Breath Shortness" OR "Breathlessness" OR "Dyspnea" OR "Dyspnea" OR "Anxiety" OR "Anxiety" OR "Nervousness" OR "Hypervigilance" OR "Anxiousness" OR "Sleep Wake Disorders" OR "sleep disturbances" OR "sleep disturbance" OR "Fatigue" OR "fatigue" OR "Nausea" OR "nausea" OR "nauseous" OR "Delirium" OR "delirium" OR "delirious" OR "Fever" OR "fever" OR "Urinary Incontinence" OR "Incontinence" OR "incontinen*" OR "Sedation depth" OR "Sedation depths" OR "Depth of sedation")) OR (TI("Dementia" OR "Dementia" OR "dement*" OR "Alzheimer Disease" OR "Alzheimer*" OR "CADASIL" OR "Creutzfeldt-Jakob" OR "Creutzfeldt-Jakob Syndrome" OR "Diffuse Neurofibrillary Tangles with Calcification" OR "Frontotemporal Lobar Degeneration" OR "Huntington Disease" OR "Huntington*" OR "Kluver-Bucy" OR "Kluver-Bucy Syndrome" OR "Lewy Body" OR "Lewy Body Disease" OR "Pick Disease of the Brain" OR "Primary Progressive Aphasia" OR "Primary Progressive Nonfluent Aphasia" OR "Death" OR "dying" OR "end of life phase" OR "end of life" OR "end-of-life phase" OR "endoflife" OR "end-of-life" OR "Hospice and Palliative Care Nursing" OR "Hospice Care" OR "Hospice Care" OR "Hospice Care" OR "Hospice" OR "Hospices" OR "Hospices" OR "palliat*" OR "Palliative Care" OR "Palliative Care" OR "Palliative Medicine" OR "Palliative Phase" OR "Palliative Phases" OR "Palliative Stage" OR "Palliative Stages" OR "Palliative Supportive Care" OR "Palliative Surgery" OR "Palliative Therapy" OR "Palliative Treatment" OR "Palliative Treatments" OR "Terminal Care" OR "Terminal Care" OR "terminal stage" OR "elderly" OR "critical care" OR "critical care" OR "intensive care" OR "Critical Illness" OR "Critical Illness" OR "critically ill" OR (("profound" OR "profound*") AND ("Disabled Persons" OR "Intellectual Disability")) OR (("profound" OR "profound*") AND ("intellectual" OR "intellectualism" OR "intellectually" OR "intellectuals") AND ("multiple" OR "multiples") AND ("disabilities" OR "disability" OR "disabled persons" OR "disabled persons" OR "disabled" OR "disablement" OR "disablements" OR "disabling" OR "disablity")) OR "Bedridden Persons" OR "Bedridden" OR "Non-Mobile Person" OR "Non-Mobile Persons") AND AB("activity monitor" OR "activity monitors" OR "activity monitoring" OR "activity tracker" OR "activity trackers" OR "activity tracking" OR "Ambulatory Monitor*" OR "Ambulatory Monitoring" OR "Electronic Skin" OR (("monitor*" OR "monitoring") AND ("Telemedicine" OR "telemed*" OR "telehealth*" OR "technol*")) OR "monitoring app" OR "monitoring application" OR "monitoring applications" OR "monitoring apps" OR "monitoring device" OR "monitoring devices" OR "monitoring technologies" OR "monitoring technology" OR "Monitoring, Ambulatory" OR "Monitoring, Physiologic" OR "Noninvasive device" OR "Non-invasive device" OR "Noninvasive devices" OR "Non-invasive devices" OR "Noninvasive monitoring device" OR "Noninvasive monitoring device" OR "Non-invasive monitoring device" OR "Noninvasive monitoring devices" OR "Noninvasive monitoring devices" OR "Non-invasive monitoring devices" OR "Noninvasive monitoring technologies" OR "Noninvasive monitoring technologies" OR "Non-invasive monitoring technologies" OR "Noninvasive monitoring technology" OR "Noninvasive monitoring technology" OR "Non-invasive monitoring technology" OR "Noninvasive monitoring" OR "Non-invasive monitoring" OR "Noninvasive technologies" OR "Non-invasive technologies" OR "Noninvasive technology" OR "Non-invasive technology" OR "Outpatient Monitor*" OR "Outpatient Monitoring" OR "Portable Electronic Application" OR "Portable Electronic Applications" OR "Portable Software Application" OR "Portable Software Applications" OR "Smart watch" OR "Smart watches" OR "Smartwatch" OR "Smartwatches" OR "tracker" OR "trackers" OR "wearab*" OR "wearable activity tracker" OR "wearable activity trackers" OR "wearable activity tracking" OR "Wearable Device" OR "Wearable Devices" OR "Wearable Electronic Device" OR "Wearable Electronic Devices" OR "Wearable Electronic Devices" OR "Wearable Technologies" OR "Wearable Technology" OR "wearable" OR "wearables" OR "noninvasive body sensor" OR "noninvasive body sensors" OR "non invasive body sensor" OR "non invasive body sensors" OR "body sensor" OR "body sensors" OR "wristband sensor" OR "wristband sensors" OR "wearable computing" OR "wearable computer" OR "wearable computers" OR "point-of-care App" OR "point-of-care Apps" OR "point-of-care Application" OR "point-of-care Applications" OR "facial recognition technology" OR "face recognition technology" OR "Point-of-Care Technology" OR "Point-of-Care Techn*" OR "HUME" OR "ePAT" OR " electronic pain assessment" OR ("BIS" AND "bispectral") OR "bispectral index" OR "Consciousness Monitors" OR "Biosensing Techniques" OR ("Facial Expression" AND "Point-of-Care Systems") OR (("noninvasiv*" OR "non invasiv*" OR "unobtrusiv*" OR "portable" OR "Mobile Applications") AND "monitor*") OR "Painchek" OR "Paincheck" OR "automated facial analysis" OR "automated facial recognition" OR "automated facial expression analysis") AND TI("discomfort" OR "discomfort*" OR "Psychological Distress" OR "distressing symptoms" OR "distressing symptom" OR "distressing" OR "distress" OR "Patient Comfort" OR "comfort" OR "comfort*" OR "Psychomotor Agitation" OR "agitation" OR "agitat*" OR "restlessness" OR "restless*" OR "hyperactivity" OR "hyperactiv*" OR "akathisia" OR "Pain" OR "pain" OR "Pain Measurement" OR "pain assessment" OR "Stress, Psychological" OR "stress level" OR "stress levels" OR "pressure ulcer" OR "pressure ulcer" OR "life quality" OR "quality of life" OR "quality of life" OR "breathing difficulty" OR "difficulty breathing" OR "difficult breathing" OR "labored breathing" OR "laboured breathing" OR "Shortness of Breath" OR "Breath Shortness" OR "Breathlessness" OR "Dyspnea" OR "Dyspnea" OR "Anxiety" OR "Anxiety" OR "Nervousness" OR "Hypervigilance" OR "Anxiousness" OR "Sleep Wake Disorders" OR "sleep disturbances" OR "sleep disturbance" OR "Fatigue" OR "fatigue" OR "Nausea" OR "nausea" OR "nauseous" OR "Delirium" OR "delirium" OR "delirious" OR "Fever" OR "fever" OR "Urinary Incontinence" OR "Incontinence" OR "incontinen*" OR "Sedation depth" OR "Sedation depths" OR "Depth of sedation")) OR (TI("Dementia" OR "Dementia" OR "dement*" OR "Alzheimer Disease" OR "Alzheimer*" OR "CADASIL" OR "Creutzfeldt-Jakob" OR "Creutzfeldt-Jakob Syndrome" OR "Diffuse Neurofibrillary Tangles with Calcification" OR "Frontotemporal Lobar Degeneration" OR "Huntington Disease" OR "Huntington*" OR "Kluver-Bucy" OR "Kluver-Bucy Syndrome" OR "Lewy Body" OR "Lewy Body Disease" OR "Pick Disease of the Brain" OR "Primary Progressive Aphasia" OR "Primary Progressive Nonfluent Aphasia" OR "Death" OR "dying" OR "end of life phase" OR "end of life" OR "end-of-life phase" OR "endoflife" OR "end-of-life" OR "Hospice and Palliative Care Nursing" OR "Hospice Care" OR "Hospice Care" OR "Hospice Care" OR "Hospice" OR "Hospices" OR "Hospices" OR "palliat*" OR "Palliative Care" OR "Palliative Care" OR "Palliative Medicine" OR "Palliative Phase" OR "Palliative Phases" OR "Palliative Stage" OR "Palliative Stages" OR "Palliative Supportive Care" OR "Palliative Surgery" OR "Palliative Therapy" OR "Palliative Treatment" OR "Palliative Treatments" OR "Terminal Care" OR "Terminal Care" OR "terminal stage" OR "elderly" OR "critical care" OR "critical care" OR "intensive care" OR "Critical Illness" OR "Critical Illness" OR "critically ill" OR (("profound" OR "profound*") AND ("Disabled Persons" OR "Intellectual Disability")) OR (("profound" OR "profound*") AND ("intellectual" OR "intellectualism" OR "intellectually" OR "intellectuals") AND ("multiple" OR "multiples") AND ("disabilities" OR "disability" OR "disabled persons" OR "disabled persons" OR "disabled" OR "disablement" OR "disablements" OR "disabling" OR "disablity")) OR "Bedridden Persons" OR "Bedridden" OR "Non-Mobile Person" OR "Non-Mobile Persons") AND TI("activity monitor" OR "activity monitors" OR "activity monitoring" OR "activity tracker" OR "activity trackers" OR "activity tracking" OR "Ambulatory Monitor*" OR "Ambulatory Monitoring" OR "Electronic Skin" OR (("monitor*" OR "monitoring") AND ("Telemedicine" OR "telemed*" OR "telehealth*" OR "technol*")) OR "monitoring app" OR "monitoring application" OR "monitoring applications" OR "monitoring apps" OR "monitoring device" OR "monitoring devices" OR "monitoring technologies" OR "monitoring technology" OR "Monitoring, Ambulatory" OR "Monitoring, Physiologic" OR "Noninvasive device" OR "Non-invasive device" OR "Noninvasive devices" OR "Non-invasive devices" OR "Noninvasive monitoring device" OR "Noninvasive monitoring device" OR "Non-invasive monitoring device" OR "Noninvasive monitoring devices" OR "Noninvasive monitoring devices" OR "Non-invasive monitoring devices" OR "Noninvasive monitoring technologies" OR "Noninvasive monitoring technologies" OR "Non-invasive monitoring technologies" OR "Noninvasive monitoring technology" OR "Noninvasive monitoring technology" OR "Non-invasive monitoring technology" OR "Noninvasive monitoring" OR "Non-invasive monitoring" OR "Noninvasive technologies" OR "Non-invasive technologies" OR "Noninvasive technology" OR "Non-invasive technology" OR "Outpatient Monitor*" OR "Outpatient Monitoring" OR "Portable Electronic Application" OR "Portable Electronic Applications" OR "Portable Software Application" OR "Portable Software Applications" OR "Smart watch" OR "Smart watches" OR "Smartwatch" OR "Smartwatches" OR "tracker" OR "trackers" OR "wearab*" OR "wearable activity tracker" OR "wearable activity trackers" OR "wearable activity tracking" OR "Wearable Device" OR "Wearable Devices" OR "Wearable Electronic Device" OR "Wearable Electronic Devices" OR "Wearable Electronic Devices" OR "Wearable Technologies" OR "Wearable Technology" OR "wearable" OR "wearables" OR "noninvasive body sensor" OR "noninvasive body sensors" OR "non invasive body sensor" OR "non invasive body sensors" OR "body sensor" OR "body sensors" OR "wristband sensor" OR "wristband sensors" OR "wearable computing" OR "wearable computer" OR "wearable computers" OR "point-of-care App" OR "point-of-care Apps" OR "point-of-care Application" OR "point-of-care Applications" OR "facial recognition technology" OR "face recognition technology" OR "Point-of-Care Technology" OR "Point-of-Care Techn*" OR "HUME" OR "ePAT" OR " electronic pain assessment" OR ("BIS" AND "bispectral") OR "bispectral index" OR "Consciousness Monitors" OR "Biosensing Techniques" OR ("Facial Expression" AND "Point-of-Care Systems") OR (("noninvasiv*" OR "non invasiv*" OR "unobtrusiv*" OR "portable" OR "Mobile Applications") AND "monitor*") OR "Painchek" OR "Paincheck" OR "automated facial analysis" OR "automated facial recognition" OR "automated facial expression analysis") AND AB("discomfort" OR "discomfort*" OR "Psychological Distress" OR "distressing symptoms" OR "distressing symptom" OR "distressing" OR "distress" OR "Patient Comfort" OR "comfort" OR "comfort*" OR "Psychomotor Agitation" OR "agitation" OR "agitat*" OR "restlessness" OR "restless*" OR "hyperactivity" OR "hyperactiv*" OR "akathisia" OR "Pain" OR "pain" OR "Pain Measurement" OR "pain assessment" OR "Stress, Psychological" OR "stress level" OR "stress levels" OR "pressure ulcer" OR "pressure ulcer" OR "life quality" OR "quality of life" OR "quality of life" OR "breathing difficulty" OR "difficulty breathing" OR "difficult breathing" OR "labored breathing" OR "laboured breathing" OR "Shortness of Breath" OR "Breath Shortness" OR "Breathlessness" OR "Dyspnea" OR "Dyspnea" OR "Anxiety" OR "Anxiety" OR "Nervousness" OR "Hypervigilance" OR "Anxiousness" OR "Sleep Wake Disorders" OR "sleep disturbances" OR "sleep disturbance" OR "Fatigue" OR "fatigue" OR "Nausea" OR "nausea" OR "nauseous" OR "Delirium" OR "delirium" OR "delirious" OR "Fever" OR "fever" OR "Urinary Incontinence" OR "Incontinence" OR "incontinen*" OR "Sedation depth" OR "Sedation depths" OR "Depth of sedation"))

## Google Scholar

"Dementia"|"dying"|"end of life"|"Palliative"|"Hospice"|"critical care"|"Bedridden" "activity monitor"|"activity tracker"|"monitoring app" "discomfort"|"Distress"|"comfort"|"pain"
